# Root exudate composition of grass and forb species in natural grasslands

**DOI:** 10.1038/s41598-019-54309-5

**Published:** 2020-07-01

**Authors:** Sophie Dietz, Katharina Herz, Karin Gorzolka, Ute Jandt, Helge Bruelheide, Dierk Scheel

**Affiliations:** 1 Leibniz Institute of Plant Biochemistry, Weinberg 3, 06120 Halle (Saale), Germany; 2 Martin Luther University Halle-Wittenberg, Institute of Biology/Geobotany and Botanical Garden, Am Kirchtor 1, 06108 Halle [Saale], Germany; 3 German Centre for Integrative Biodiversity Research (iDiv) Halle-Jena-Leipzig, Deutscher Platz 5e, 04103 Leipzig, Germany

**Keywords:** Plant ecology, Ecosystem ecology

## Abstract

Plants exude a diverse cocktail of metabolites into the soil as response to exogenous and endogenous factors. So far, root exudates have mainly been studied under artificial conditions due to methodological difficulties. In this study, each five perennial grass and forb species were investigated for polar and semi-polar metabolites in exudates under field conditions. Metabolite collection and untargeted profiling approaches combined with a novel classification method allowed the designation of 182 metabolites. The composition of exuded polar metabolites depended mainly on the local environment, especially soil conditions, whereas the pattern of semi-polar metabolites was primarily affected by the species identity. The profiles of both polar and semi-polar metabolites differed between growth forms, with grass species being generally more similar to each other and more responsive to the abiotic environment than forb species. This study demonstrated the feasibility of investigating exudates under field conditions and to identify the driving factors of exudate composition.

## Introduction

Plants adjust their phenotype in response to environmental conditions and interactions with other organisms^1–4^. So far, studies on phenotypic adjustment have mainly focused on morphological and physiological characteristics. One result was that the species’ phenotypic plasticity differs between forbs or grasses, which are the dominating growth form of grasslands. These two growth forms vary with respect to plant functional traits^5–9^. Grasses are thereby the dominant organisms in grasslands due to their tolerance to mowing and grazing, fast spread and responses to environmental changes^5,8^. Forbs instead are less affected by changes in nutrient conditions than grasses due to their ability to store nutrients in their roots^8^. However, it is unknown so far whether these two growth forms also differ with respect to another fundamental plants phenotype: the metabolites exuded by plant roots.

Exudates comprise organic compounds of low molecular weight^10^ from the primary and secondary metabolism spanning a wide range of polarities. Examples for exuded primary metabolites are alcohols, amino acids, organic acids, carbohydrates, nucleic bases and nucleotides, exuded secondary metabolites are for example alkaloids, phenylpropanoids and terpenes^3,11–14^. Due to the wide range of polarity, distinct analytical methods need to be applied to cover a broad range of detected compounds, e.g. gas chromatography coupled to mass spectrometry (GC-MS) and reversed phase liquid chromatography coupled to mass spectrometry (RP-LC-MS).

Various experiments revealed that the composition of the exuded metabolites into the rhizosphere^1,15–18^ depends on different endogenous and exogenous factors. Endogenous factors such as growth form^19^, species^2,3,20^, and plant functional traits^19–21^ turned out to alter the exudate composition in the rhizosphere. In addition, exogenous biotic factors e.g. microbial rhizosphere community^16^, herbivores^15^ and neighbouring plants^4,22,23^ also define the metabolite profile. Rice plants inhibit the growth of neighbouring lettuce and grass species by the release of lactones^23^. The noxious weed (*Centaurea maculosa*) secretes the allelochemical (±)-catechin into the surrounding rhizosphere to delay germination of seeds and thus prevents intraspecific sibling competition^22^. However, studies investigating the effect of the neighbouring plants on the exudate patterns of target plants in a natural environment are rare so far^19,21,24^.

Besides responding to biotic factors, root metabolites are also released as a reaction to the abiotic environment such as light and temperature, soil pH, moisture, nutrient supply and organic matter^25–31^. For example, nutrient deficiency often results from a complex formation with metal ions or absorption by the soil^31,32^ which can be overcome by the release of organic acids^33^ or phenolic compounds from the plant^30,31^. These chelating substances enhance the acquisition of insoluble nutrients and interfere with the nutrient cycles^25^. Furthermore, anthropogenic land use was assumed to influence the exudate profiles of plants^19^. High amounts of nutrients, such as nitrogen and phosphor, are introduced into the soil by fertilization and grazing. This modifies the soil nutrient status^34–36^ and, thus, probably the root exudation. However, this correlation has not yet been fully analysed.

Most of the knowledge about exudates were obtained under controlled laboratory conditions, some of those mimic the natural ecosystem conditions^24,37^, with one- or two-factorial designs^4,16,22,23,38,39^. Furthermore, due to the tremendous variety of metabolites in the plant kingdom^3,40,41^, these studies have mainly focused on specific metabolites or metabolite classes^4,14,24^. As a result, a large part of the exuded plant metabolome remains unconsidered. On the other side, most studies under field conditions neglect the role of exudates. Thus, both types of strategies deliberately disregarded important components of the complex natural ecosystems. To fully understand those networks, a comprehensive investigation of the metabolite profile of a plant and the combination of metabolomics and ecological techniques under natural conditions are of great importance^40^. So far, there are two studies about root exudation of either polar^19^ or semi-polar root metabolites^21^ applying the untargeted metabolite profiling approach to field grown plants. These two studies focus mainly on the impacts of different endogenous factors in a natural ecosystem.

In this study, the effects of different exogenous factors such as climate, soil, neighbouring plants and anthropogenic land use, as well as the endogenous factors species and growth form were investigated for their impact on the composition of polar and semi-polar root exuded metabolites. Those were analysed by GC-MS (detecting more the polar fraction of metabolites) and C18-RP-LC-MS (targeting more the semi-polar fraction of metabolites), respectively. A large field experiment was performed in which five grass and five forb species were transplanted in more than 50 different grassland communities in the three sites of the German Biodiversity Exploratories (Schorfheide-Chorin, Hainich and Schwabian Alb). Those differ in various environmental factors e.g. soil, climate and land use^36,42,43^. After more than one year in the surrounding environment, the root exudates of the transplants were analysed by untargeted metabolite profiling. Moreover, as the identification of semi-polar metabolites is challenging due to their high chemical diversity in plants^14^, a novel approach of classifying metabolites to chemical classes was applied^44^.

The main issues of this paper are:

(1) Which of the factors growth form (grass or forb), species identity and site affect the root exudate richness under field conditions significantly?

(2) What is the impact of biotic growth conditions, species identity and neighbouring plants on root exudate composition?

## Results

### Chemical richness and composition of metabolites detected by GC-MS (polar metabolites)

The untargeted metabolite profiling of the investigated samples revealed an annotation of 285 features (detected monoisotopic signals characterized by their specific retention time and mass to charge ration) (Supplementary Table 1). A total of 66 of these features were identified and classified as metabolites of the classes alcohols (6), aldehydes (1), alkaloids (1), amines (2), amino acids (19), carbohydrates (10), lipids (4), nucleic bases or nucleotides (3) and organic acids (19). Five compounds were classified as unidentified carbohydrates (4) and unidentified lipid (1), respectively, due to their mass spectra similarity to other compounds of these classes (Table 1, Supplementary Table 1). The identified compounds in this GC-MS analysis were mainly of the primary metabolism and often of polar character. Thus GC-MS detected compounds and metabolites will be referred to as polar metabolites in the following sections.

**Table 1:** Overview of the polar metabolites.

		alkaloid (1)	alcohol (6)	aldehyde (1)	amine (2)	amino acid (19)	carbohydrates (10)	lipid (4)	nuclic base/nucleotide (3)	organic acid (18)	phenylpropanoid (1)	u_carbohydrates (4)	u_lipid (1)	u_compounds (215)
forb	ALB	0	139	0	1	266	160	7	7	219	0	129	19	1260
	HAI	0	181	2	0	369	219	13	15	314	1	118	21	2184
	SCH	0	80	0	0	189	82	23	15	140	0	61	16	1242
grass	ALB	1	140	2	1	371	153	17	22	232	0	123	28	1105
	HAI	0	114	0	1	343	127	9	24	206	0	84	20	1161
	SCH	0	104	0	1	385	116	42	37	235	0	81	26	2004
*A. millefolium*	ALB	0	32	0	0	61	34	2	3	42	0	22	4	227
	HAI	0	35	0	0	81	40	4	5	58	0	26	5	419
	SCH	0	10	0	0	22	11	3	2	16	0	9	2	155
*G. mollugo*	ALB	0	32	0	0	67	38	1	4	53	0	24	3	298
	HAI	0	38	1	0	99	50	2	3	75	1	26	3	504
	SCH	0	25	0	0	64	27	8	6	43	0	17	6	402
*G. verum*	ALB	0	29	0	0	57	37	2	0	47	0	32	7	246
	HAI	0	36	0	0	85	53	1	1	70	0	23	4	436
	SCH	0	18	0	0	44	21	5	6	34	0	9	4	257
*P. lanceolata*	ALB	0	25	0	1	44	31	1	0	48	0	35	2	324
	HAI	0	31	1	0	29	33	4	0	48	0	24	5	421
	SCH	0	17	0	0	42	17	6	1	31	0	20	3	282
*R. acris*	ALB	0	21	0	0	37	20	1	0	29	0	16	3	165
	HAI	0	41	0	0	75	43	2	6	64	0	19	4	404
	SCH	0	10	0	0	17	6	1	0	16	0	6	1	146
*A. praten-sis*	ALB	0	11	1	0	31	12	0	3	18	0	11	1	93
	HAI	0	19	0	0	73	28	3	4	41	0	18	5	238
	SCH	0	13	0	0	46	14	4	4	30	0	11	3	249
*elatius*	ALB	1	48	0	0	123	48	8	10	81	0	32	9	420
	HAI	0	16	0	0	49	19	1	2	25	0	12	2	148
	SCH	0	18	0	0	75	25	10	8	45	0	13	4	391
*D. glomerata*	ALB	0	32	1	0	92	38	5	4	57	0	37	10	265
	HAI	0	33	0	0	98	32	1	9	57	0	22	6	304
	SCH	0	28	0	1	117	33	13	13	70	0	23	9	601
*L. perenne*	ALB	0	27	0	0	60	29	2	2	41	0	23	5	158
	HAI	0	23	0	1	67	24	3	6	44	0	14	4	250
	SCH	0	26	0	0	88	26	8	7	54	0	19	5	464
*P pratensis*	ALB	0	22	0	1	65	26	2	3	35	0	20	3	169
	HAI	0	23	0	0	56	24	1	3	39	0	18	3	221
	SCH	0	19	0	0	59	18	7	5	36	0	15	5	299

The table shows the number of detections of a compounds of each class per site and species as well as site and growth form. The numbers in brackets represent the number of metabolite identities in each chemical class. u = unidentified.

**Figure 1:**
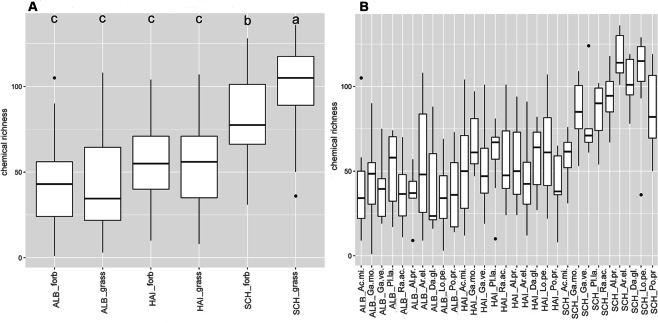
Chemical richness of polar metabolites. Boxplots display the median chemical richness (line in the box), the distribution (box) and the upper and lower quantile (lines) of the chemical richness of GC-MS detected (polar) metabolites of samples within the sample groups (**A)** site-growth form and (**B)** site-species. Points above and below the box represent outliers. A Scheffé post hoc test was performed to reveal significant differences between the sample groups. Sample groups with the same letter are not significantly different. Number of samples per group is given in Supplementary Table 9.

**Figure 2:**
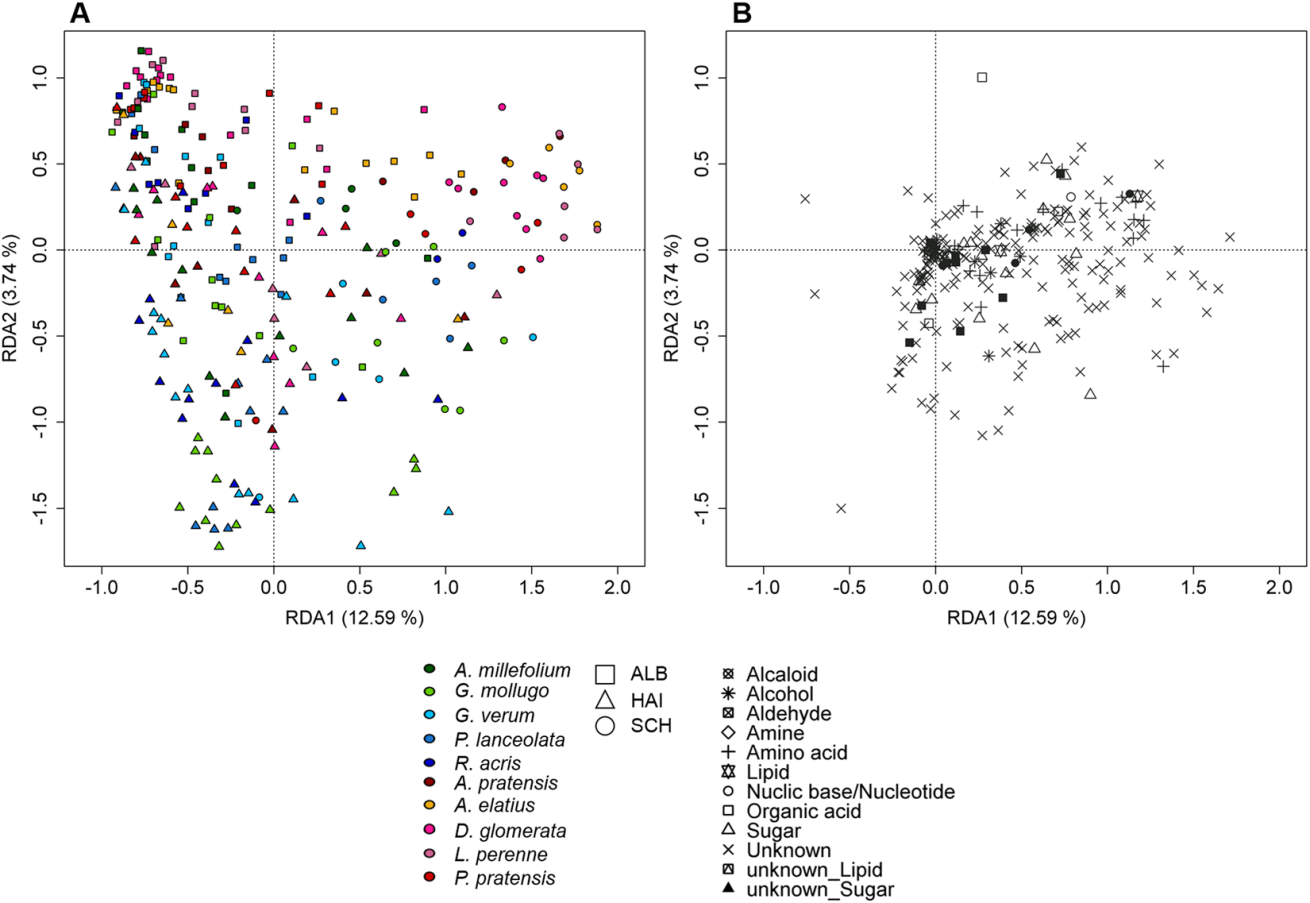
Redundancy analysis of polar metabolites. RDA was performed with 257 samples plotted against a presence/absence matrix of species per site. (**A)** presents axis 1 against axis 2. The ten species are indicated by colour, whereas the points are grouped by site (see legend). (**B)** represents the loadings of the exuded compounds of the corresponding RDA levels. The points represent the different chemical classes (see legend).

Linear mixed-effects models showed that the number of exuded metabolites (chemical richness, Fig. 1) significantly depended on species (*p* < 0.05), site (*p < *0.001; Schorfheide, Hainich or Swabian Alb) and their interaction (*p < *0.01; Supplementary Table 2a)). In contrast, the two growth forms grass and forb as a whole did not differ in their total number of exuded compounds (chemical richness), whereas those of forbs and grasses of the sites differ significantly from each other (*p < *0.001; Fig. 1A, Supplementary Table 2a)). Chemical richness was highest in the Schorfheide (SCH), in particular for grasses (*p* < 0.001) and the forb *Ranunculus acris* (*p < *0.01; Fig. 1A, Supplementary Table 2b)). The pattern of chemical richness was also reflected in the multivariate analysis of the metabolites composition, as revealed by a Redundancy Analysis (RDA, Fig. 2A) with species and site as constraining variables. Here, the samples of SCH were separated from those of HAI and ALB on first axis (12.59% of total variance explained, Fig. 2A), while ALB and HAI differed in their scores on the second axis (2.57%; Fig. 2A). In contrast to site, differences among species or growth forms played a subordinate role, and were only apparent on the lower axes (Supplementary Fig. 1).

The loadings of the exuded compounds (Fig. 2B) indicated a common set of metabolites in the exudate profiles of the transplants. Those comprised metabolites of the classes alcohols, amino acids, carbohydrates, lipids, nucleic bases, organic acids, and also unidentified compounds. Some of the class members, however, showed a higher probability to occur in specific sample groups. Grasses exuded the highest number of group discriminating metabolites compared to forbs (Supplementary Table 3a)). The highest number of those metabolites was observed in plots of the ALB such as N-acetylglucosamine, favourably exuded by *Poa pratensis*, and succinate, especially exuded by *Lolium perenne*, as well as a number of unidentified compounds (Supplementary Table 3).

Discriminating exudates of grass plants grown in SCH or HAI plots showed no species-specificity, but the number of those metabolites was higher in SCH than in HAI. Instead, forb exudate profiles revealed the highest number of discriminating metabolites in plots of the HAI. Most of those could be related to a specific species, e.g. 3-Caffeoyl-trans quinic acid preferentially exuded by *Galium mollugo*, and many unidentified compounds (Supplementary Table 3). Forbs grown on SCH and ALB plots exuded nearly the same number of discriminating metabolites. In all sample groups, many metabolites were preferentially exuded by a specific growth form in a specific site, but not by a specific species. Simultaneously, some compounds were preferentially exuded by plants of a specific growth form or species without an influence of the site factor. For instance, octadecatrienoic acid is exuded by *Plantago lanceolata* of all plots, whereas 2-aminoadipate is preferentially exuded by *A. elatius* plants in all plots (Supplementary Table 3).

### Polar metabolite composition and exogenous factors

**Figure 3:**
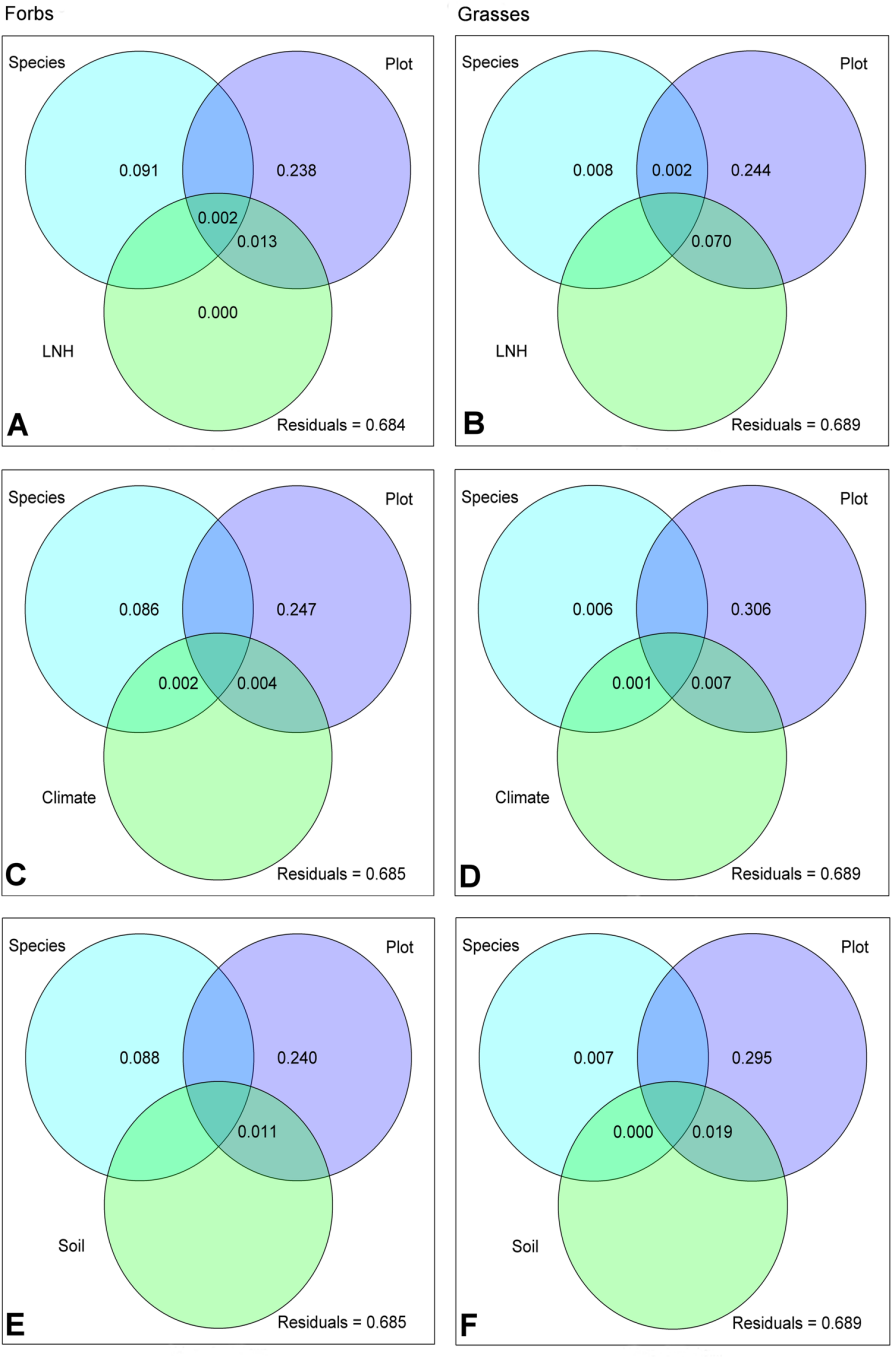
Variance partitioning of GC-MS detected (polar) metabolites. Venn diagrams present the proportion of variance in metabolite patterns of forbs (left) and grasses (right) explained by different predictors: Species = species identity of the target plant, Plot = location impact, LNH **(A,B)** = plant local neighbourhood community in a radius of 25 cm around the target plant, climate **(C,D)** = combined characteristics describing the temperature and precipitation, soil **(E,F)** = combined characteristics describing the soil of the location where the target plant was growing.

The importance of the growth location (e.g. the site) compared to species is also reflected in the variance partitioning analysis both for forbs and grasses (Fig. 3). Here, plot explained most of the variance (forbs: 23.8%, grasses: 24.4%; Fig. 3A, B). While in forbs (Fig. 3A) the second most important factor was species (8.8%), in grasses it was the interaction with the local neighbouring plants (LNH) and the plot (7.0%) together. The effect of LNH on polar metabolite composition was mainly brought about by covered area of the neighbouring plants (Cover, Supplementary Table 4). When LNH was replaced by environmental factors, either by climate and soil factors (Env, Supplementary Fig. 2, Supplementary Table 4), or by climate (Climate) and soil (Soil) separately (Fig. 3C–F), the previously described pattern remained essentially the same. Also here, plot explained most of the variation in the exudate metabolite pattern (24.0% to 24.7% for forbs, 29.5% to 30.6% for grasses), while neither climate nor soil were important at all. With minor exceptions, the same holds true for impacts of the factor land use and the single variables cumulated in the environmental factors (Supplementary Table 4). The highest amount of shared variation between plot and single variables was brought about by the combination of plot and total carbon content of the soil (TC; 2.85% and 7.38% in case of forbs and grasses, respectively; Supplementary Table 4).

The correlation of single metabolites to the different environmental drivers revealed that 65.14% and 69.72% of metabolites in forb and grass exudate samples responded significantly to the environment (Supplementary Table 5a,c)). Soil variables showed the highest number of impacted metabolites followed by LNH and climate, whereas the lowest number of metabolites is linked to LUI effects in forbs and grasses (Supplementary Fig. 3, Supplementary Table 5a,c)). For instance, 101 of the polar metabolites exuded by forbs and grasses were significantly affected by soil moisture (moisture), whereas the number (Richness) and the diversity (Shannon) of species neighbouring the exuded plant and the annual temperature in 2 m height above the plant (T(200)) affected the exudation of about 80 metabolites (Supplementary Fig. 3, Supplementary Table 5a). In accordance with the results of the variance partitioning and in all LUI variables, grazing affected primary metabolites to the highest extent.

### Semi-polar metabolites detected by LC-MS occur in a species-dependent manner in exudates

**Figure 4:**
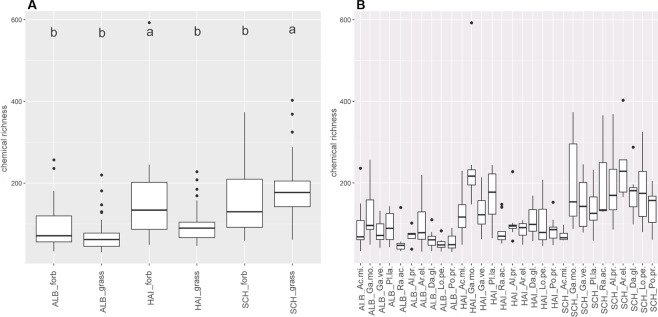
Chemical richness of semi-polar metabolites. Boxplots display the median chemical richness (line in the box), its distribution (box),quantiles (lines) and outlier (points) of the chemical richness of semi-polar metabolites of samples within the sample groups (**A)** site-growth form and (**B)** site-species. A Scheffé post hoc test was performed to reveal significant differences between the sample groups. Sample groups with the same letter are not significantly different. Number of samples per group is given in Supplementary Table 9.

**Figure 5:**
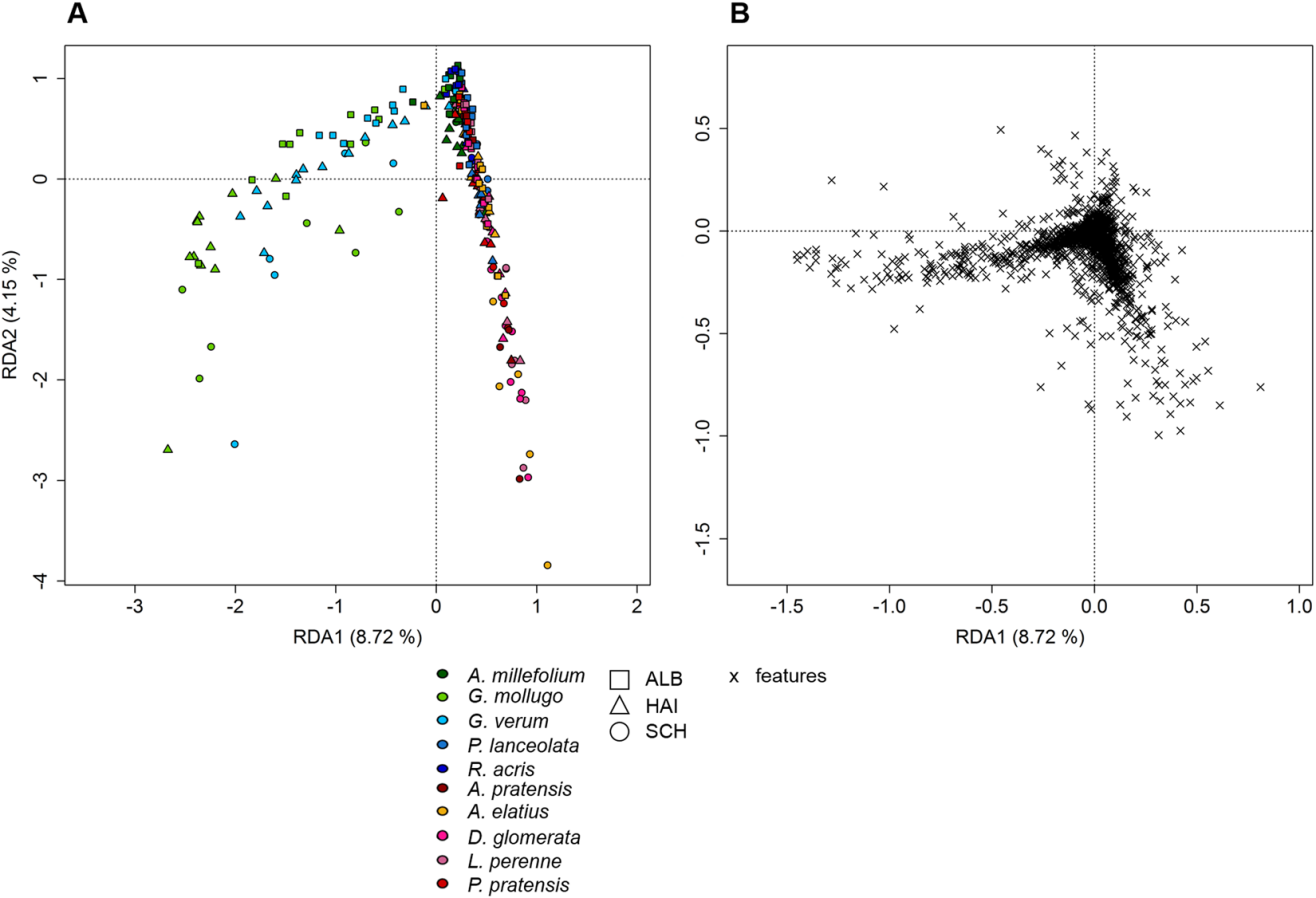
Redundancy analysis of semi-polar metabolites. RDA was performed with 257 samples plotted against a presence/absence matrix of species per site. (**A)** The ten species (*p = *0.001) are represented by colour, whereas the points are grouped by site (see legend). The plot (**B)** represents the loadings of semi-polar compounds.

Untargeted metabolite profiling by LC-MS (focussing on the semi-polar metabolite fraction) revealed 2,947 features. The chemical richness of the transplant exudate profiles was independent of the growth form (p = 0.630), but significantly depended on site and species (p < 0.001, Supplementary Table 2). This was mainly driven by the grass species, which displayed a higher chemical richness in SCH plots than in all other exploratory plots (Fig. 4B, Supplementary Table 2b)). Moreover exudates from grasses and forbs grown on SCH plots showed a significantly higher chemical richness than in the other exploratories (Fig. 4A, Supplementary Table 2b). For forbs, this was mainly driven by the *Galium* species.The RDA of semi-polar features partly reflected these results (Fig. 5). Although a discrimination of the plant samples by site was not observed, a species-specific pattern occurred (Fig. 5A). While the two Galium species were separated from the other species on axis one (8.72%), *P. lanceolata* was separated from the other species on axis three (2.42%, Supplementary Fig. 4). Furthermore, *A. millefolium* samples were discriminated from the other species on axis four (1.71%, Supplementary Fig. 4) whereas the separation of *R. acris* samples occurred on axis six (1.05%, Supplementary Fig. 4). Axis five (1.44%, Supplementary Fig. 4) was the only dimension in which *A. elatius*, a grass, was separated from all other species, while all other grass species always clustered together. The loadings of the exuded semi-polar metabolites (Fig. 5B) indicated a common set of exuded metabolites of all species, but also a higher degree of metabolite diversity in exudate patterns of forbs than of grasses. This is reflected by the calculation of the significant specific features per species or the genus *Galium* (*Galium* spp.) together, respectively. 229 of these significant species-specific features were thereby observed in metabolite profiles of forbs (*A. millefolium*: 40, *G. mollugo*: 69, *G. verum*: 2, *P. lanceolata*: 89, *R. acris*: 29), whereas 47 were observed in grass profiles. Further 76 significantly specific features were discovered in *Galium* spp. samples.

### Chemical classification of species-specific exuded semi-polar metabolites

**Figure 6:**
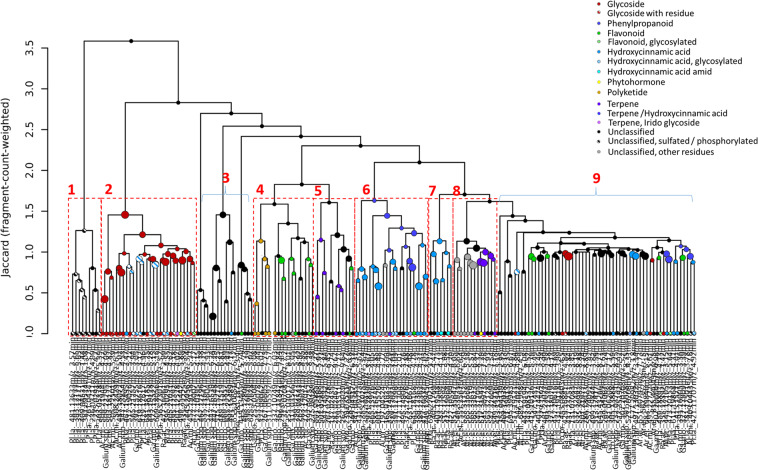
Hierarchical clustering of species-specific semi-polar exudates. Hierarchical clustering was performed on the tandem-mass spectra of the significant species-specific compounds. Cluster were calculated on spectral similarity rested on Jaccard dissimilarity and fragment-count-weighted value rating. The numbers represent the cluster which are shown in Supplementary Figs. 5–12 for more details. The classification of metabolites is given in the legend.

Tandem mass spectrometry (MS/MS) provided fragment mass spectra of 217 of the 352 significant species-specific features (Supplementary Table 6). Their chemical classification revealed 116 compounds grouped into seven chemical classes with different subclasses (Table 2): glycosides (20) with different residues (acid (2), sulfate (2), hydroxycarbonic acid (1)), jasmonate derivatives (2), phenylpropanoids such a coumarin derivative (1), flavonoids (15) (glycosylated (3), kaempferol derivatives (3)), hydroxycinnamic acids (39) (glycosylated (15), not glycosylated (21) amide residues (3)), polyketides (5), terpenes (12) and compounds which could not be assigned to one of these semi-polar metabolite families but carried different chemical residues (aliphatic (7), imine residues (2), methoxy-groups (3), sulfates or phosphate groups (10)). Hierarchical clustering of the compounds according to their mass spectral fragment similarities resulted in a dendrogram with nine main branches (Fig. 6). Seven of those corresponded to the substance classes listed above, whereas two branches contained members of all chemical families and unclassified compounds (Fig. 6, Supplementary Figs. 5–12). Furthermore, the spectra of species-specific compounds showed a clustering due to species identity. Sulfated or phosphorylated compounds clustered in branch one together and were predominantly exuded by *P. lanceolata* (Fig. 6, Supplementary Fig. 5). The majority of glycosylated compounds and glycosides clustered in main branch two and were exuded by *A. millefolium*, *G. mollugo*, *G. verum*, *P. lanceolata*, *A. elatius*, *A. pratensis*, and *R. acris* plants, respectively (Fig. 6, Supplementary Fig. 6). The annotated polyketides and some potential flavonoids released by *G. mollugo*, *G. verum*, *Galium* spp and *A. millefolium* roots clustered in branch four (Fig. 6, Supplementary Fig. 8). There were two branches containing substances that resembled terpenes (Fig. 6, Supplementary Figs. 9 and 11), mainly exuded by *Galium* spp (branch 5) or *A. elatius* (branch 8). The latter also contained one of the two annotated jasmonate derivatives. Compounds of phenylpropanoid-, flavonoid- and hydroxycinnamic acid-like structures were predominantly clustered in branches six and seven, whereas branch six contained fragment spectra of *Galium* spp., *P. lanceolata* and *A. pratensis* specific compounds and branch seven fragment spectra of *R. acris* and *P. lanceolata* specific compounds (Fig. 6, Supplementary Fig. 10). Branches three and eight instead contained compounds of either different classes of chemically unrelated or unclassified compounds (Fig. 6, Supplementary Figs. 7 and 12). These branches are heterogeneous in the species origin. Two compounds (931.2829 m/z at 3.67 min, 501.1253 m/z at 4.07 min) were exclusively found in the exudates of *P. lanceolata* roots and might represent irido glycosides (Table 2).

**Table 2:** Putative classification of species-specific semi-polar compounds.

Classes			*A. millefolium* (22)	*G. mollugo* (40)	*G. verum* (5)	*Galium spp.* (37)	*P. lanceolata* (59)	*R. acris* (19)	*A. pratensis* (6)	*A. elatius* (25)	*D. glomerata* (0)	*L. perenne* (1)	*P. pratensis* (1)
Glycosides (19)		Glycoside, acidified (2)						2					
		Glycoside, sulfated/phosphorylated (2)	1				1						
		o. Glycoside1 (14)	5			3	3	1	1				1
Phenylpropanoid (46)		Phenylpropanoid, Coumarin derivative^a^ (1)	1										
	Flavonoids (15)	Flavonoid, glycosylated (3)		1			2						
		Flavonoid, Kaempferol derivative^b^ (3)		2		1							
		o. Flavonoid^a^ (9)		4	1	1	3						
	Hydroxycinnamic acid (40)	Hydroxycinnamic acid amid^a^ (3)					2	1					
		Hydroxycinnamic acid, glycosylated (16)	2	2		2	9	1					
		Hydroxycinnamic acid^a^ (21)	1	4		4	6	2	2	1		1	
Jasmo-nate (2)		Jasmonate derivative, glycosylated (1)					1						
		Jasmonate derivative^a^ (1)								1			
Polyke-tide (5)		Polyketide^a^ (5)		4		1							
Terpene (12)		Terpene / Hydroxycinnamic acid (1)				1							
		Terpene, Iridio glycoside (2)					2						
		Terpene, Sesqueterpene^a^ (8)			2	4				2			
		Terpene, Sesqueterpene, methoxylated^a^ (1)								1			
unclassified (122)		Unclassified, aliphatic acid^a^ (7)								7			
		Unclassified, imin (1)			1								
		Unclassified, imin, aliphatic acid (1)					1						
		Unclassified, methoxylated (2)			1				1				
		Unclassified, sulfate/phosphate residue^a^ (9)	1				8						
		Unclassified, sulfate/phosphate residue, aliphatic acid (1)								1			
		o. Unclassified compounds (101)	11		19	23	22	12	2	12			

^a^Chemical classes contain compounds being classified on the base of one identifier fragment.

^b^The annotation of compounds as a kaempferol derivative bases on identifier fragments of kaempferol and spectral similarity. This has to be confirmed by analytical standards.

The table contains the total number of compounds (in brackets) of each class as well as the occurrences in the samples of the ten different species. Numbers in brackets behind the species represent the total amount of specific compounds per species.

### The exudation of semi-polar metabolite is differentially affected by the environment in case of forbs and grasses

**Figure 7:**
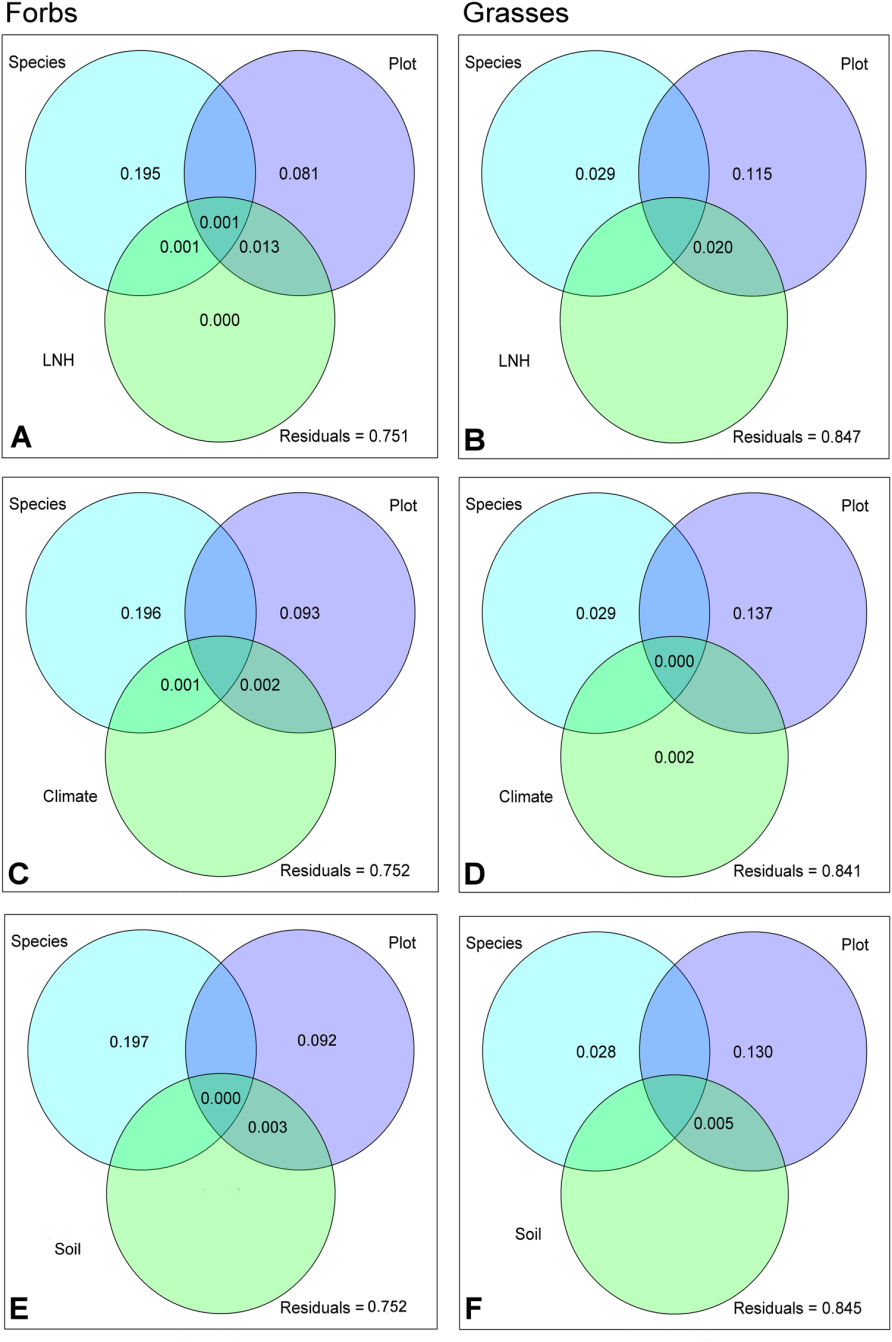
Variance partitioning of semi-polar metabolite composition. Venn diagrams present the proportion of variance in metabolite patterns of forbs (left) and grasses (right) explained by different predictors: Species = species identity of the target plant, Plot = location impact, LNH **(A,B)** = plant local neighbourhood community in a radius of 25 cm around the target plant, climate **(C,D)** = combined characteristics describing the temperature and precipitation, soil **(E,F)** = combined characteristics describing the soil of the location where the target plant was growing.

The results of variance partitioning of the semi-polar metabolites strongly differed between the two growth forms. In sum, the predictors explained less of the variation in semi-polar exudate profiles of grasses than those of forbs (up to 15.9% and up to 24.9% for grasses and forbs, respectively, Fig. 7). For grasses the largest proportion of variance was explained by plot, in forbs most of the variation was accounted by species identity (Fig. 7A,C,E, Supplementary Fig. 2C). The predictors LNH, Climate, Soil and Env did not have any explanatory power, whereas single environmental variables explained the variability in semi-polar metabolite profiles to a minor extent (Supplementary Table 8). The inclusion of LUI as predictor resulted in a minor amount of explained variance (0.34 and 0.96% for forbs and grasses, respectively). This is caused by the effect of fertilization and grazing on the exudation of grasses and forbs (Supplementary Table 8).

The correlation of semi-polar metabolite profiles with single environmental variables revealed a strong environmental impact on the exudation of many secondary metabolites (Supplementary Table 5b,c)). 21.90% and 17.49% of compounds detected in forb and grass exudate samples, respectively, could be linked to one of the environmental variables (Supplementary Fig. 13). Soil variables such as moisture and soil texture but also the climate variable precipitation and T(200) were significantly correlated to semi-polar compounds (Supplementary Fig. 13, Supplementary Table 5b,c)). In general, LUI and LNH variables had a similar effect on the metabolite exudation. In particular, mowing is the variable of LUI with the highest number of affected features (forbs: 89, grasses: 80), whereas Cover was involved in the exudation of 106 features and Shannon in 101 features in forbs and grasses, respectively (Supplementary Fig. 13, Supplementary Table 5b,c)). Interestingly, there are species-specific compounds among these correlated compounds (Supplementary Table 5b)). For instance, LUI traits could be linked to compounds of the phenylpropanoid metabolism and glycosides of various species. Furthermore, compounds of hydroxycinnamic acid-like character exuded by *A. millefolium* (619.1862m/z_3.12 min), *G. mollugo* (381.0621 m/z_7.17 min) *and P. lanceolata* (445.2057 m/z_3.4 min) were significantly correlated to climate variables, whereas flavonoids exuded by *G. mollugo* (507.2066 m/z_4.98 min)*, G. verum* (389.1117 m/z_3.09 min) and *P. lanceolata* (645.1817m/z_4.78 min) or potential terpenes released by the *Galium spp*. (267.0304 m/z_9.64 min) and *A. elatius* (549.3422 m/z_7.18 min) were significantly correlated with soil variables.

## Discussion

Root exudation is a complex process in which a diverse chemical cocktail of substances is released into the rhizosphere. Most studies focus on the investigation of either single substances or specific chemical families^14,20,30,31,38^ and with this they neglect the complexity of exudate profiles. The untargeted metabolite profiling approach presented here allowed not only the detection of 3,185 features but also the classification of 182 substances into various chemical families. Thus, this represents a highly comprehensive exudate analysis of plant species that were not characterized in such a detail, so far. Furthermore, the semi-polar metabolites were designated by chemical classification and grouped according to spectral and fragment similarities^44^. With this, the time consuming bottle necks of tradotional substance identification and categorization, the lack of appropriate analytical standards and the large gap in the knowledge of the majority of metabolites^3,14,40^, in almost all untargeted metabolomics investigations^14,39,40,44^ was overcome. A clustering by fragment similarity and classification of indicative shared fragments could help to overcome this obstacle. Thus, this method provides a basis for the further elucidation of such metabolites and their characterization.

The overall composition of the metabolite profiles of the investigated transplants showed a quite common set of compounds in all ten species, but also differences due to various impacting factors. Moreover, it played a role when the metabolites were categorised into the polar or semi-polar metabolite profile. The chemical composition of polar metabolites was qualitatively similar between the species and less affected by growth form (issue 1 of this study). Semi-polar metabolite compositions, however, showed major differences between forbs and grasses (issue 1 of the current study). Here, forbs had a higher diversity in their profiles. This can be linked to the high impact of species identity and the tendency of forbs to exude more species-specific metabolites than grasses. The high importance of this factor on the exudate composition of forbs could be explained by the phylogenetic distance between the species of both growth forms. It was shown that the genus of a species can impact the diversity in the metabolite profiles^45^. Thus, the larger phylogenetic distance between the forbs compared to the *Poaceae* grasses could affect the result. This assumption is consistent with the results of Herz *et al*.^19^ and Dietz *et al*.^21^. Both studies investigated the impact of endogenous factors on plant root exudation of the same ten target species. In contrast to Herz *et al*.^19^, the present study revealed the factor growth form of being of minor importance for polar metabolites (issue 1 of this study). This might be due to the different exposure times in the field. The transplants of Herz *et al*.^19^ and Dietz *et al*.^21^ grew three month in the field, whereas the plants analysed here were exposed to field conditions for more than one year.

Another explanation for the differences in the role of growth form in the polar metabolite exudation might be the inclusion of further aspects of the experiment, e.g. the site (issue 1 of the current study). The German Biodiversity Exploratories were set up along environmental gradients^42^, in which Schorfheide represents a special site. This was particularly obvious at the level of soil, nutrient cycles and organismic interactions^35,43^. The Schorfheide-Chorin exhibits a higher soil moisture and lower pH as well as higher nitrogen and carbon content than the Swabian Alb and Hainich-Dün^35,43^. Low pH and high soil moisture trigger the exudation of alcohols, amino acids and organic acids^27,28^ to overcome the acidification of the plant cells^43,46^, which is caused by anaerobic soil conditions. Previous studies also showed that the release of nitrogen containing metabolites, such as amino acids contributes to the nitrogen content of the soil^47,48^, which in turn triggers the increased release of carbohydrates, organic acids^33^ and phenylpropanoids^31^. Those mediate the uptake of nutrients by enhancing their absorption or interacting with decomposing organisms^49^. This might explain the occurrence of some of the amino acids, carbohydrates, organic acids and phenylpropanoids in the exudate profiles of the plants investigated here. The inclusion of site might also be the reason for the divergence in the impact of growth form between this study and Herz *et al*.^19^, who omitted this further sub-classification.

The present study revealed that grasses showed a higher chemical richness in exuded polar metabolites in SCH compared to forbs. This implies that grasses exhibit a higher potential for environmental adjustment than forbs^5,8^, which is supported by further results of this study. As already described by Herz *et al*.^19^ and Dietz *et al*.^21^, plot characteristics are also the main drivers for semi-polar metabolite exudation of grasses, but also polar metabolites which were released of both growth forms (issue 1 of this study) in this study. In addition, the present study further resolved the impact of the individual environmental traits to shed more light on their influence (issue 2 the current study). Single variables of soil and climate altered the exudate composition. Soil variables, such as soil moisture and the soil characteristics of the WRB database (soil texture, soil type) had a high relevance for the exudation of polar and semi-polar compounds in both growth forms. The relation of soil moisture and pH to polar and semi-polar compounds like hydroxycinnamic acids or terpenes is remarkable and not described so far. Also the relation of climatic drivers like aboveground temperature to polar and semi-polar compounds needs further investigation.

In previous exudate studies^19,21^ of the ten target species in grasslands, minor impact of neighbouring plants and no impact of land use was found. In contrast, the results of the present study revealed a contribution of single variables of these predictors to the variance in plant metabolite exudation. The impact given by LNH underlines the suggestion that longer residential time could play a role in the exudation of plants in a field plant community^19,21^. Therefore, a better adaptation and a stronger interaction with their locally neighbouring plants, as described for other species^4,22,50,51^, is highly likely. An impact of the plant neighbourhood on a plant after a long residential time was already investigated for belowground root development and plant fitness by Ravenek *et al*.^7^. The impact of the LUI also matches the observations of other studies to the impact of land use on ecosystems^8,36^. The results would support the functions of semi-polar metabolites as mediators of interaction with neighbouring plants^4,22,23^, but also polar and semi-polar metabolites as adaptive agents to abiotic factors^27,28,33,47,48^ such as LUI. So far, the nature of these interactions of exudates and LUI or LNH factors, respectively, is not clear. Thus, further investigation of the correlated exudates with variables of the predictors LUI and LNH might expand the knowledge of plant-plant interaction and land use impact.

It has to be noted that the presented relations between environmental factors and exudates are to the greatest extent not the result of the typical relation of cumulated predictors to the dataset of interest (here the exudates)^19,21,36^ (issue 2 of the current study). The findings discussed here are the result of the impact of single variables on compound composition and single compounds. Quenching effects might be the reason. Those effects might result from either less explanatory power of single variables or the number of compounds that were not correlated with the endogenous and exogenous factors. This might reduce the overall explanation power of the predictors. On the other hand, the higher impact of logistic models compared to variance partitioning is also reasonable. In logistic models, the non-linear character of the analysis can result in a much earlier significant correlation of two variables, here an exuded metabolite and an environmental factor, than by the variance partitioning. However, variance partitioning is affected by the presence of a metabolite in comparison to the overall metabolite profile, whereas logistic models compare each compound individually with the specific environmental variable. Further statistical methods could help to clarify this point.

Moreover, the compounds with a linkage to different factors are of specific interest and this study provides a large set of those compounds. They are not identified so far. Thus, it would be of great interest to reveal their chemical identity by the identification approaches provided by different analytical techniques^31,52,53^.

Although plot together with different individual variables of the neighbouring plants, land use, climate and soil could partially explain the polar and semi-polar exudate profiles of the ten species (up to 31.6% and 24.9%, respectively), an unexplained variance of 68.4% to 68.9% in polar metabolites and of 75.1% to 84.7% in semi-polar metabolites remained. This points to further unrevealed variables influencing the exudation. For instance, single aboveground events (trampling and erosion) or further root surrounding organisms like bacteria, fungi and herbivores might explain the appearance of certain exuded metabolites and the exudate composition. This is especially of huge interest for some polar metabolites, as amino acids, organic acids and carbohydrates, known to be released as response to the microbial community surrounding the root^20,54^. But also several semi-polar metabolites are described as interaction mediators between plants and as defence agents against bacteria or fungi^17,55–57^. They also act as inhibitors of the growth of plants^58^ and as toxins for herbivores^18,59,60^. Also endogenous factors e.g. the plant functional traits^19–21^ and plant age^20^ can alter the exudate composition in the rhizosphere. Herz *et al*. (2018)^19^ and Dietz *et al*. (2019)^21^ demonstrated for the same ten plant species that in both cases, polar and semi-polar exuded metabolites, plant functional traits such as root biomass and C content of the roots have an impact on the exudate profile of plant roots. Such investigations were not possible in the current study due to detection limitations of different plant functional traits of the ten phytometer species (data not shown), however, they should be part of future investigations. Aulakh *et al*. (2001)^21^ presented qualitative and quantitative changes in the metabolite profile of organic acids of rice cultivars in dependence of the plant developmental stage. The experimental design of the current study did not allow such relations, which might also play a role in the grassland rhizosphere. In conclusion, this study demonstrates the diversity of exudate profiles of polar and semi-polar metabolites of different forb and grass species in the field. Only the combined investigation of a broad set of metabolites and different ecosystem components can help to find the most probable explanation why plants release a part of their metabolome into the soil and thereby might provide information about the potential biological function of exudates in the rhizosphere.

## Methods

### Experimental setup

The experiment was performed during May 2014 and August 2015 in the three regions of the German Biodiversity Exploratories^42^: Schorfheide-Chorin, Hainich-Dün and Swabian Alb. The sites differ in their location, climatic and soil properties^42,43,46,61,62^.

18 experimental grassland plots in each Exploratory (54 in total) varying in land use intensity were selected for the analyses. In total, 10 species were raised for the experiment: five forbs (*Achillea millefolium* L. [Asteraceae], *Galium mollugo* L., *Galium verum* L. [Rubiaceae], *Plantago lanceolata* L. [Plantaginaceae], *Ranunculus acris* L. [Ranunculaceae]) and five grasses (*Alopecurus pratensis* L., *Arrhenatherum elatius* [L.] P.Beauv. ex J.Presl & C.Presl., *Dactylis glomerata* L., *Lolium perenne* L., *Poa pratensis* L. [all Poaceae]). The cultivation and planting was performed in 2014 with transplants of the same age as described in Herz *et al*.^8^. Supplementary Table 9 presents the number of samples per site and species.

### Environmental factors and data collection

Climate data of the precipitation (in %) and temperature in 10 cm and 2 m height (T(10) and T(200), in °C) as well as soil moisture (moisture, in %) were provided by Biodiversity Local Management teams^42^. Soil pH (pH), the total carbon (TC, in %) and total nitrogen content (TN, in %) of the soil of each site were measured on soil samples of bulk soil of each target plant. The soil was collected, sieved (2 mm mesh size), dried at 105 °C and ground. pH was determined by mixing 10 g soil powder and 25 ml demineralized water. 1.86 g KCl was added and pH was measured by a glass electrode. TC and TN were determined by weighing 10 mg soil powder into tin capsules and analysed using a C/N-analyser (vario EL cube; Elementar).

Agricultural management was investigated by calculating the Land use intensity index of 2015 (LUI) according the following formula of Blüthgen *et al*.^36^:

$$\begin{array}{c}{\rm{LUI}}=\frac{{\rm{Fi}}}{{\rm{Fr}}}+\frac{{\rm{Mi}}}{{\rm{Mr}}}+\frac{{\rm{Gi}}}{{\rm{Gr}}}{\rm{i}}={\rm{factor}}\,{\rm{per}}\,{\rm{plot}}\\ \,\,\,\,\,\,\,\,\,{\rm{r}}={\rm{mean}}\,{\rm{factor}}\,{\rm{within}}\,{\rm{the}}\,{\rm{site}}\end{array}$$

The factors account for the amount of N fertilizer in kg per ha for the growth time of the target plants (fertilization, F), the annual mowing rate in (mowing, M) and annual grazing frequency in livestock units days of grazing per ha per year (grazing, G). All abbreviations are summarized in Supplementary Table 10.

### Exudate sample collection

The sample collection took place from June to August 2015. A field exudate collection method was adapted to those of Herz *et al*.^19^ and Dietz *et al*.^21^ to collect the polar and semi-polar metabolites. A wash step of the roots in 0.5% sodium chloride solution (NaCl) for 10 min was inserted between wash step one and two to remove rhizosphere microorganisms form the root surface. The exudate collection was performed in deionised water of HPLC quality from the complete root. Water samples without root exudation were used as process control (“water blanks”).

An internal standard stock solution containing 20 µg/mL 2,4-dichlorophenoxyacetic acid and 10 µM Ribitol were added immediately after exudate collection in the field.

The exudate solution was purified by using the approach described in Herz *et al*.^19^ and Dietz *et al*.^21^ and measured with two different non-targeted plant metabolite profiling approaches. Aliquots of 100 µL of each sample were analysed by LC-MS according to Dietz *et al*.^21^. Aliquots of 200 µL of each sample were derivatized as described in Herz *et al*.^19^ and subjected to GC-MS analysis.

### GC-MS analysis and data processing

Derivatized exudates and water controls were analysed by non-targeted plant metabolite profiling with a gas chromatograph (6890N GC; Agilent Technologies, Santa Clara, USA) equipped with a ZB-5 Zebron Guardian^TM^ Capillary GC column (30 m + 10 m Zebron^TM^, iD 0.25 mm, df 0.25 µm; Phenomenex, Torrance, USA) and coupled to mass spectrometer (5975 MSD; Agilent Technologies). Settings and method of measurement as well as data processing were applied as described in Herz *et al*.^19^.

### LC-MS and MS/MS analysis and data processing

Exudate samples as well as water controls were analysed by ultra performance liquid chromatography coupled to electron spray ionisation quadrupole time of flight mass spectrometry (UPLC/ESI-Q-ToF-MS). An ultra performance liquid chromatography (ACQUITY UPLC; Waters, Eschborn, Germany) equipped with an Acquity UPLC® HSS T3 column (ACQUITY UPLC HSS T3 Column, 100 Å, 1.8 µm, 1 mm × 100 mm; Waters) coupled to MicrOTOF–Q II hybrid quadrupole time-of-flight mass spectrometer equipped with an Apollo II electrospray ion source (Bruker Daltonics) was used for MS mode. To obtain CID mass spectra (MS/MS) of exuded compounds UPLC/ESI-Q-ToF-MS with an ultra performance Acquity UPLC platform (ACQUITY UPLC; Waters) equipped with an Aquity UPLC® H5S T3 column (ACQUITY UPLC HSS T3 Column, 100 Å, 1.8 µm, 3 mm × 100 mm, 1/pkg; Waters) and a MicrOTOF–Q I hybrid quadrupole time-of-flight mass spectrometer equipped with an Apollo II electrospray ion source (Bruker Daltonics). Detailed description were provided in the publication of Dietz *et al*.^21^.

### Compound classification and identification

Data were processed as described in Herz *et al*.^19^ and Dietz *et al*.^21^. The identification of GC-MS measured compounds based on the National Institute of Standards and Technology (NIST) data base, the Golm metabolome database (GMD) and analytical standards measured on the same instrument. The classification of LC-MS measured compounds based on comparison of qualifier ions of each MS/MS of each compound with a fragment library of measured reference standards (see also Dietz *et al*.^21^). The specific identifier ions are given in Supplementary Table 7. The hierarchical clustering of mass spectra was done according to their fragment spectra similarity by the MetFamily tool^44^. The coloration of the endpoints was done manually in accordance to family classes and fragments.

### Statistical analysis

The statistical analysis was adjusted to Dietz *et al*.^21^ and was performed either with Excel 2010 or with R (version 3.4.4^63^). First, metabolites and compounds occurring in 50% of water controls as well as in 50% of chemical blanks (GC-MS analysis) were regarded as artefacts and excluded from the metabolite dataset. For investigation of the overall metabolite profile, data were transformed to a presence/absence matrix. This allowed the observation of metabolite composition without the heterogeneity already described in former investigations^19,21^. The chemical richness as well as the significance of differences were calculated by the mean number of measured metabolites per group, here site and species or site and plant, using ANOVA (function aov,^63^ and Scheffé posthoc test (function sheffe, package agricolae^64^). The dependency on traits as species, site and growth form were calculated by linear mixed effect models (function lmer, package lmerTest^65^) including site with plot nested in species or species and plot nested in growth form as random factors. Results were visualized using boxplots (function qplot, package ggplot2^66^).

The exudate composition was analysed by redundancy analysis (function rda, package vegan^67^) of the presence/absence matrix of metabolite composition against a presence/absence matrix of species and site together. GC-MS measured exudates were also analysed for their quantitative occurrence in growth form, site, and growth form and site as well as species, and species and site. First, the counts of each substance over all samples of the group members were summed up and divided by the number of samples per group. This percentage of occurrence of one group member, e.g. ALB, was divided by the sum of percentage of occurrence of all other group members, e.g. SCH and HAI. Metabolites with a ratio of at least 2 were accepted as linked to this group member. LC-MS measured exudates were analysed for the significant species-specific compounds by calculating the mean of the compound composition of each species and subjecting it to a t-test (function binom.test^63^) with alternative hypothesis that a compound does not occur in one out of ten species. In a second test, *Galium mollugo* and *Galium verum* data were combined as *Galium* species due to their phylogenetic origin and compound composition similarity and investigated with the alternative hypothesis that a compound occurs at least in two species.

Polar and semi-polar metabolite matrices were subjected to variance partitioning (function varpart, package vegan^67^) for the calculation of explained variance by target species identity (Species), the plot as local impact (Plot) and either parameters of the neighbour plants around each target plant (LNH) and the environmental conditions (either as cumulated predictors of soil and climate parameters (Env), or soil conditions (Soil), and climate conditions (Climate) separately). The single variables contained in LNH (Cover, Richness and Shannon), LUI (mowing, grazing, fertilization), Soil (pH, soil moisture, soil texture, soil type, TC, TN) and Climate (precipitation, T(10) and T(200)) were investigated for their explanatory power of exudate composition in the same way as the cumulated predictors. Furthermore, logistic regression models (glmer, lme4 package^68^) were performed with the particular metabolites as dependent variable and a single environmental variable as predictor, using plot and species as random factors. Correlations with an alpha error below 0.05 in ANOVA and correlation analysis were considered as significant. The correlated compounds were summed up for each variable in a bar plot (Excel 2010) and divided by the total number of compounds found in the dataset of forb or grass exudates, respectively, for calculation of the percentage of affected compounds per cumulated predictor.

## Supplementary information

Supplementary information

## Data Availability

The mass spectrometric data are available from MetaboLights database LC-MS permanent link: https://www.ebi.ac.uk/metabolights/mtbls865 GC-MS permanent link: https://www.ebi.ac.uk/metabolights/mtbls866^1^ and www.bexis.uni-jena.de. Information concerning environmental data, land use intensity data, and plant community data that support the findings of this study are available from www.bexis.uni-jena.de.
