# Supplementary material for: Root exudate composition of grass and forb species in natural grasslands
Source: Sci Rep. 2020 Jul 1;10:10691. doi: 10.1038/s41598-019-54309-5 (PMC7329890; doi:10.1038/s41598-019-54309-5)
Supplement: Supplementary file 1 — Supplementary information [file 41598_2019_54309_MOESM1_ESM.pdf]

Title: „Root exudate composition of grass and forb species in natural grasslands"

Author list: Sophie Dietz[1*], Katharina Herz[2], Karin Gorzolka[1], Ute Jandt[2,3], Helge Bruelheide[2,3], Dierk Scheel[1, 3]

1: Leibniz Institute of Plant Biochemistry, Weinberg 3, 06120 Halle (Saale), Germany

2: Martin Luther University Halle-Wittenberg, Institute of Biology / Geobotany and Botanical Garden, Am Kirchtor 1, 06108 Halle [Saale], Germany

3: German Centre for Integrative Biodiversity Research (iDiv) Halle-Jena-Leipzig, Deutscher Platz 5e, 04103 Leipzig, Germany

Supplementary Data

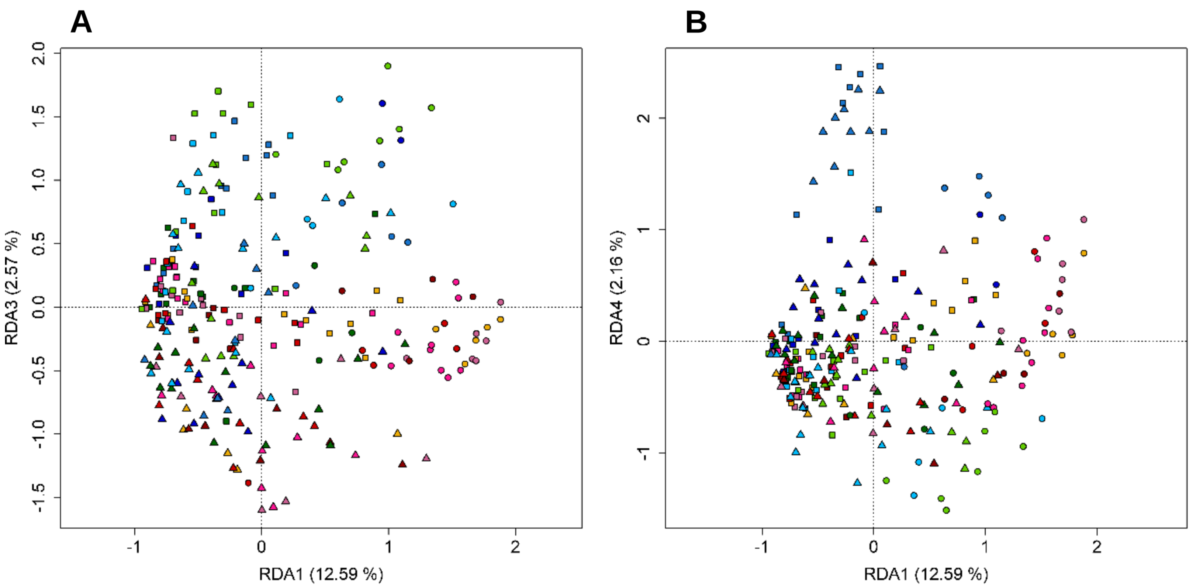

Fig. is continued on the following page

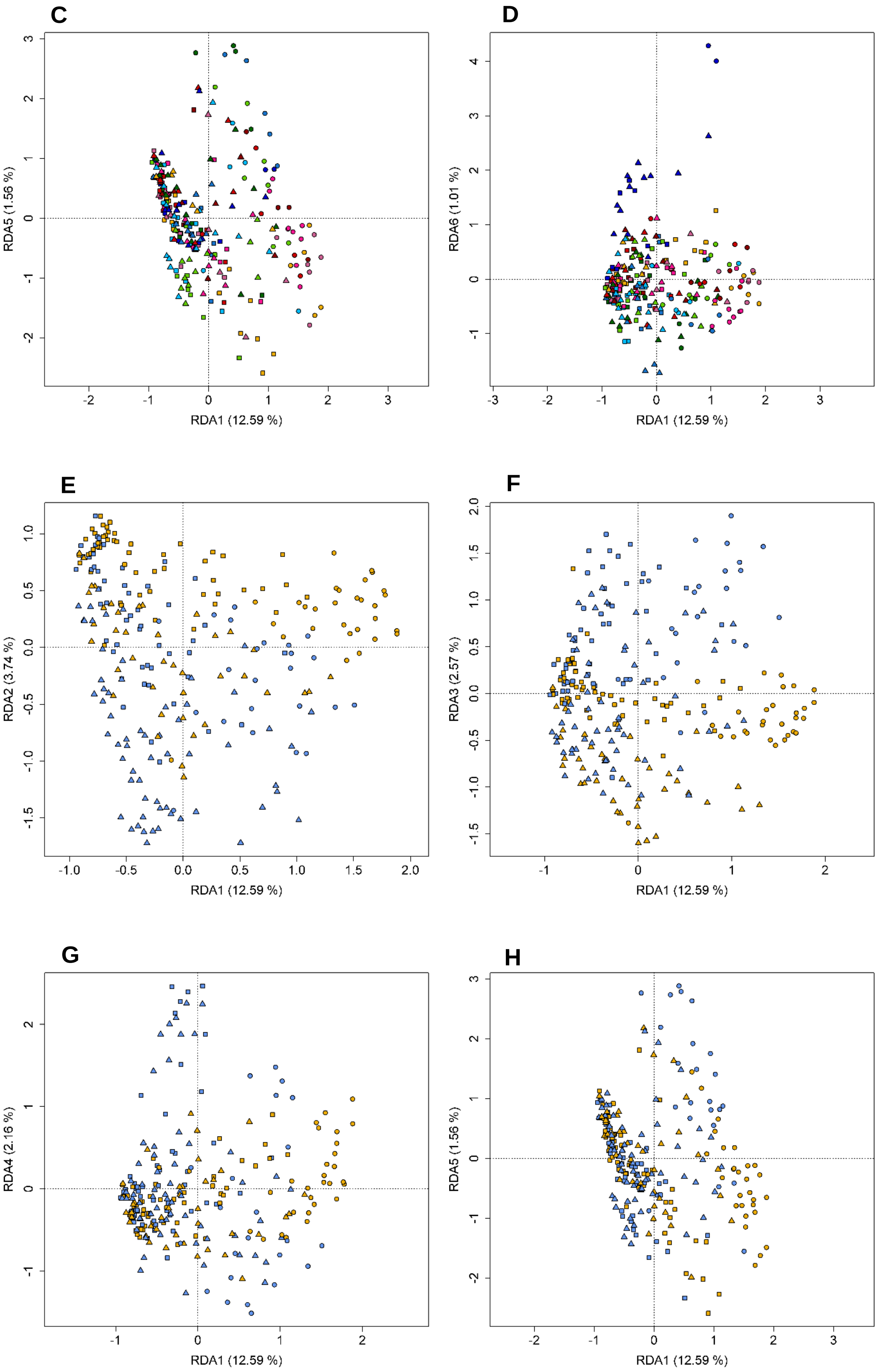
C
RDA5 (1.56 %)
RDA1 (12.59 %)
D
RDA6 (1.01 %)
RDA1 (12.59 %)
E
RDA2 (3.74 %)
RDA1 (12.59 %)
F
RDA3 (2.57 %)
RDA1 (12.59 %)
G
RDA4 (2.16 %)
RDA1 (12.59 %)
H
RDA5 (1.56 %)
RDA1 (12.59 %)

I

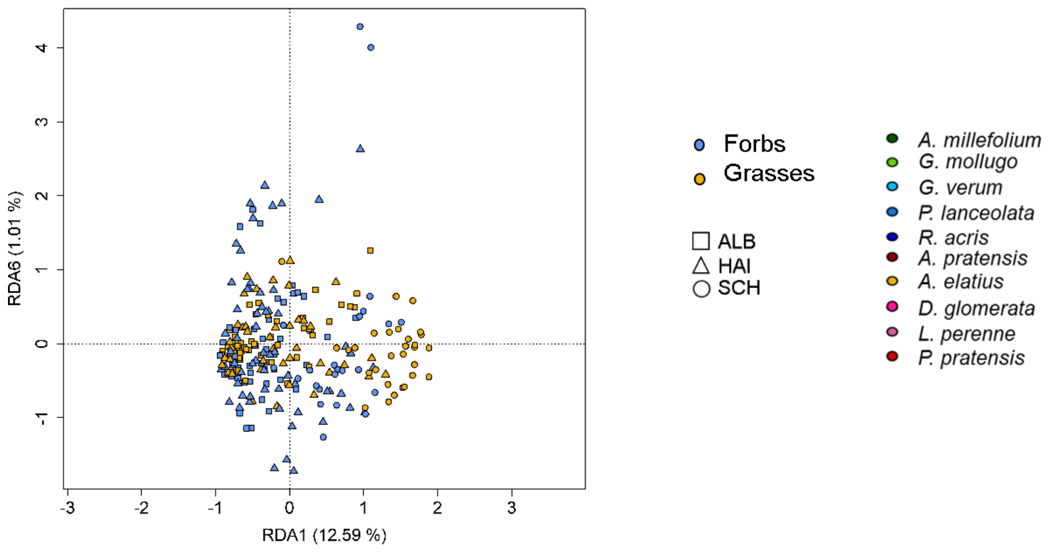

Supplementary Fig. 1: Redundancy analysis of polar metabolites. RDA was performed with 257 samples matching with impacting environmental factor information. Metabolite compositions of the samples were plotted against a presence/absence matrix of species per site. The plots **A-D** represents the RDA axis three to six plotted against rda axis one and were coloured by species (see legend), whereas plots **E-I** represents the RDA axis one against two to six and were coloured by growth form (see legend). Symbols represent the three Exploratories (see legend).

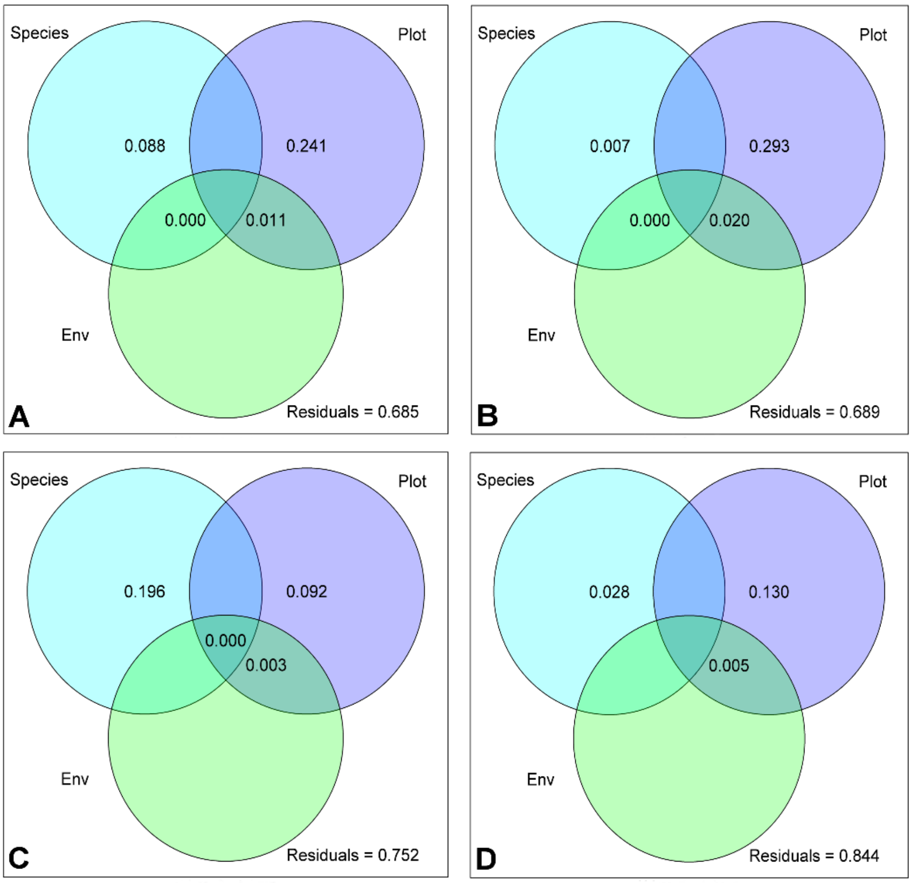

Supplementary Fig. 2: Variance partitioning of polar and semi-polar metabolites. Venn diagrams present the proportion of variance in **A, B** polar or **C, D** semi-polar metabolite pattern, respectively, of forbs (left) and grasses (right) explained by different predictors: Species = species identity of the target plant, Plot = local impact, Env= Environmental properties summarizing climate and soil variables.

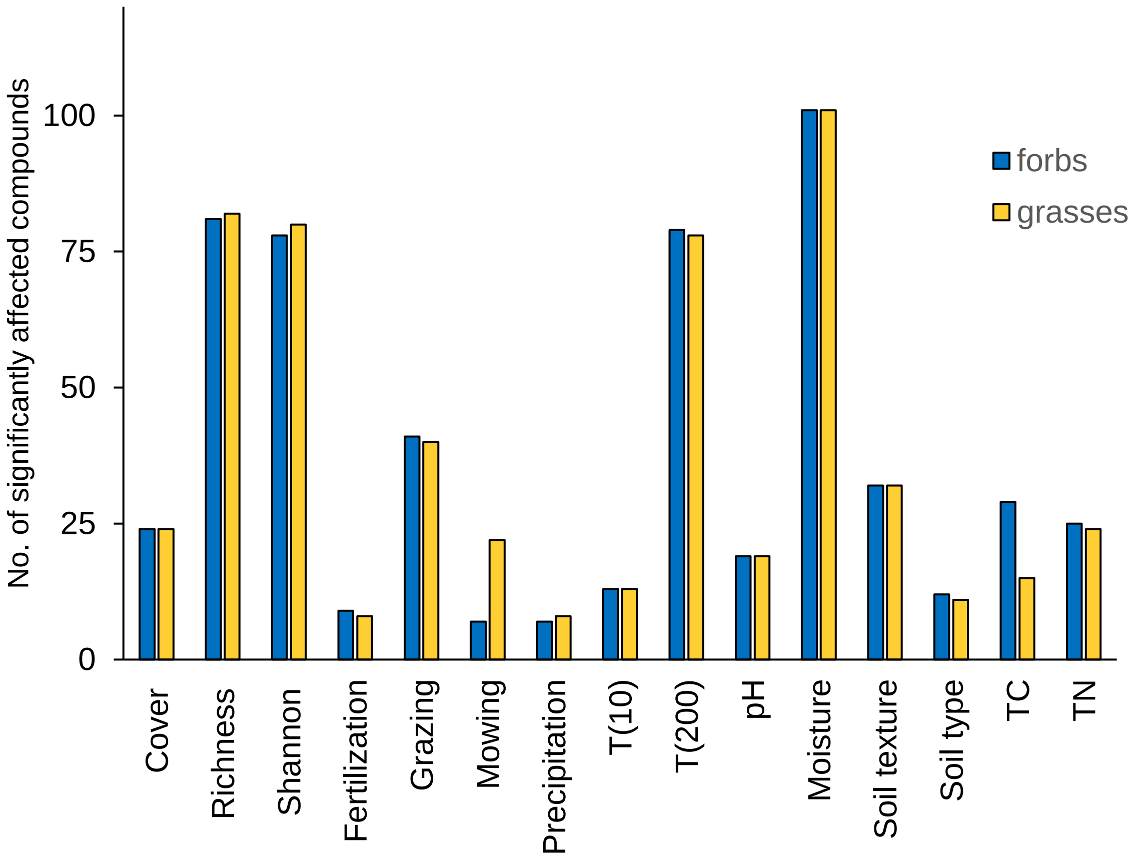

Supplementary Fig. 3: Environmental characteristics affecting polar compounds. Bar plot presents the number of significantly affected polar compounds correlated to the different single variables of the environmental factors LNH, LUI, Climate and Soil. The numbers were calculated for each growth form (see legend). A detailed description of the abbreviation is given in Supplementary Table 10.

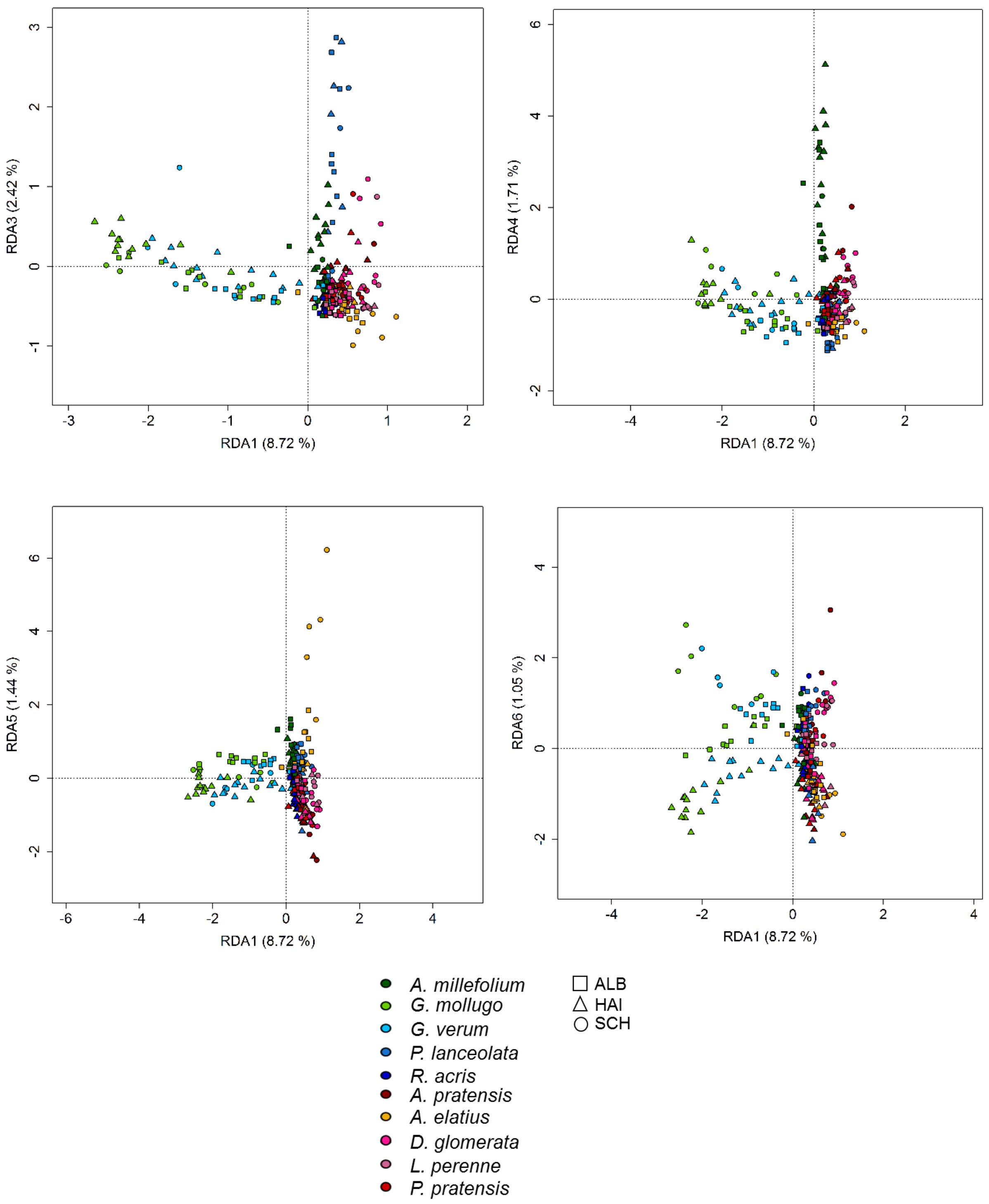

Supplementary Fig. 4: Redundancy analysis of semi-polar metabolites. RDA was performed with 257 samples matching with impacting environmental factor information. Metabolite compositions of the samples were plotted against a presence/absence matrix of species per site. The ten species are represented by colour, whereas the symbols indicate the sites (see legend). Axis three to six were plotted against axis one.

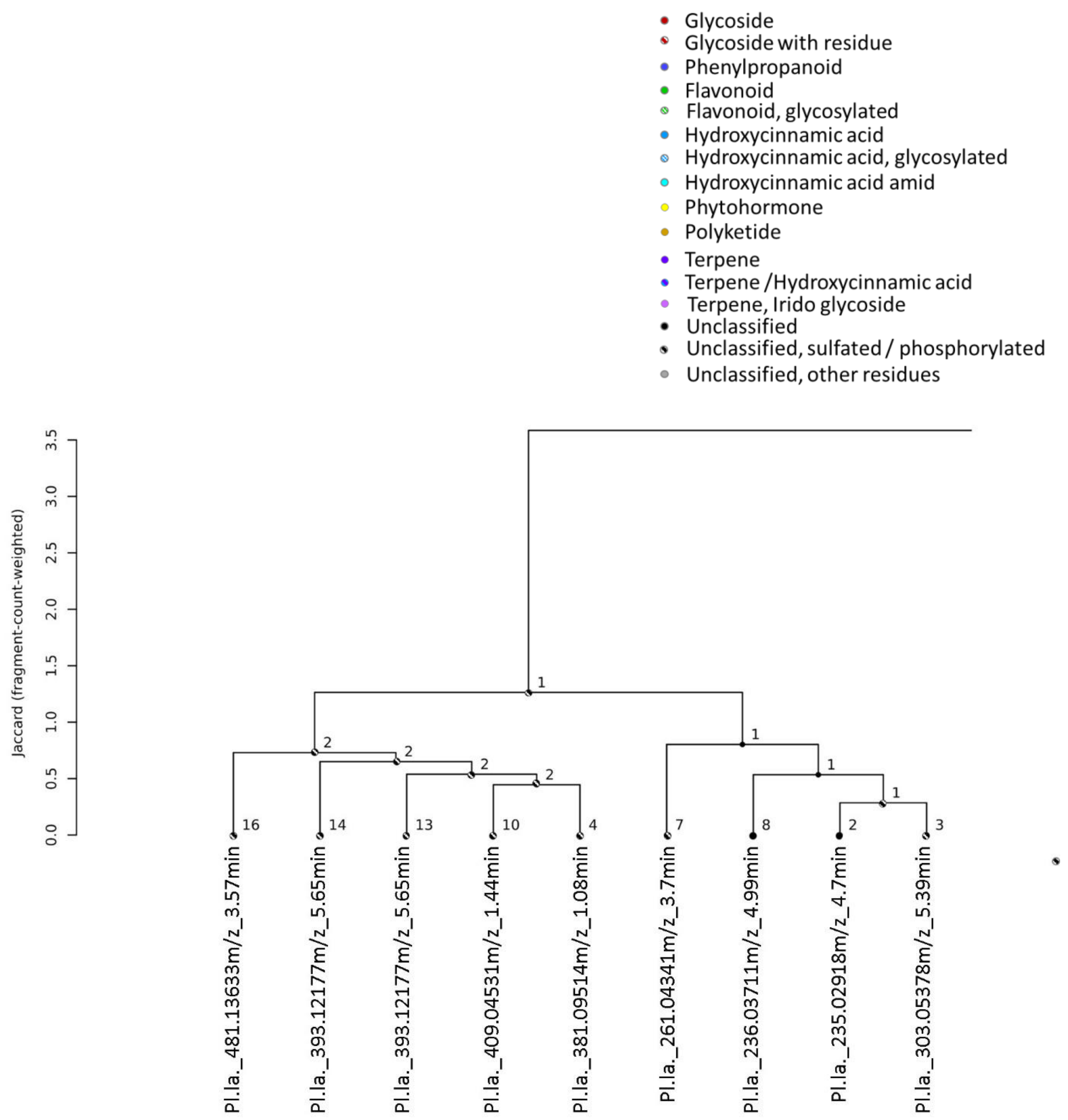

Supplementary Fig. 5: Detailed view of branch 1 of hierarchical clustering of species specific semi-polar exudates. Hierarchical clustering was performed on the tandem-mass spectra of the significant species-specific compounds. Cluster were calculated on spectral similarity rested on Jaccard dissimilarity and fragment-count-weighted value rating. The classification of metabolites is given in the legend.

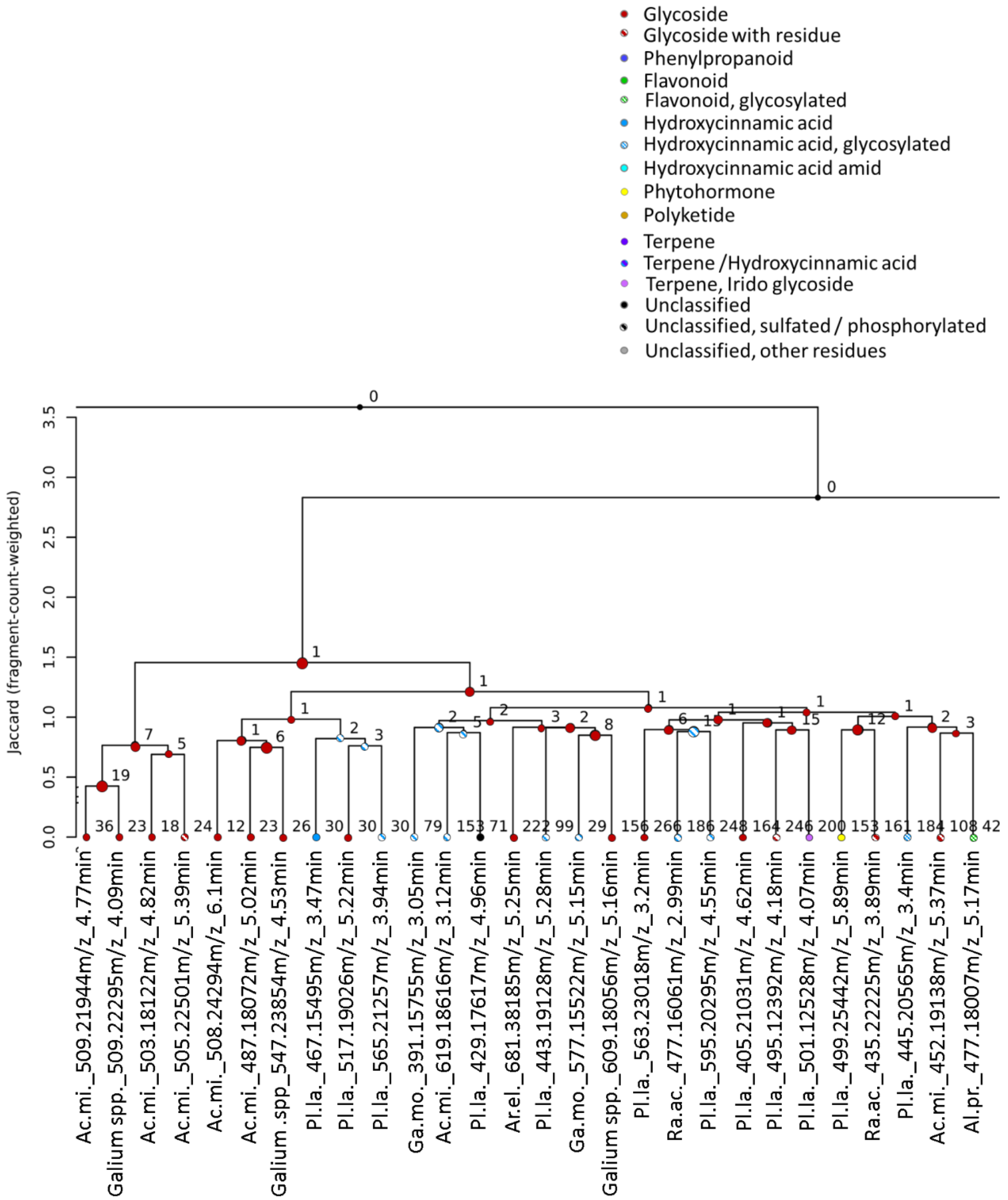

Supplementary Fig. 6: Detailed view of branch 2 of hierarchical clustering of species specific semi-polar exudates. Hierarchical clustering was performed on the tandem-mass spectra of the significant species-specific compounds. Cluster were calculated on spectral similarity rested on Jaccard dissimilarity and fragment-count-weighted value rating. The classification of metabolites is given in the legend.

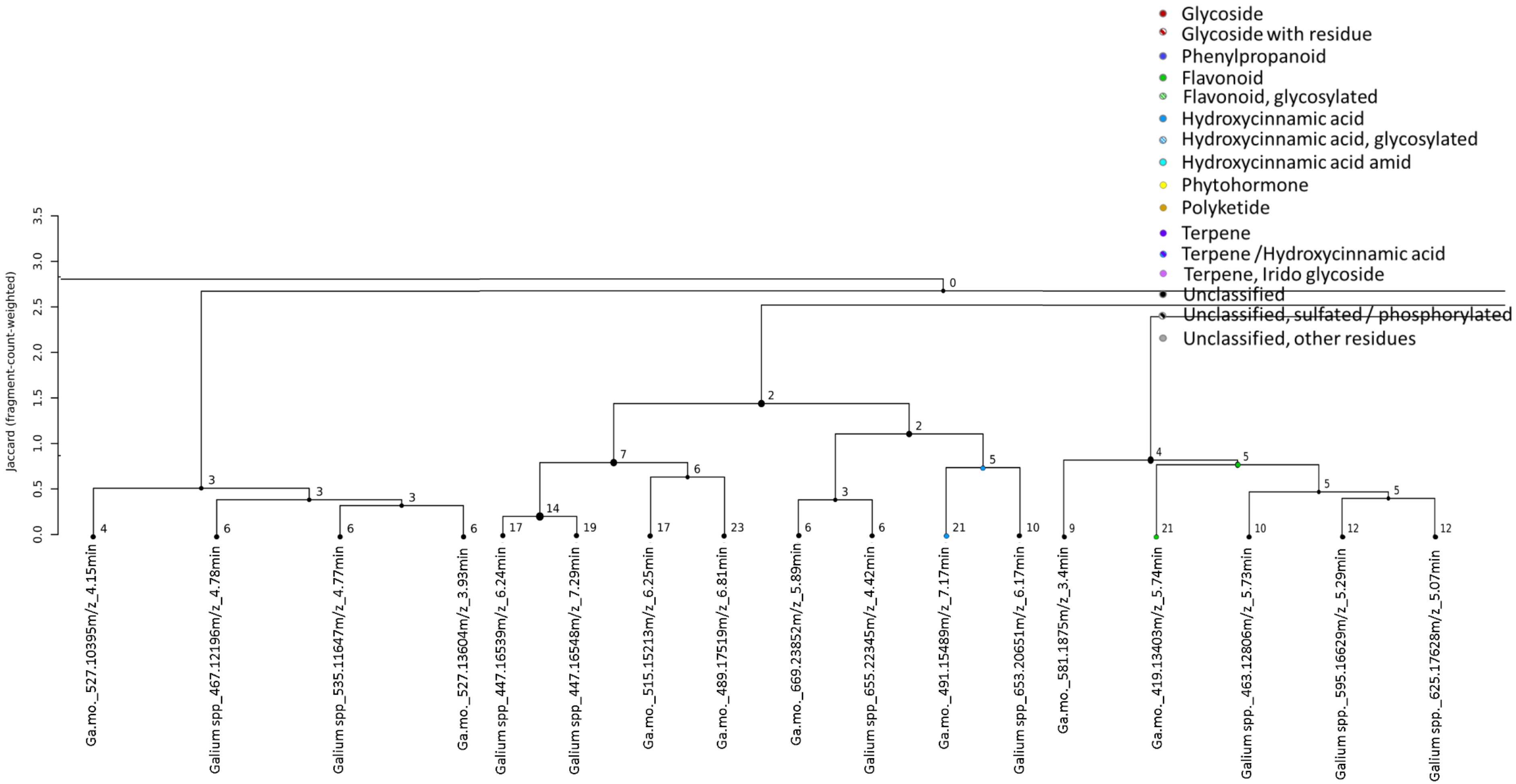

Supplementary Fig. 7: Detailed view of branch 3 of hierarchical clustering of species specific semi-polar exudates. Hierarchical clustering was performed on the tandem-mass spectra of the significant species-specific compounds. Cluster were calculated on spectral similarity rested on Jaccard dissimilarity and fragment-count-weighted value rating. The classification of metabolites is given in the legend.

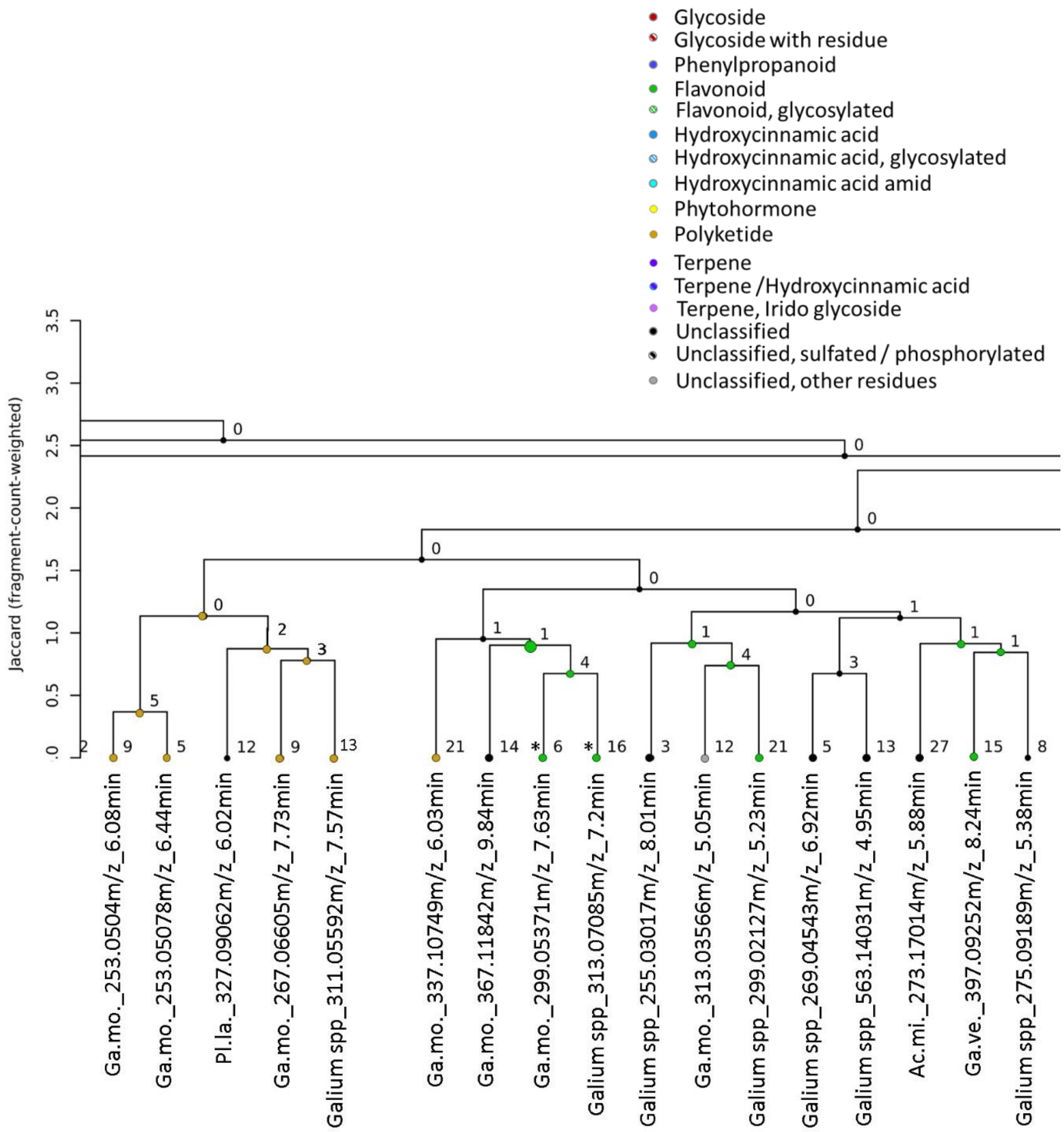

Supplementary Fig. 8: Detailed view of branch 4 of hierarchical clustering of species specific semi-polar exudates. Hierarchical clustering was performed on the tandem-mass spectra of the significant species-specific compounds. Cluster were calculated on spectral similarity rested on Jaccard dissimilarity and fragment-count-weighted value rating. The classification of metabolites is given in the legend.

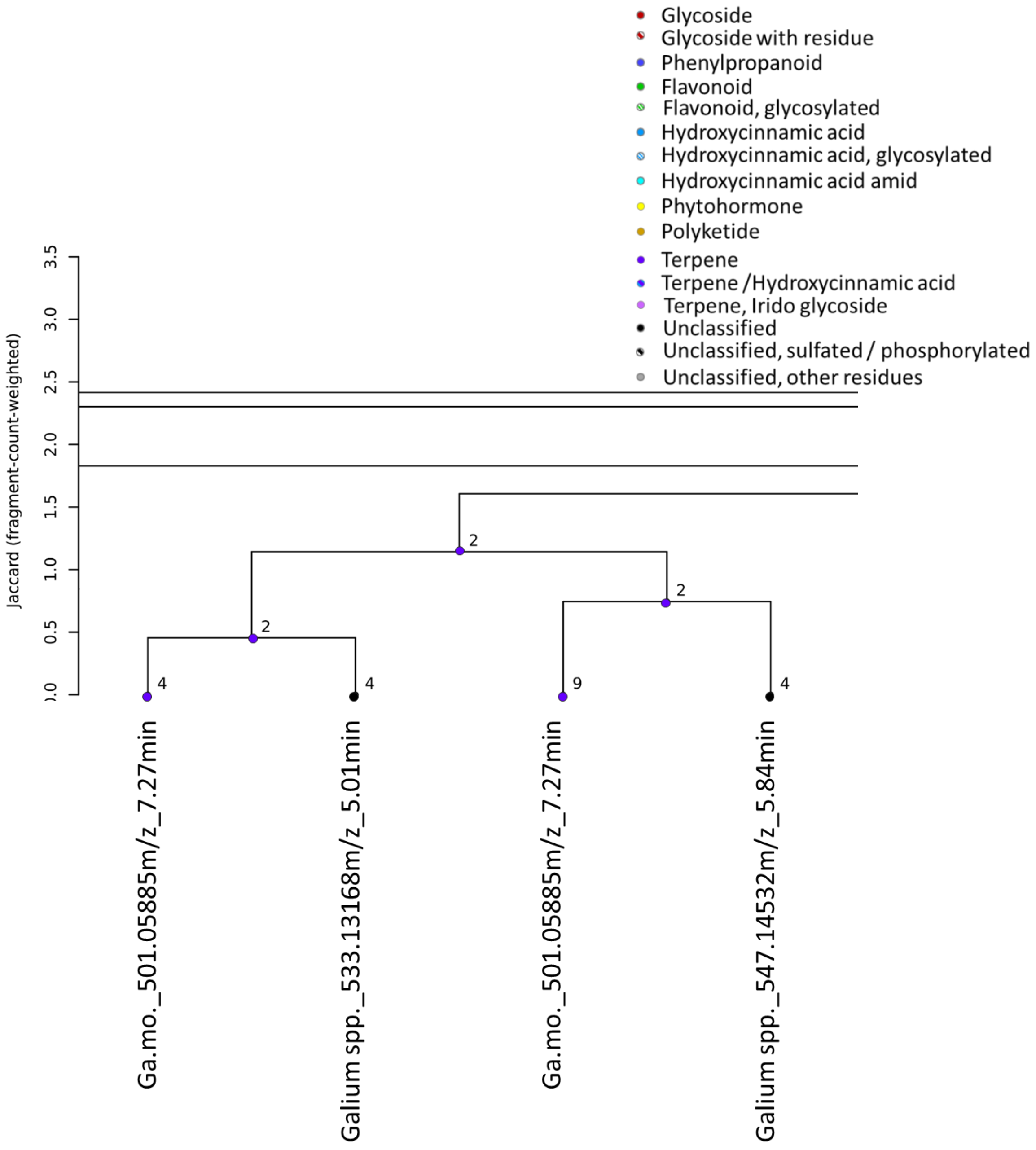

Supplementary Fig. 9: Detailed view of branch 5 of hierarchical clustering of species specific semi-polar exudates. Hierarchical clustering was performed on the tandem-mass spectra of the significant species-specific compounds. Cluster were calculated on spectral similarity rested on Jaccard dissimilarity and fragment-count-weighted value rating. The classification of metabolites is given in the legend.

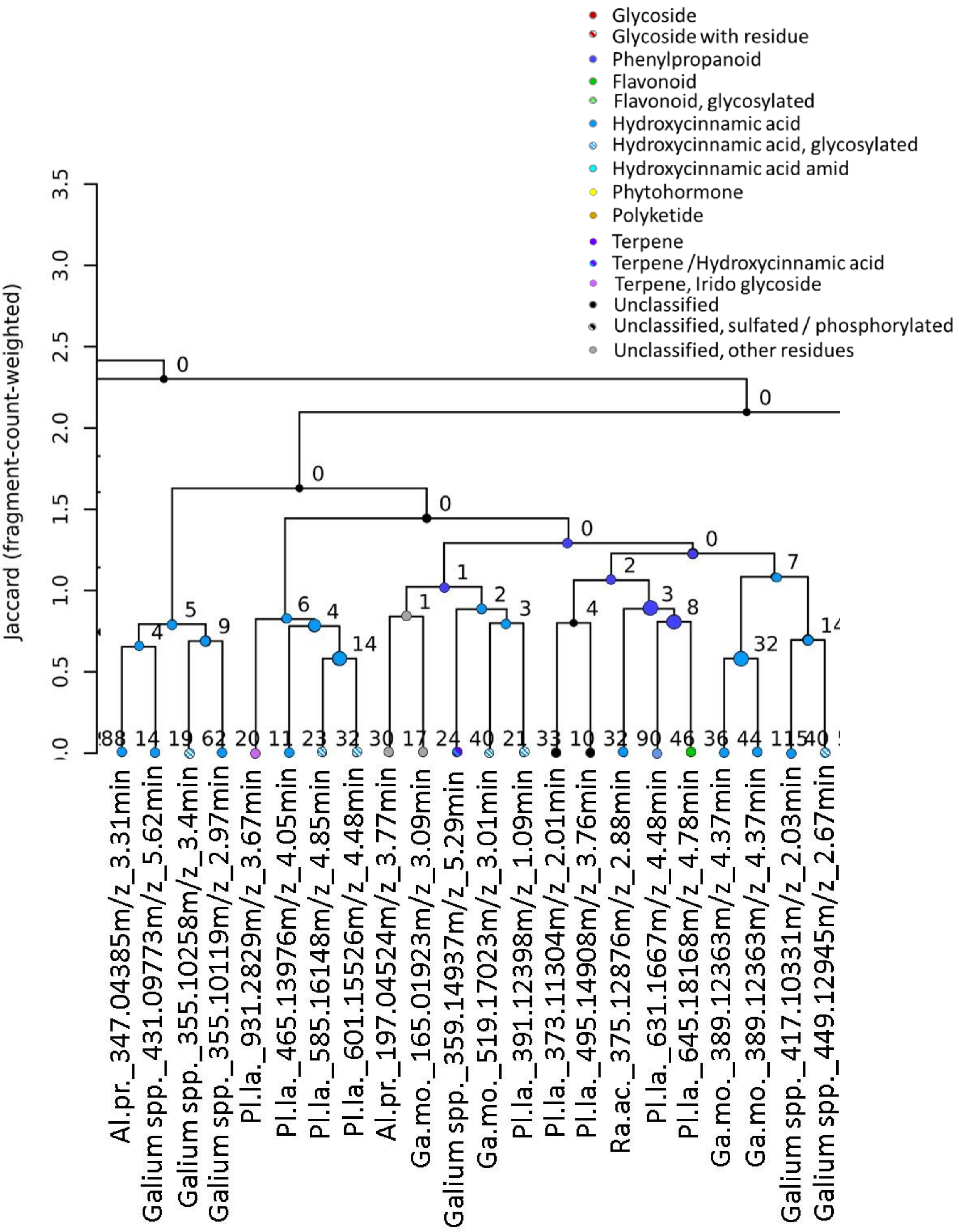

Supplementary Fig. 10: Detailed view of branch 6 and 7 of hierarchical clustering of species specific semi-polar exudates. Hierarchical clustering was performed on the tandem-mass spectra of the significant species-specific compounds. Cluster were calculated on spectral similarity rested on Jaccard dissimilarity and fragment-count-weighted value rating. The classification of metabolites is given in the legend.

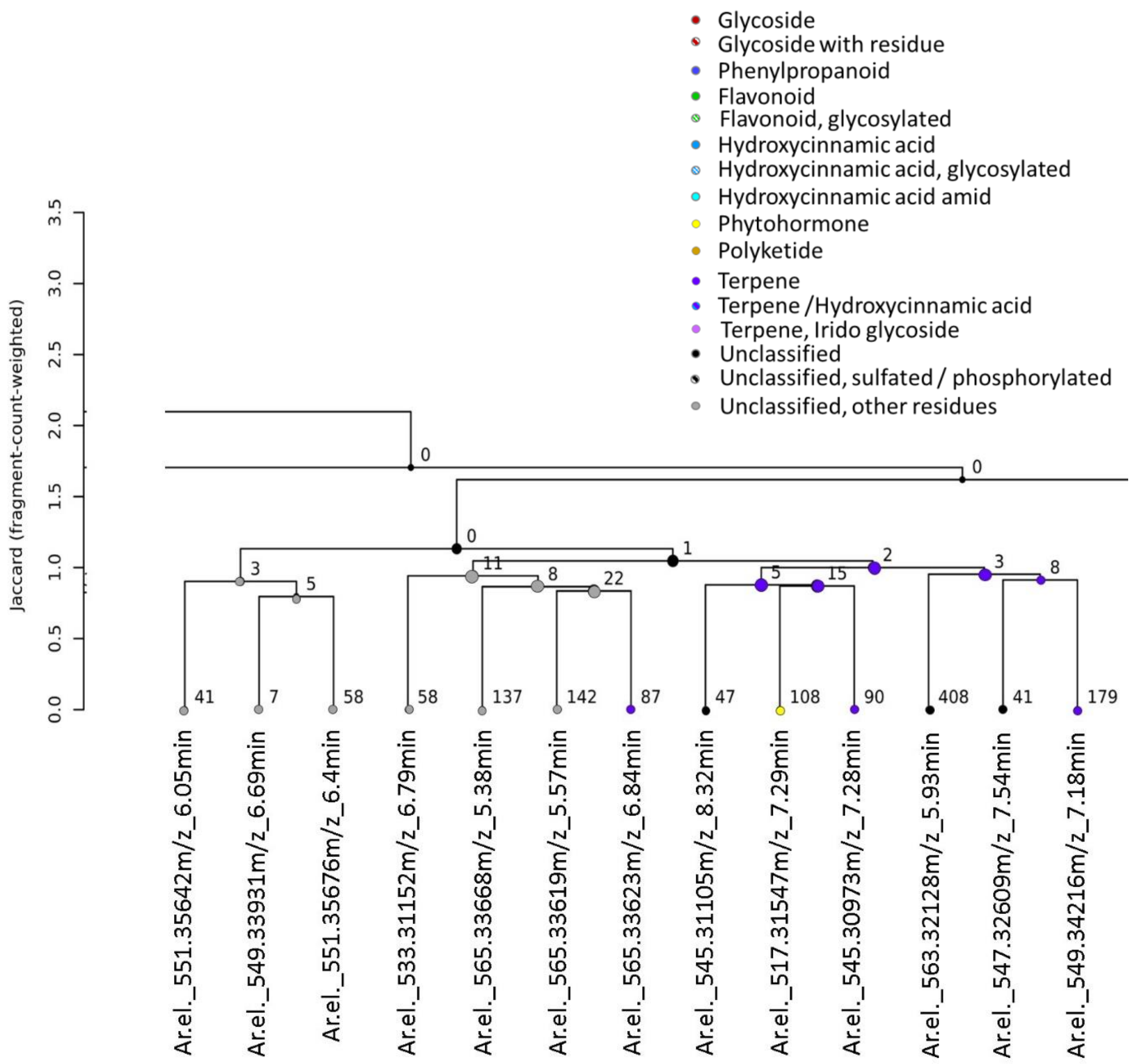

Supplementary Fig. 11: Detailed view of branch 8 of hierarchical clustering of species specific semi-polar exudates. Hierarchical clustering was performed on the tandem-mass spectra of the significant species-specific compounds. Cluster were calculated on spectral similarity rested on Jaccard dissimilarity and fragment-count-weighted value rating. The classification of metabolites is given in the legend.

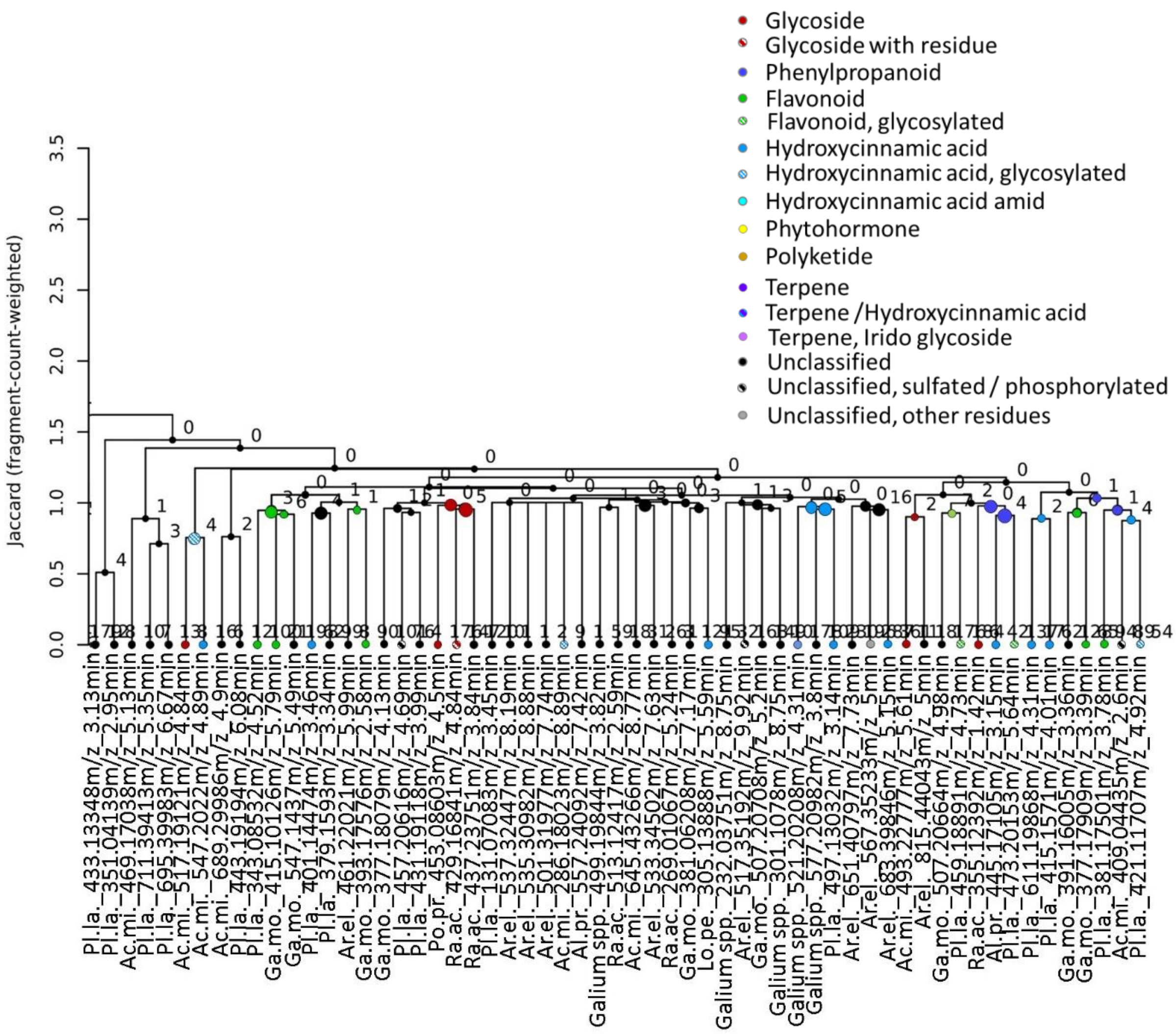

Supplementary Fig. 12: Detailed view of branch 9 of hierarchical clustering of species specific semi-polar exudates. Hierarchical clustering was performed on the tandem-mass spectra of the significant species-specific compounds. Cluster were calculated on spectral similarity rested on Jaccard dissimilarity and fragment-count-weighted value rating. The classification of metabolites is given in the legend.

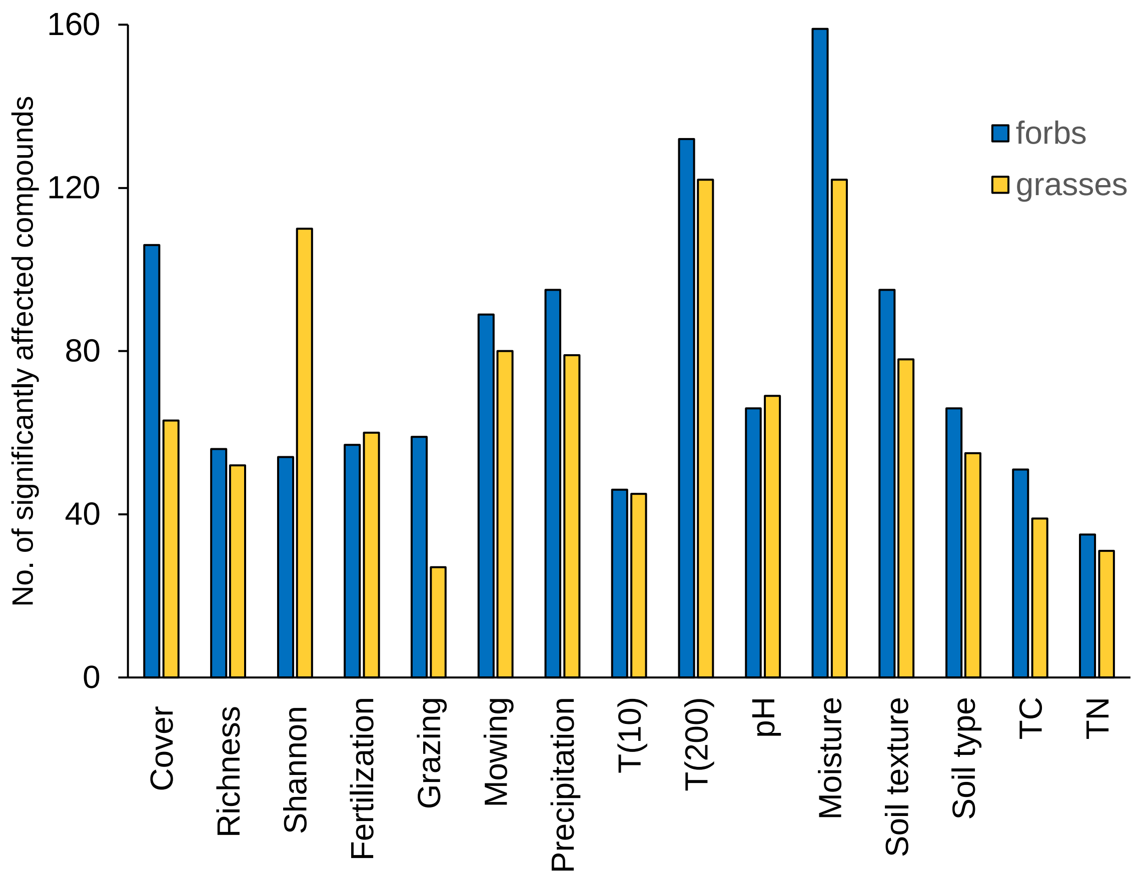

Supplementary Fig. 13: Environmental characteristics affecting semi-polar compounds. Bar plot presents the number of significantly affected semi-polar compounds correlated to the different single variables of the environmental factors LNH, LUI, Climate and Soil. The numbers were calculated for each growth form (see legend). A detailed description of the abbreviation is given in Supplementary Table 10.

Supplementary Table 1: List of all polar metabolites. All annotated and identified polar metabolites are given with their quantifier ion (m/z), retention indices (R_i) and retention time (RT).

Compound Name	class	Quanti-fier ion	Ri	RT [min]
Noradrenalin (174)	alkaloid	174	1759.7	19.38
myo-Inositol (305)	alcohol	305	2133.3	24.23
Pinitol (260)	alcohol	260	1869.4	20.45
scyllo-inositol (204)	alcohol	204	2060	23.33
Sorbitol (217)	alcohol	217	1315.4	11.48
Threitol (217)	alcohol	217	1525.4	15.33
Xylitol (307)	alcohol	307	1735.6	18.59
Benzaldehyde (257)	aldehyde	257	1664.4	17.29
N-Acetylglucosamine (156)	amine	156	1796.5	19.62
Tyramine (174)	amine	174	1926.5	21.36
Alanine (116)	amino acid	116	1113.1	8.01
Asparagine (231)	amino acid	231	1697	18.07
Aspartate (232)	amino acid	232	1540.6	15.32
beta-Alanine (248)	amino acid	248	1436.7	13.63
Glutamate (246)	amino acid	246	1643.3	16.97
Glutamine (155)	amino acid	155	1484	19.36
Homoserine (218)	amino acid	218	1464.4	14.02
Isoleucine (158)	amino acid	158	1302.3	11.28
Leucine (158)	amino acid	158	1279.8	10.98
Lysine (156)	amino acid	156	1942.9	21.55
Methionine (176)	amino acid	176	1533.4	15.21
Ornithine / Citrullin (142)	amino acid	142	1843.3	20.05
Phenylalanine (192)	amino acid	192	1650.6	17.12
Proline (142)	amino acid	142	1304.4	11.32
Serine (204)	amino acid	204	1373.2	12.52
Threonine (218)	amino acid	218	1401.7	13.01
Tryptophan (202)	amino acid	202	2244.5	25.66
Tyrosine (218)	amino acid	218	1961.1	21.89
Valine (144)	amino acid	144	1222.4	9.89
Glycerol 3-phosphatee (357)	lipid	357	1799.3	19.67
Octadecadienoic acid (337)	lipid	337	2218.9	25.30
Octadecatrienoic acid (335)	lipid	335	2226.4	25.21
Octadecenoic acid (339)	lipid	339	2223.9	25.45
Adenine (264)	nuclic base/nucleotide	264	1879.6	21.31
Adenosine (236)	nuclic base/nucleotide	236	2680.6	22.92
Uracil (241)	nuclic base/nucleotide	241	1344.9	12.14
2-Aminoadipate (260)	organic acid	260	1742.3	23.67
2-Isopropylmalate (275)	organic acid	275	1599.2	20.56
2-Oxoglutarate (129)	organic acid	129	1597.1	16.29
4-Aminobutanoate [GABA] (174)	organic acid	174	1544.7	15.41

5-Indolecarboxylic acid (305)	organic acid	305	2033.7	22.84
Adipic acid (111)	organic acid	111	1515.5	15.02
Aminomalonic acid (218)	organic acid	218	1483.7	14.38
Azelaic acid (317)	organic acid	317	1806.9	19.64
Benzoic acid (267)	organic acid	267	1250.5	16.99
cis-Aconitate (229)	organic acid	229	1770.5	18.98
Coumaric acid (308)	organic acid	308	1807.4	19.60
Erythronic acid (292)	organic acid	292	1571.3	15.82
Gluconate (333)	organic acid	333	2037.5	23.02
Lactic acid (191)	organic acid	191	1076.6	7.32
Salicylic acid (267)	organic acid	267	1518.7	15.03
Shikimate (204)	organic acid	204	1834.8	19.99
Succinate (147)	organic acid	147	1316.3	11.56
Tartaric acid (292)	organic acid	292	1671.9	27.43
3-Caffeoyl-trans-quinic acid (345)	phenylpropanoid	345	3179	47.03
Fructose (217)	carbohydrates	217	1913.5/	
1924.8	21.23			
Glucose-6-phosphatee (387)	carbohydrates	387	2392.9	27.06
Lactose (361)	carbohydrates	361	2738.3/	
2754/				
2768.6	31.54			
Melibiose (361)	carbohydrates	361	2905.3/	
2917.9/				
2931.6	32.28			
Myo-Inositol-1-phosphatee (318)	carbohydrates	318	2486.6	16.89
Phosphoenolpyruvate (247)	carbohydrates	247	1624.7	21.11
Rhamnose (117)	carbohydrates	117	1756.3	18.74
Ribose (217)	carbohydrates	217	1709.9	18.14
Sucrose (361)	carbohydrates	361	2716.8	30.82
Xylose (217)	carbohydrates	217	1685.4/	
1694.4	17.67			
fatty acid (339) RT1518	unidentified lipid	339	-	25.30
carbohydrate (319) RT1314	unidentified carbohydrates	319	-	21.90
carbohydrate (319) RT1321	unidentified carbohydrates	319	-	22.02
carbohydrate (204) RT1781	unidentified carbohydrates	204	-	29.68
carbohydrate (204) RT1913	unidentified carbohydrates	204	-	31.88
compound (174) RT433	unidentified	174	-	7.22
compound (191) RT435	unidentified	191	-	7.25
compound (75) RT435	unidentified	75	-	7.25
compound (87) RT435	unidentified	87	-	7.25
compound (207) RT436	unidentified	207	-	7.27
compound (89) RT439	unidentified	89	-	7.32
compound (89) RT440	unidentified	89	-	7.33
compound (117) RT443	unidentified	117	-	7.38
compound (173) RT443	unidentified	173	-	7.38

compound (112) RT451	unidentified	112	-	7.52
compound (207) RT454	unidentified	207	-	7.57
compound (117) RT459	unidentified	117	-	7.65
compound (77) RT466	unidentified	77	-	7.77
compound (127) RT470	unidentified	127	-	7.83
compound (58) RT470	unidentified	58	-	7.83
compound (116) RT474	unidentified	116	-	7.90
compound (258) RT494	unidentified	258	-	8.23
compound (125) RT508	unidentified	125	-	8.47
compound (355) RT515	unidentified	355	-	8.58
compound (117) RT529	unidentified	117	-	8.82
compound (158) RT533	unidentified	158	-	8.88
compound (281) RT548	unidentified	281	-	9.13
compound (241) RT553	unidentified	241	-	9.22
compound (89) RT575	unidentified	89	-	9.58
compound (169) RT581	unidentified	169	-	9.68
compound (288) RT590	unidentified	288	-	9.83
compound (169) RT592	unidentified	169	-	9.87
compound (117) RT605	unidentified	117	-	10.08
compound (281) RT610	unidentified	281	-	10.17
compound (219) RT616	unidentified	219	-	10.27
compound (179) RT621	unidentified	179	-	10.35
compound (284) RT632	unidentified	284	-	10.53
compound (74) RT632	unidentified	74	-	10.53
compound (186) RT642	unidentified	186	-	10.70
compound (192) RT643	unidentified	192	-	10.72
compound (158) RT649	unidentified	158	-	10.82
compound (159) RT650	unidentified	159	-	10.83
compound (205) RT653	unidentified	205	-	10.88
compound (173) RT665	unidentified	173	-	11.08
compound (280) RT669	unidentified	280	-	11.15
compound (126) RT670	unidentified	126	-	11.17
compound (75) RT690	unidentified	75	-	11.50
compound (89) RT706	unidentified	89	-	11.77
compound (75) RT722	unidentified	75	-	12.03
compound (278) RT789	unidentified	278	-	13.15
compound (103) RT803	unidentified	103	-	13.38
compound (75) RT824	unidentified	75	-	13.73
compound (172) RT825	unidentified	172	-	13.75
compound (243) RT830	unidentified	243	-	13.83
compound (306) RT832	unidentified	306	-	13.87
compound (237) RT851	unidentified	237	-	14.18
compound (179) RT867	unidentified	179	-	14.45
compound (191) RT908	unidentified	191	-	15.13
compound (306) RT913	unidentified	306	-	15.22
compound (174) RT924	unidentified	174	-	15.40
compound (263) RT932	unidentified	263	-	15.53

compound (71) RT934	unidentified	71	-	15.57
compound (120) RT940	unidentified	120	-	15.67
compound (158) RT944	unidentified	158	-	15.73
compound (227) RT945	unidentified	227	-	15.75
compound (174) RT956	unidentified	174	-	15.93
compound (217) RT964	unidentified	217	-	16.07
compound (142) RT973	unidentified	142	-	16.22
compound (103) RT974	unidentified	103	-	16.23
compound (103) RT982	unidentified	103	-	16.37
compound (223) RT983	unidentified	223	-	16.38
compound (245) RT987	unidentified	245	-	16.45
compound (342) RT996	unidentified	342	-	16.60
compound (117) RT1037	unidentified	117	-	17.28
compound (245) RT1049	unidentified	245	-	17.48
compound (103) RT1055	unidentified	103	-	17.58
compound (245) RT1065	unidentified	245	-	17.75
compound (277) RT1082	unidentified	277	-	18.03
compound (117) RT1121	unidentified	117	-	18.68
compound (217) RT1123	unidentified	217	-	18.72
compound (57) RT1127	unidentified	57	-	18.78
compound (93) RT1132	unidentified	93	-	18.87
compound (103) RT1133	unidentified	103	-	18.88
compound (69) RT1140	unidentified	69	-	19.00
compound (174) RT1143	unidentified	174	-	19.05
compound (69) RT1150	unidentified	69	-	19.17
compound (217) RT1155	unidentified	217	-	19.25
compound (103) RT1166	unidentified	103	-	19.43
compound (217) RT1166	unidentified	217	-	19.43
compound (292) RT1166	unidentified	292	-	19.43
compound (295) RT1171	unidentified	295	-	19.52
compound (69) RT1171	unidentified	69	-	19.52
compound (57) RT1173	unidentified	57	-	19.55
compound (217) RT1180	unidentified	217	-	19.67
compound (292) RT1203	unidentified	292	-	20.05
compound (174) RT1212	unidentified	174	-	20.20
compound (285) RT1213	unidentified	285	-	20.22
compound (103) RT1218	unidentified	103	-	20.30
compound (75) RT1218	unidentified	75	-	20.30
compound (156) RT1226	unidentified	156	-	20.43
compound (149) RT1233	unidentified	149	-	20.55
compound (160) RT1240	unidentified	160	-	20.67
compound (103) RT1243	unidentified	103	-	20.72
compound (71) RT1257	unidentified	71	-	20.95
compound (295) RT1259	unidentified	295	-	20.98
compound (344) RT1259	unidentified	344	-	20.98
compound (103) RT1264	unidentified	103	-	21.07
compound (319) RT1268	unidentified	319	-	21.13

compound (179) RT1272	unidentified	179	-	21.20
compound (204) RT1272	unidentified	204	-	21.20
compound (217) RT1272	unidentified	217	-	21.20
compound (71) RT1278	unidentified	71	-	21.30
compound (319) RT1286	unidentified	319	-	21.43
compound (273) RT1289	unidentified	273	-	21.48
compound (273) RT1297	unidentified	273	-	21.62
compound (299) RT1298	unidentified	299	-	21.63
compound (132) RT1300	unidentified	132	-	21.67
compound (299) RT1309	unidentified	299	-	21.82
compound (285) RT1310	unidentified	285	-	21.83
compound (57) RT1314	unidentified	57	-	21.90
compound (319) RT1316	unidentified	319	-	21.93
compound (71) RT1316	unidentified	71	-	21.93
compound (160) RT1319	unidentified	160	-	21.98
compound (333) RT1325	unidentified	333	-	22.08
compound (174) RT1327	unidentified	174	-	22.12
compound (361) RT1330	unidentified	361	-	22.17
compound (318) RT1334	unidentified	318	-	22.23
compound (204) RT1340	unidentified	204	-	22.33
compound (205) RT1347	unidentified	205	-	22.45
compound (293) RT1357	unidentified	293	-	22.62
compound (311) RT1362	unidentified	311	-	22.70
compound (155) RT1366	unidentified	155	-	22.77
compound (297) RT1372	unidentified	297	-	22.87
compound (335) RT1385	unidentified	335	-	23.08
compound (331) RT1441	unidentified	331	-	24.02
compound (217) RT1443	unidentified	217	-	24.05
compound (331) RT1443	unidentified	331	-	24.05
compound (324) RT1456	unidentified	324	-	24.27
compound (327) RT1456	unidentified	327	-	24.27
compound (319) RT1465	unidentified	319	-	24.42
compound (319) RT1466	unidentified	319	-	24.43
compound (319) RT1487	unidentified	319	-	24.78
compound (128) RT1504	unidentified	128	-	25.07
compound (185) RT1512	unidentified	185	-	25.20
compound (204) RT1517	unidentified	204	-	25.28
compound (357) RT1526	unidentified	357	-	25.43
compound (204) RT1603	unidentified	204	-	26.72
compound (167) RT1609	unidentified	167	-	26.82
compound (204) RT1615	unidentified	204	-	26.92
compound (197) RT1637	unidentified	197	-	27.28
compound (239) RT1643	unidentified	239	-	27.38
compound (83) RT1643	unidentified	83	-	27.38
compound (255) RT1654	unidentified	255	-	27.57
compound (204) RT1655	unidentified	204	-	27.58
compound (57) RT1655	unidentified	57	-	27.58

compound (259) RT1657	unidentified	259	-	27.62
compound (91) RT1660	unidentified	91	-	27.67
compound (260) RT1676	unidentified	260	-	27.93
compound (204) RT1683	unidentified	204	-	28.05
compound (82) RT1700	unidentified	82	-	28.33
compound (204) RT1731	unidentified	204	-	28.85
compound (149) RT1745	unidentified	149	-	29.08
compound (204) RT1749	unidentified	204	-	29.15
compound (217) RT1751	unidentified	217	-	29.18
compound (204) RT1756	unidentified	204	-	29.27
compound (283) RT1774	unidentified	283	-	29.57
compound (219) RT1776	unidentified	219	-	29.60
compound (127) RT1780	unidentified	127	-	29.67
compound (204) RT1780	unidentified	204	-	29.67
compound (204) RT1788	unidentified	204	-	29.80
compound (216) RT1788	unidentified	216	-	29.80
compound (261) RT1791	unidentified	261	-	29.85
compound (204) RT1796	unidentified	204	-	29.93
compound (204) RT1798	unidentified	204	-	29.97
compound (117) RT1803	unidentified	117	-	30.05
compound (173) RT1809	unidentified	173	-	30.15
compound (191) RT1825	unidentified	191	-	30.42
compound (204) RT1834	unidentified	204	-	30.57
compound (204) RT1853	unidentified	204	-	30.88
compound (319) RT1860	unidentified	319	-	31.00
compound (356) RT1864	unidentified	356	-	31.07
compound (306) RT1865	unidentified	306	-	31.08
compound (217) RT1877	unidentified	217	-	31.28
compound (204) RT1902	unidentified	204	-	31.70
compound (259) RT1906	unidentified	259	-	31.77
compound (160) RT1909	unidentified	160	-	31.82
compound (361) RT1909	unidentified	361	-	31.82
compound (204) RT1914	unidentified	204	-	31.90
compound (361) RT1919	unidentified	361	-	31.98
compound (259) RT1920	unidentified	259	-	32.00
compound (361) RT1926	unidentified	361	-	32.10
compound (217) RT1936	unidentified	217	-	32.27
compound (223) RT1937	unidentified	223	-	32.28
compound (204) RT1939	unidentified	204	-	32.32
compound (361) RT1948	unidentified	361	-	32.47
compound (204) RT1958	unidentified	204	-	32.63
compound (319) RT1968	unidentified	319	-	32.80
compound (204) RT1971	unidentified	204	-	32.85
compound (83) RT1974	unidentified	83	-	32.90
compound (362) RT1978	unidentified	362	-	32.97
compound (204) RT1986	unidentified	204	-	33.10
compound (525) RT1992	unidentified	525	-	33.20

compound (201) RT1994	unidentified	201	-	33.23
compound (217) RT1994	unidentified	217	-	33.23
compound (361) RT2003	unidentified	361	-	33.38
compound (204) RT2011	unidentified	204	-	33.52
compound (119) RT2019	unidentified	119	-	33.65
compound (297) RT2022	unidentified	297	-	33.70
compound (217) RT2026	unidentified	217	-	33.77
compound (361) RT2053	unidentified	361	-	34.22
compound (91) RT2055	unidentified	91	-	34.25
compound (327) RT2065	unidentified	327	-	34.42
compound (204) RT2068	unidentified	204	-	34.47

Supplementary Table 2: Chemical richness as function of site, species and growth form. Supplementary Table shows the **A)** analysis of variance (ANOVA) and the **B)** parameter estimates of linear mixed effect models for chemical richness of polar and semi-polar metabolites. Fixed factors were either species, site and their interaction (model 1) or growth form, site and their interaction (model 2). In both models, plot was included as random factors. Note that the intercept in model 1 refers to species *Achillea millefolium* and the site ALB (Swabian Alb), and in model 2 to the growth form forb and site ALB.

a) ANOVA

Predictor		Polar metabolites *p*	Semi polar metabolites *p*
Model 1	Species	0.023	<0.001
	Site	<0.001	<0.001
	Site × species	0.004	0.001
Model 2	Growth form	0.260	0.630
	Site	<0.001	<0.001
	Site × growth form	<0.001	<0.001

b) linear mixed effect models

Predictor		Polar metabolites Estimate	*p*	Semi-polar metabolites Estimate	*p*
Model 1	Intercept	38.653	**<0.001**	92.246	**<0.001**
	G. mollugo	9.634	0.267	36.365	0.144
	G. verum	4.672	0.590	-16.707	0.502
	P. lanceolata	11.859	0.184	0.248	0.992
	R. acris	-4.749	0.618	-36.648	0.177
	A. pratensis	0.035	0.997	0.0348	0.997
	A. elatius	16.950	**0.039**	8.790	0.708
	D. glomerata	-0.269	0.974	-30.566	0.194
	L. perenne	-2.774	0.753	-41.355	0.105
	P. pratensis	-2.678	0.772	-37.423	0.154
	HAI	12.891	0.159	30.971	0.197
	SCH	20.522	0.109	-19.523	0.568
	G. mollugo HAI	5.887	0.619	78.095	**0.023**
	G. verum HAI	-5.547	0.633	21.942	0.512
	P. lanceolata HAI	-0.919	0.941	48.305	0.173
	R. acris HAI	7.867	0.529	-1.461	0.967
	A. pratensis HAI	0.148	0.992	2.580	0.950
	A. elatius HAI	-28.357	**0.030**	-47.266	0.204
	D. glomerata HAI	2.465	0.834	15.383	0.650
	L. perenne HAI	6.714	0.605	21.875	0.558
	P. pratensis HAI	-5.469	0.667	-0.914	0.980
	G. mollugo SCH	17.573	0.257	97.729	**0.028**
	G. verum SCH	14.908	0.363	139.798	**0.003**
	P. lanceolata SCH	14.081	0.389	65.797	0.159
	R. acris SCH	55.602	**0.006**	96.651	**0.093**
	A. pratensis SCH	35.197	0.058	145.843	**0.005**
	A. elatius SCH	42.217	**0.009**	164.544	**<0.001**
	D. glomerata SCH	43.128	**0.004**	134.429	**0.002**
	L. perenne SCH	57.209	**<0.001**	151.172	**0.001**
	P. pratensis SCH	34.557	**0.037**	98.084	**0.038**
Model 2	Intercept	43.269	**<0.001**	88.58	**0.001**
	grass	-1.749	0.729	-19.95	0.392
	HAI	13.748	**0.014**	59.540	**<0.001**
	SCH	37.333	**<0.001**	60.210	**<0.001**
	grass: HAI	-5.493	0.353	-28.410	0.095
	grass: SCH	25.192	**<0.001**	57.330	**0.005**

Supplementary Table 3: Probability of occurrence of polar metabolite. Values represent the ratio of the percentage of occurrence of the sample groups, A) growth forms, the sites and their interaction, B) the species, and C) the interaction between species and sites. Metabolites occurring with an at least to times higher percentage in one of the groups are colored green.

Asterisk marks all metabolites, which occur only in the corresponding sample group. Metabolites being not preferentially exuded by at least one sample group are left out.

a) Growth form, site, interaction of site and growth form

	growth form		site			site x growth form					
**Metabolites**	**forb**	**grass**	**ALB**	**HAI**	**SCH**	**ALB forb**	**ALB grass**	**HAI forb**	**HAI grass**	**SCH forb**	**SCH grass**
Noradrenalin (174)	0	2 *	2 *	0	0	0	2 *	0	0	0	0
Threitol (217)	0.19	5.19	0.25	1.06	0.4	0	0.22	0.04	1.05	0.24	0.08
N-Acetylglucosamine (156)	0	2 *	2 *	0	0	0	2 *	0	0	0	0
Tyramine (174)	0.45	2.23	0.34	0.35	0.94	0.33	0	0	0.48	0	0.75
Asparagine (231)	2 *	0	2 *	0	0	2 *	0	0	0	0	0
Aspartate (232)	0.51	1.95	0.23	0.38	1.16	0.08	0.13	0.1	0.26	0.23	0.49
beta-Alanine (248)	0.04	22.26	0.19	0.2	2.07	0.03	0.16	0	0.27	0	1.63
Glutamate (246)	0.49	2.04	0.24	0.32	1.32	0.06	0.16	0.1	0.2	0.25	0.54
Glutamine (155)	1.8	0.56	0.49	2.06	0	0	0.59	1.7	0	0	0
Homoserine (218)	0.05	18.92	0.13	0.31	1.87	0	0.13	0.03	0.33	0	1.48
Lysine (156)	0.36	2.78	0.26	0.27	1.41	0	0.3	0.24	0	0	1.36
Methionine (176)	0.12	8.68	0.13	0.16	2.99	0.01	0.12	0	0.21	0.15	1.37
Phenylalanine (192)	0.26	3.9	0.18	0.25	1.82	0.02	0.16	0.06	0.21	0.17	0.89
Tyrosine (218)	0.08	12.98	0.11	0.21	2.58	0	0.12	0.02	0.26	0.08	1.48

Glycerol 3-phosphate (357)	1.3	0.77	0.09	0.13	4.02	0.06	0.03	0.11	0	0.77	0.62
Octadecadienoic acid (337)	0.25	4.08	0.04	0	24.82	0	0.04	0	0	0.37	2.24
Octadecatrienoic acid (335)	3.59	0.28	0	0.81	1.24	0	0	0.67	0	0.51	0.36
Octadecenoic acid (339)	0.47	2.14	0.23	0.18	1.94	0.05	0.16	0.05	0.14	0.4	0.57
Uracil (241)	0.34	2.98	0.21	0.33	1.38	0.02	0.17	0.07	0.26	0.25	0.54
2-Aminoadipate (260)	0.15	6.68	0.26	0.27	1.41	0	0.28	0.1	0.16	0	1.22
2-Isopropylmalate (275)	2.92	0.34	0	2 *	0	0	0	2.18	0.46	0	0
Aminomalonic acid (218)	0	2 *	0	0	2 *	0	0	0	0	0	2 *
Azelaic acid (317)	7.19	0.14	0	4.31	0.23	0	0	1.74	0.16	0.3	0
Coumaric acid (308)	0	2 *	0	0.13	7.42	0	0	0	0.19	0	5.29
Lactic acid (191)	2.1	0.48	0.11	9.26	0	0.11	0	1.07	0.62	0	0
Succinate (147)	0	2 *	2 *	0	0	0	2 *	0	0	0	0
Tartaric acid (292)	0.9	1.11	0	0.13	7.42	0	0	0.11	0	0.64	1.02
3-Caffeoyl-trans-quinic acid (345)	2 *	0	0	2 *	0	0	0	2 *	0	0	0
Glucose-6-phosphate (387)	1.35	0.74	0	0.36	2.78	0	0	0.31	0	0.43	0.87
Lactose (361)	3.74	0.27	0.54	0.34	0.66	0.38	0.09	0.28	0	0.41	0.16
Melibiose (361)	4.94	0.2	0.08	3.89	0.15	0.08	0	1.35	0.23	0.19	0
Myo-Inositol-1-phosphate (318)	0.54	1.85	0	0.08	12.98	0	0	0	0.1	0.81	0.86
Phosphoenolpyruvate (247)	0.16	6.23	0.36	0.04	2.28	0.07	0.27	0	0.06	0.1	1.35
Sucrose (361)	0.9	1.11	2 *	0	0	0.98	1.02	0	0	0	0
Xylose (217)	1.71	0.59	0.03	5.55	0.14	0.03	0	1.06	0.52	0	0.13
unknown compound (174) RT433	0	2 *	2 *	0	0	0	2 *	0	0	0	0
unknown compound (191) RT435	1.35	0.74	0.08	0	12.41	0	0.08	0	0	2.24	0.31
unknown compound (75) RT435	0	2 *	2 *	0	0	0	2 *	0	0	0	0
unknown compound (89) RT439	4.49	0.22	0.11	2.48	0.23	0.12	0	1.23	0.16	0.13	0.1

unknown compound (117) RT443	0.78	1.28	0.02	0.36	2.58	0	0.02	0.14	0.17	0.53	0.59
unknown compound (173) RT443	2 *	0	0	0	2 *	0	0	0	0	2 *	0
unknown compound (112) RT451	0	2 *	2 *	0	0	0	2 *	0	0	0	0
unknown compound (117) RT459	0.82	1.21	5.5	0	0.18	0.81	0.71	0	0	0	0.16
unknown compound (77) RT466	0	2 *	0.08	0	12.41	0	0.09	0	0	0	10.9
unknown compound (127) RT470	0	2 *	2 *	0	0	0	2 *	0	0	0	0
unknown compound (58) RT470	0	2 *	2 *	0	0	0	2 *	0	0	0	0
unknown compound (258) RT494	0	2 *	2 *	0	0	0	2 *	0	0	0	0
unknown compound (125) RT508	2 *	0	2 *	0	0	2 *	0	0	0	0	0
unknown compound (117) RT529	0.08	12.8	0	0.1	9.74	0	0	0.02	0.1	0.06	4.92
unknown compound (158) RT533	0.78	1.28	0.13	0.13	3.39	0.05	0.07	0.07	0.05	0.59	0.67
unknown compound (241) RT553	1.2	0.83	0	0.4	2.47	0	0	0.26	0.07	0.47	0.7
unknown compound (89) RT575	0.67	1.48	0	0	2 *	0	0	0	0	0.97	1.03
unknown compound (288) RT590	0	2 *	2 *	0	0	0	2 *	0	0	0	0
unknown compound (169) RT592	4.04	0.25	0.07	0.49	1.55	0.07	0	0.39	0	0.76	0.29
unknown compound (179) RT621	0.9	1.11	0.07	0.34	2.1	0.03	0.04	0.17	0.11	0.44	0.6
unknown compound (284) RT632	2 *	0	0	2 *	0	0	0	2 *	0	0	0
unknown compound (74) RT632	0	2 *	0	2 *	0	0	0	0	2 *	0	0
unknown compound (186) RT642	0.78	1.28	0.03	0.27	3.14	0	0.03	0.15	0.08	0.49	0.77
unknown compound (192) RT643	2 *	0	0	2 *	0	0	0	2 *	0	0	0
unknown compound (158) RT649	0.22	4.45	1.46	0.69	0	0.22	0.57	0	0.85	0	0
unknown compound (159) RT650	0.48	2.09	0.13	2.72	0.18	0.04	0.08	0.29	1.13	0	0.15
unknown compound (173) RT665	0	2 *	2 *	0	0	0	2 *	0	0	0	0
unknown compound (280) RT669	0.82	1.21	0.04	0.33	2.42	0.01	0.03	0.16	0.13	0.46	0.64
unknown compound (126) RT670	2 *	0	0	0.54	1.85	0	0	0.39	0	2.54	0

unknown compound (75) RT690	0.74	1.36	0.05	0.12	5.61	0	0.05	0.07	0.04	0.74	0.75
unknown compound (89) RT706	0.64	1.56	0.12	3.95	0.11	0.05	0.05	0.38	1.17	0	0.09
unknown compound (278) RT789	0.26	3.9	0.07	0.17	3.74	0.07	0	0	0.22	0.18	1.48
unknown compound (306) RT832	2 *	0	0	0.36	2.78	0	0	0.26	0	3.81	0
unknown compound (179) RT867	1.11	0.9	0.14	0.24	2.18	0.08	0.04	0.1	0.11	0.63	0.43
unknown compound (191) RT908	0.45	2.23	1.94	0.51	0	0.43	0.44	0	0.64	0	0
unknown compound (306) RT913	0	2 *	0.26	0	3.82	0	0.3	0	0	0	3.35
unknown compound (174) RT924	3.77	0.26	0.17	0.66	0.83	0.14	0.03	0.46	0.04	0.5	0.18
unknown compound (71) RT934	0.07	14.47	0	0.04	24.11	0	0	0	0.06	0.1	5.86
unknown compound (158) RT944	1.04	0.96	0.09	0.21	2.89	0.05	0.04	0.14	0.05	0.56	0.65
unknown compound (227) RT945	1.32	0.76	0.04	9.21	0.06	0.02	0.02	0.86	0.79	0.02	0.04
unknown compound (174) RT956	0.13	7.79	0.31	0	3.18	0	0.34	0	0	0.22	1.3
unknown compound (217) RT964	0.46	2.15	0.18	0.24	1.88	0.08	0.08	0.04	0.22	0.31	0.64
unknown compound (142) RT973	0.68	1.47	0.1	0.31	2.02	0.03	0.08	0.16	0.11	0.33	0.71
unknown compound (103) RT974	0.3	3.34	0.08	0.08	5.64	0	0.08	0	0.11	0.48	1.02
unknown compound (103) RT982	0.6	1.67	0.02	0.29	3.15	0	0.02	0.1	0.16	0.47	0.76
unknown compound (223) RT983	0.1	9.57	0.12	0.16	3.13	0	0.12	0.01	0.19	0.14	1.51
unknown compound (245) RT987	0.1	10.02	0.06	0	17.18	0	0.06	0	0	0.15	4.24
unknown compound (342) RT996	1.7	0.59	0.08	12.35	0	0	0.09	1.33	0.54	0	0
unknown compound (245) RT1049	1.57	0.64	0.1	10.29	0	0.1	0	0.84	0.83	0	0
unknown compound (103) RT1055	1.25	0.8	0.04	5.06	0.14	0.04	0	0.68	0.77	0.05	0.08
unknown compound (245) RT1065	0	2 *	2 *	0	0	0	2 *	0	0	0	0
unknown compound (217) RT1123	2 *	0	2 *	0	0	2 *	0	0	0	0	0
unknown compound (57) RT1127	0.61	1.63	0	0.37	2.72	0	0	0.19	0.13	0.25	1.11
unknown compound (93) RT1132	2 *	0	0	2 *	0	0	0	2 *	0	0	0

unknown compound (69) RT1140	0.28	3.56	0.21	0.11	2.69	0.03	0.18	0.03	0.09	0.26	1.01
unknown compound (174) RT1143	1.8	0.56	0.26	0	3.82	0.2	0.04	0	0	1.06	0.4
unknown compound (217) RT1155	0	2 *	0.07	0.07	7.06	0	0.07	0	0.09	0	5.55
unknown compound (295) RT1171	0.58	1.73	0.13	0.03	6.04	0.05	0.07	0	0.03	0.65	0.84
unknown compound (69) RT1171	0.36	2.78	0	2 *	0	0	0	0.27	3.72	0	0
unknown compound (217) RT1180	2.95	0.34	0.2	0.32	1.44	0.17	0.02	0.23	0.03	0.72	0.25
unknown compound (292) RT1203	0.49	2.05	0.25	0.24	1.54	0.08	0.16	0.11	0.12	0.19	0.77
unknown compound (174) RT1212	0.9	1.11	0.52	0	1.91	0	0.46	0	0	2.17	0
unknown compound (103) RT1218	0	2 *	2 *	0	0	0	2 *	0	0	0	0
unknown compound (75) RT1218	0.16	6.2	0.36	0.17	1.45	0.07	0.26	0.01	0.19	0.07	0.94
unknown compound (156) RT1226	0.49	2.05	0.18	0.08	3.49	0.04	0.14	0.05	0.02	0.44	0.89
unknown compound (149) RT1233	0.84	1.19	0.24	0.12	2.29	0.09	0.12	0.08	0.03	0.5	0.58
unknown compound (160) RT1240	0.39	2.55	0.41	0.21	1.16	0.13	0.22	0.06	0.16	0.12	0.68
unknown compound (71) RT1257	2 *	0	0.42	0.58	0.51	0.42	0	0.45	0	0.65	0
unknown compound (295) RT1259	0.45	2.23	2 *	0	0	0.49	2.04	0	0	0	0
unknown compound (344) RT1259	2 *	0	0	2 *	0	0	0	2 *	0	0	0
unknown compound (103) RT1264	2.37	0.42	0.85	0.03	1.04	0.39	0.19	0.03	0	0.87	0.08
unknown compound (319) RT1268	0.25	4.08	0	0.04	24.11	0	0	0	0.05	0.36	2.16
unknown compound (179) RT1272	0	2 *	2 *	0	0	0	2 *	0	0	0	0
unknown compound (204) RT1272	0.9	1.11	0	2 *	0	0	0	0.67	1.49	0	0
unknown compound (217) RT1272	0.06	16.69	0.07	0	13.36	0	0.08	0	0	0.09	5.23
unknown compound (273) RT1297	0.83	1.2	0.08	0.31	2.22	0.03	0.04	0.12	0.16	0.55	0.49
unknown compound (299) RT1298	2 *	0	0	2 *	0	0	0	2 *	0	0	0
unknown compound (132) RT1300	0.71	1.4	0.1	0.29	2.2	0.02	0.07	0.11	0.14	0.5	0.54
unknown compound (299) RT1309	3.95	0.25	0.41	0.37	0.79	0.42	0	0.26	0.04	0.35	0.25

unknown compound (285) RT1310	0	2 *	2 *	0	0	0	2 *	0	0	0	0
unknown compound (57) RT1314	0.9	1.11	0.21	2.52	0.12	0.06	0.13	0.54	0.6	0	0.11
unknown compound (319) RT1316	0.73	1.37	0.24	0.15	2.1	0.1	0.12	0.08	0.05	0.38	0.66
unknown compound (71) RT1316	2.7	0.37	0.32	3.09	0	0.33	0	0.76	0.47	0	0
unknown compound (160) RT1319	2.4	0.42	0.3	0.26	1.3	0.29	0	0.13	0.09	0.55	0.28
unknown compound (333) RT1325	0.58	1.73	0.24	0.06	2.95	0.06	0.16	0.02	0.04	0.58	0.61
unknown compound (318) RT1334	1.2	0.83	0.04	0	24.82	0.04	0	0	0	1.37	0.62
unknown compound (204) RT1340	0.04	27.82	0.16	0.02	5.16	0	0.18	0	0.03	0.06	3.2
unknown compound (293) RT1357	0.1	10.02	0.03	0.03	16.93	0	0.03	0	0.04	0.15	4.07
unknown compound (311) RT1362	0.7	1.43	0.12	0.21	2.54	0.05	0.07	0.09	0.1	0.46	0.66
unknown compound (155) RT1366	1.8	0.56	0	0.27	3.71	0	0	0	0.29	3.42	0
unknown compound (297) RT1372	0	2 *	0	0	2 *	0	0	0	0	0	2 *
unknown compound (335) RT1385	2.9	0.35	0.24	0.12	2.33	0.23	0	0.1	0	0.84	0.36
unknown compound (331) RT1441	0.45	2.23	1.05	0	0.95	0.37	0.38	0	0	0	0.85
unknown compound (331) RT1443	0.63	1.59	0.21	4.8	0	0	0.2	0.5	0.99	0	0
unknown compound (324) RT1456	0.4	2.53	0.13	0	7.91	0.02	0.11	0	0	0.56	1.11
unknown compound (327) RT1456	0.86	1.17	0.13	0.06	4.88	0.03	0.09	0.04	0.02	0.94	0.54
unknown compound (319) RT1465	2.4	0.42	0.36	2.75	0	0.11	0.24	1.4	0.14	0	0
unknown compound (319) RT1466	0.57	1.74	0.17	0.21	2.08	0.08	0.09	0.1	0.1	0.26	0.85
unknown compound (319) RT1487	0.74	1.35	0.02	0.19	4.55	0	0.02	0.06	0.12	0.78	0.59
unknown compound (128) RT1504	0.11	8.9	0	1.89	0.53	0	0	0.08	1.81	0	0.4
unknown compound (185) RT1512	0	2 *	0	2 *	0	0	0	0	2 *	0	0
unknown compound (204) RT1603	1.05	0.95	0.08	0.23	2.81	0.01	0.07	0.08	0.12	1.05	0.3
unknown compound (204) RT1615	0.07	13.35	0	0.1	10.2	0	0	0	0.13	0.11	3.57
unknown compound (197) RT1637	0.6	1.67	0.03	0.3	2.79	0	0.03	0.13	0.14	0.4	0.79

unknown compound (239) RT1643	0	2 *	2 *	0	0	0	2 *	0	0	0	0
unknown compound (83) RT1643	0	2 *	2 *	0	0	0	2 *	0	0	0	0
unknown compound (204) RT1655	0.9	1.11	0.24	0.06	2.95	0.14	0.07	0.01	0.06	0.65	0.54
unknown compound (57) RT1655	1.62	0.62	0.21	0	4.77	0.2	0	0	0	0.88	0.57
unknown compound (259) RT1657	0.63	1.59	0.23	0.06	3.06	0.08	0.13	0.03	0.02	0.5	0.72
unknown compound (91) RT1660	0	2 *	2 *	0	0	0	2 *	0	0	0	0
unknown compound (260) RT1676	1.11	0.9	0.32	0.53	0.7	0.15	0.13	0.22	0.19	0.28	0.25
unknown compound (204) RT1683	0	2 *	2 *	0	0	0	2 *	0	0	0	0
unknown compound (204) RT1731	0.8	1.25	0.06	0.25	2.8	0.02	0.05	0.11	0.12	0.61	0.56
unknown compound (204) RT1749	2.52	0.4	1.01	0.31	0.35	0.61	0.17	0.26	0	0.12	0.19
unknown compound (217) RT1751	20.67	0.05	0.26	0.86	0.49	0.27	0	0.68	0	0.46	0.07
unknown compound (204) RT1756	8.99	0.11	1.19	0.19	0.42	0.84	0.08	0.15	0	0.5	0
unknown compound (283) RT1774	0.78	1.28	0	0.22	4.64	0	0	0.13	0.06	0.51	0.96
unknown compound (219) RT1776	2.44	0.41	0.25	0.43	0.99	0.22	0.03	0.3	0.04	0.36	0.34
unknown compound (127) RT1780	7.19	0.14	3.4	0.29	0	2.18	0.13	0.25	0	0	0
unknown compound (204) RT1780	0	2 *	2 *	0	0	0	2 *	0	0	0	0
unknown compound (204) RT1788	2 *	0	0	0	2 *	0	0	0	0	2 *	0
unknown compound (216) RT1788	2 *	0	0.23	0.4	1.13	0.22	0	0.3	0	1.44	0
unknown compound (261) RT1791	4.35	0.23	0.2	0.64	0.81	0.16	0.03	0.52	0	0.44	0.22
unknown compound (204) RT1796	2 *	0	1.7	0.15	0.32	1.62	0	0.12	0	0.38	0
unknown compound (204) RT1798	1.38	0.72	0.16	0.2	2.26	0.14	0.02	0.12	0.06	0.55	0.53
unknown compound (117) RT1803	0.77	1.3	0.03	0.23	3.52	0.01	0.02	0.1	0.11	0.62	0.65
unknown compound (173) RT1809	9.52	0.1	0.3	0.5	0.78	0.25	0.03	0.36	0.02	0.79	0.05
unknown compound (191) RT1825	0.81	1.24	0.17	0.22	2.09	0.08	0.08	0.15	0.05	0.32	0.78
unknown compound (204) RT1834	1.28	0.78	0.06	5.31	0.12	0.06	0	0.75	0.74	0	0.1

unknown compound (204) RT1853	36.84	0.03	0.32	0.83	0.44	0.29	0.02	0.64	0	0.57	0
unknown compound (319) RT1860	0	2 *	1.05	0	0.95	0	1.19	0	0	0	0.84
unknown compound (356) RT1864	2 *	0	0	2 *	0	0	0	2 *	0	0	0
unknown compound (204) RT1902	17.07	0.06	0.34	0.43	0.81	0.3	0.02	0.33	0	0.88	0.03
unknown compound (204) RT1914	0.66	1.52	0.21	2.22	0.16	0.04	0.16	0.43	0.68	0	0.13
unknown compound (361) RT1919	0.95	1.05	0.24	0.08	2.78	0.16	0.06	0.03	0.05	0.53	0.63
unknown compound (217) RT1936	2 *	0	0	2 *	0	0	0	2 *	0	0	0
unknown compound (223) RT1937	1.47	0.68	0.03	3.83	0.21	0.03	0	0.76	0.57	0.07	0.12
unknown compound (204) RT1939	3.14	0.32	0.12	8.24	0	0.13	0	1.45	0.42	0	0
unknown compound (204) RT1958	2.7	0.37	0.03	5.34	0.14	0.04	0	1.3	0.36	0.08	0.06
unknown compound (204) RT1971	0.85	1.17	0.03	0.21	3.88	0.03	0	0.09	0.09	0.58	0.74
unknown compound (83) RT1974	4.49	0.22	0.4	0.3	0.94	0.41	0	0.25	0	0.4	0.29
unknown compound (204) RT1986	7.19	0.14	0.28	3.6	0	0.14	0.14	3.01	0	0	0
unknown compound (525) RT1992	2 *	0	0.49	2.06	0	0.58	0	1.74	0	0	0
unknown compound (201) RT1994	3.77	0.26	0.49	0.27	0.84	0.34	0.06	0.14	0.08	0.79	0.05
unknown compound (217) RT1994	0.97	1.03	0.28	0.13	2.05	0.2	0.06	0.08	0.04	0.32	0.74
unknown compound (361) RT2003	2 *	0	0	0	2 *	0	0	0	0	2 *	0
unknown compound (297) RT2022	2.88	0.35	0.31	1.49	0.2	0.32	0	0.57	0.29	0.11	0.08
unknown compound (217) RT2026	4.59	0.22	0.3	0.31	1.16	0.28	0.01	0.25	0	0.63	0.23
unknown compound (361) RT2053	2 *	0	0.32	3.09	0	0.38	0	2.61	0	0	0
unknown compound (91) RT2055	2.25	0.45	0	2 *	0	0	0	1.68	0.6	0	0
unknown compound (204) RT2068	2 *	0	0.46	1	0.23	0.5	0	0.8	0	0.29	0
unknown compound (362) RT2071	2 *	0	0.19	5.15	0	0.23	0	4.34	0	0	0
unknown compound (91) RT2097	6.29	0.16	0.06	2.78	0.27	0.06	0	1.68	0.08	0.15	0.11
unknown compound (204) RT2147	2 *	0	0.2	0.88	0.58	0.2	0	0.67	0	0.75	0

unknown compound (153) RT2157	41.33	0.02	0.31	0.59	0.64	0.28	0.02	0.45	0	0.82	0
unknown compound (191) RT2175	0.85	1.17	0.07	0.23	2.99	0.07	0	0.18	0.02	0.24	1.3
unknown compound (204) RT2329	0	2 *	2 *	0	0	0	2 *	0	0	0	0

b) Species

	Species									
	Ac.mi.	Ga.mo.	Ga.ve.	Pl.la.	Ra.ac.	Al.pr.	Ar.el.	Da.gl.	Lo.pe.	Po.pr.
Noradrenalin (174)	0	0	0	0	0	0	2*	0	0	0
N-Acetylglucosamine (156)	0	0	0	0	0	0	0	0	0	2*
Asparagine (231)	2*	0	0	0	0	0	0	0	0	0
Octadecatrienoic acid (335)	0	0	0	5.44	0	0	0	0.18	0	0
2-Aminoadipate (260)	0	0.16	0	0	0	0	2.16	0	0.22	0
3-Caffeoyl-trans-quinic acid (345)	0	2*	0	0	0	0	0	0	0	0
Succinate (147)	0	0	0	0	0	0	0	0	2*	0
unknown compound (306) RT832	0	0	0	0	2*	0	0	0	0	0
unknown compound (174) RT433	0	0	0	0	0	2	0	0.5	0	0
unknown compound (75) RT435	0	0	0	0	0	2	0	0.5	0	0
unknown compound (112) RT451	0	0	0	0	0	2	0	0.5	0	0
unknown compound (127) RT470	0	0	0	0	0	2	0	0.5	0	0
unknown compound (58) RT470	0	0	0	0	0	2	0	0.5	0	0
unknown compound (116) RT474	0	0	0	2*	0	0	0	0	0	0
unknown compound (258) RT494	0	0	0	0	0	0	0	0	0	2*
unknown compound (125) RT508	0	0	0	0	2*	0	0	0	0	0
unknown compound (288) RT590	0	0	0	0	0	2*	0	0	0	0

unknown compound (169) RT592	0	0	0	0	5.91	0	0	0.17	0	0
unknown compound (284) RT632	0	0	0	0	2*	0	0	0	0	0
unknown compound (74) RT632	0	0	0	0	0	0	0	0	2*	0
unknown compound (192) RT643	0	0	0	0	2*	0	0	0	0	0
unknown compound (173) RT665	0	0	0	0	0	2	0	0.5	0	0
unknown compound (126) RT670	0	0	0	0	2*	0	0	0	0	0
unknown compound (191) RT908	0.43	0	0	0	0	0	0	0	0	2.33
unknown compound (174) RT956	0	0	0	0.14	0	0	7	0	0	0
unknown compound (245) RT1065	0	0	0	0	0	2	0	0.5	0	0
unknown compound (217) RT1123	0	2*	0	0	0	0	0	0	0	0
unknown compound (93) RT1132	2*	0	0	0	0	0	0	0	0	0
unknown compound (174) RT1212	0	0	0	0	0	0	2*	0	0	0
unknown compound (103) RT1218	0	0	0	0	0	2	0	0.5	0	0
unknown compound (71) RT1257	10.68	0	0	0	0.09	0	0	0	0	0
unknown compound (344) RT1259	0	0	0	2*	0	0	0	0	0	0
unknown compound (179) RT1272	0	0	0	0	0	0	0	2*	0	0
unknown compound (299) RT1298	0	0.27	0	3.72	0	0	0	0	0	0
unknown compound (299) RT1309	0	0	0.07	3.41	0	0	0.04	0.09	0.04	0
unknown compound (285) RT1310	0	0	0	0	0	2	0	0.5	0	0
unknown compound (185) RT1512	0	0	0	0	0	0	2*	0	0	0
unknown compound (239) RT1643	0	0	0	0	0	2	0	0.5	0	0
unknown compound (83) RT1643	0	0	0	0	0	2	0	0.5	0	0
unknown compound (91) RT1660	0	0	0	0	0	2	0	0.5	0	0
unknown compound (204) RT1683	0	0	0	0	0	2	0	0.5	0	0

unknown compound (204) RT1780	0	0	0	0	0	2	0	0.5	0	0
unknown compound (204) RT1788	0	0	0	0	2*	0	0	0	0	0
unknown compound (216) RT1788	0	0	0	0	2*	0	0	0	0	0
unknown compound (319) RT1860	0	0	0	0	0	0	2.72	0.37	0	0
unknown compound (356) RT1864	0	0	0	2*	0	0	0	0	0	0
unknown compound (217) RT1936	0	0	0	2*	0	0	0	0	0	0
unknown compound (83) RT1974	0	0	0.12	2.73	0	0	0.04	0.03	0.05	0.05
unknown compound (525) RT1992	0	0	0.4	2.48	0	0	0	0	0	0
unknown compound (201) RT1994	0	0.03	0	2.48	0.09	0	0	0.1	0	0.09
unknown compound (361) RT2003	0	0	2*	0	0	0	0	0	0	0
unknown compound (361) RT2053	0	0	0.27	3.72	0	0	0	0	0	0
unknown compound (362) RT2071	0	0	0.4	2.48	0	0	0	0	0	0
unknown compound (204) RT2329	0	0	0	0	0	2	0	0.5	0	0

c) Interaction of site and species

	Site x species																			
	AEG_Ac.mi.	AEG_Ga.mo.	AEG_Ga.ve.	AEG_Pl.la.	AEG_Ra.ac.	AEG_Al.pr.	AEG_Ar.el.	AEG_Da.gl.	AEG_Lo.pe.	AEG_Po.pr.	HEG_Ac.mi.	HEG_Ga.mo.	HEG_Ga.ve.	HEG_Pl.la.	HEG_Ra.ac.	HEG_Al.pr.	HEG_Ar.el.	HEG_Da.gl.	HEG_Lo.pe.	HEG_Po.pr.
Noradrenalin (174)	0	0	0	0	0	0	2*	0	0	0	0	0	0	0	0	0	0	0	0	0
N-Acetylglucosamine (156)	0	0	0	0	0	0	0	0	0	2*	0	0	0	0	0	0	0	0	0	0
Asparagine (231)	2*	0	0	0	0	0	0	0	0	0	0	0	0	0	0	0	0	0	0	0
3-Caffeoyl-trans-quinic acid (345)	0	0	0	0	0	0	0	0	0	0	0	2*	0	0	0	0	0	0	0	0
Succinate (147)	0	0	0	0	0	0	0	0	2*	0	0	0	0	0	0	0	0	0	0	0
unknown compound (174) RT433	0	0	0	0	0	2.8	0	0.36	0	0	0	0	0	0	0	0	0	0	0	0
unknown compound (75) RT435	0	0	0	0	0	2.8	0	0.36	0	0	0	0	0	0	0	0	0	0	0	0
unknown compound (112) RT451	0	0	0	0	0	2.8	0	0.36	0	0	0	0	0	0	0	0	0	0	0	0
unknown compound (127) RT470	0	0	0	0	0	2.8	0	0.36	0	0	0	0	0	0	0	0	0	0	0	0
unknown compound (58) RT470	0	0	0	0	0	2.8	0	0.36	0	0	0	0	0	0	0	0	0	0	0	0

unknown compound (116) RT474	0	0	0	0	0	0	0	0	0	0	0	0	0	0.7	0	0	0	0	0	0
unknown compound (258) RT494	0	0	0	0	0	0	0	0	0	2*	0	0	0	0	0	0	0	0	0	0
unknown compound (125) RT508	0	0	0	0	2*	0	0	0	0	0	0	0	0	0	0	0	0	0	0	0
unknown compound (288) RT590	0	0	0	0	0	2*	0	0	0	0	0	0	0	0	0	0	0	0	0	0
unknown compound (284) RT632	0	0	0	0	0	0	0	0	0	0	0	0	0	0	2*	0	0	0	0	0
unknown compound (74) RT632	0	0	0	0	0	0	0	0	0	0	0	0	0	0	0	0	0	0	2*	0
unknown compound (192) RT643	0	0	0	0	0	0	0	0	0	0	0	0	0	0	2*	0	0	0	0	0
unknown compound (173) RT665	0	0	0	0	0	2.8	0	0.36	0	0	0	0	0	0	0	0	0	0	0	0
unknown compound (245) RT987	0	0	0	0	0	0	0	0.06	0	0	0	0	0	0	0	0	0	0	0	0
unknown compound (245) RT1065	0	0	0	0	0	2.8	0	0.36	0	0	0	0	0	0	0	0	0	0	0	0
unknown compound (217) RT1123	0	2*	0	0	0	0	0	0	0	0	0	0	0	0	0	0	0	0	0	0
unknown compound (93) RT1132	0	0	0	0	0	0	0	0	0	0	2*	0	0	0	0	0	0	0	0	0
unknown compound (103) RT1218	0	0	0	0	0	2.8	0	0.36	0	0	0	0	0	0	0	0	0	0	0	0

unknown compound (344) RT1259	0	0	0	0	0	0	0	0	0	0	0	0	0	2*	0	0	0	0	0	0
unknown compound (179) RT1272	0	0	0	0	0	0	0	2*	0	0	0	0	0	0	0	0	0	0	0	0
unknown compound (299) RT1298	0	0	0	0	0	0	0	0	0	0	0	0.28	0	3.6	0	0	0	0	0	0
unknown compound (285) RT1310	0	0	0	0	0	2.8	0	0.36	0	0	0	0	0	0	0	0	0	0	0	0
unknown compound (155) RT1366	0	0	0	0	0	0	0	0	0	0	0	0	0	0	0	0	0	0	0	0.22
unknown compound (185) RT1512	0	0	0	0	0	0	0	0	0	0	0	0	0	0	0	0	2*	0	0	0
unknown compound (239) RT1643	0	0	0	0	0	2.8	0	0.36	0	0	0	0	0	0	0	0	0	0	0	0
unknown compound (83) RT1643	0	0	0	0	0	2.8	0	0.36	0	0	0	0	0	0	0	0	0	0	0	0
unknown compound (91) RT1660	0	0	0	0	0	2.8	0	0.36	0	0	0	0	0	0	0	0	0	0	0	0
unknown compound (204) RT1683	0	0	0	0	0	2.8	0	0.36	0	0	0	0	0	0	0	0	0	0	0	0
unknown compound (283) RT1774	0	0	0	0	0	0	0	0	0	0	0.04	0.02	0.06	0	0	0	0.04	0	0.04	0
unknown compound (204) RT1780	0	0	0	0	0	2.8	0	0.36	0	0	0	0	0	0	0	0	0	0	0	0
unknown compound (356) RT1864	0	0	0	0	0	0	0	0	0	0	0	0	0	2*	0	0	0	0	0	0

unknown compound (217) RT1936	0	0	0	0	0	0	0	0	0	0	0	0	0	2*	0	0	0	0	0	0
unknown compound (525) RT1992	0	0	0.42	0	0	0	0	0	0	0	0	0	0	2.4	0	0	0	0	0	0
unknown compound (361) RT2003	0	0	0	0	0	0	0	0	0	0	0	0	0	0	0	0	0	0	0	0
unknown compound (361) RT2053	0	0	0.28	0	0	0	0	0	0	0	0	0	0	3.6	0	0	0	0	0	0
unknown compound (362) RT2071	0	0	0.18	0	0	0	0	0	0	0	0	0	0.15	2.58	0	0	0	0	0	0
unknown compound (204) RT2329	0	0	0	0	0	2.8	0	0.36	0	0	0	0	0	0	0	0	0	0	0	0

Site x species

	SEG_Ac.mi.	SEG_Ga.mo.	SEG_Ga.ve.	SEG_Pl.la.	SEG_Ra.ac.	SEG_Al.pr.	SEG_Ar.el.	SEG_Da.gl.	SEG_Lo.pe.	SEG_Po.pr.
Noradrenalin (174)	0	0	0	0	0	0	0	0	0	0
N-Acetylglucosamine (156)	0	0	0	0	0	0	0	0	0	0
Asparagine (231)	0	0	0	0	0	0	0	0	0	0
3-Caffeoyl-trans-quinic acid (345)	0	0	0	0	0	0	0	0	0	0

Succinate (147)	0	0	0	0	0	0	0	0	0	0
unknown compound (174) RT433	0	0	0	0	0.23	0	0	0	0	0
unknown compound (75) RT435	0	0	0	0	0	0	0	0	0	0
unknown compound (112) RT451	0	0	0	0	0.8	0	0	0	0	0
unknown compound (127) RT470	0	0	0	0	0	0	0	0	0	0
unknown compound (58) RT470	0	0	0	0	2*	0	0	0	0	0
unknown compound (116) RT474	0	0	0	0	2	0	0	0	1.43	0
unknown compound (258) RT494	0	0	0	0	0.04	0	0	0	0	0
unknown compound (125) RT508	0	0	0	0	0	0	0	0	0	0
unknown compound (288) RT590	0	0	0	0	0	0	0	0	0	0
unknown compound (284) RT632	0	0	0	0	0.05	0	0	0	0	0
unknown compound (74) RT632	0	0	0	0	0	0	0	0	0	0
unknown compound (192) RT643	0	0	0	0	0.07	0	0	0	0	0

unknown compound (173) RT665	0	0	0	0	0	0	0	0	0	0
unknown compound (245) RT987	0	0.12	0	0	6	0	0.18	0.51	0.28	0.15
unknown compound (245) RT1065	0	0	0	0	0	0	0	0	0	0
unknown compound (217) RT1123	0	0	0	0	0	0	0	0	0	0
unknown compound (93) RT1132	0	0	0	0	0	0	0	0	0	0
unknown compound (103) RT1218	0	0	0	0	0.08	0	0	0	0	0
unknown compound (344) RT1259	0	0	0	0	0.12	0	0	0	0	0
unknown compound (179) RT1272	0	0	0	0	0	0	0	0	0	0
unknown compound (299) RT1298	0	0	0	0	4	0	0	0	0	0
unknown compound (285) RT1310	0	0	0	0	0	0	0	0	0	0
unknown compound (155) RT1366	4.5	0	0	0	0.03	0	0	0	0	0
unknown compound (185) RT1512	0	0	0	0	0	0	0	0	0	0
unknown compound (239) RT1643	0	0	0	0	0.07	0	0	0	0	0

unknown compound (83) RT1643	0	0	0	0	0	0	0	0	0	0
unknown compound (91) RT1660	0	0	0	0	0	0	0	0	0	0
unknown compound (204) RT1683	0	0	0	0	0.06	0	0	0	0	0
unknown compound (283) RT1774	0	0.12	0.05	0.18	4.67	0.07	0.11	0.13	0.17	0.09
unknown compound (204) RT1780	0	0	0	0	0.06	0	0	0	0	0
unknown compound (356) RT1864	0	0	0	0	0	0	0	0	0	0
unknown compound (217) RT1936	0	0	0	0	0.14	0	0	0	0	0
unknown compound (525) RT1992	0	0	0	0	0	0	0	0	0	0
unknown compound (361) RT2003	0	0	2*	0	0	0	0	0	0	0
unknown compound (361) RT2053	0	0	0	0	0	0	0	0	0	0
unknown compound (362) RT2071	0	0	0	0	0	0	0	0	0	0
unknown compound (204) RT2329	0	0	0	0	0	0	0	0	0	0

Supplementary Table 4: **Individual explained variance of the polar metabolite composition of exudates by single variables of the environmental factors of LNH, Soil and Climate**. Table contains the explained amount of variance (in %) of polar metabolites by different impacting factors and included variables. A detailed description of the abbreviation is given in Supplementary Table 10. Residuals = remaining unexplained variation.

	Environ-mental factor and included single variables (SV)	Species	Plot	SV	Species +Plot	Plot +SV	SV +Species	Species +Plot +SV	Residuals
forb	LNH	8.75	24.09	0	0	1.03	0	0	68.41
	- Cover	8.79	23.06	0	0	2.05	0	0	68.61
	- Richness	8.76	24.62	0	0	0.49	0	0	68.68
	- Shannon	8.76	24.62	0	0	0.49	0	0	68.68
	LUI	8.75	24.64	0	0	0.48	0	0	68.41
	- fertilization	8.75	24.18	0	0	0.93	0	0	68.41
	- grazing	8.75	24.18	0	0	0.93	0	0	68.41
	- mowing	8.53	23.74	0	0	1.37	0.22	0	68.61
	Soil	8.84	23.29	0	0	1.82	0	0.01	68.61
	- pH	8.83	23.83	0	0	1.28	0	0	68.5
	- TC	8.75	22.26	0	0	2.85	0	0	68.41
	- TN	8.75	22.65	0	0	2.46	0	0	68.42
	- moisture	8.78	24.61	0	0	0.5	0	0	68.68
	- soil cores	8.83	23.83	0	0	1.28	0	0	68.5
	- soil type	8.75	24.83	0	0	0.28	0	0	68.41
	Climate	8.75	23.38	0	0	1.73	0	0	68.41
	- precipitation	8.75	24.09	0	0	1.03	0	0	68.41
	- T(10)	8.79	23.06	0	0	2.05	0	0	68.61
	- T(200)	8.76	24.62	0	0	0.49	0	0	68.68
grass	LNH	0.74	30.72	0	0	0.65	0	0	68.78
	- Cover	0.86	24.82	0.23	0.08	6.54	0	0	68.55
	- Richness	0.69	29.51	0	0	1.85	0.05	0	68.95
	- Shannon	0.69	29.51	0	0	1.85	0.05	0	68.95
	LUI	0.74	31.47	0	0	0	0	0.03	68.78
	- fertilization	0.74	31.26	0	0	0.1	0	0.02	68.78
	- grazing	0.74	31.26	0	0	0.1	0	0.02	68.78
	- mowing	0.85	30.43	0.16	0	0.93	0	0.16	68.62
	Soil	0.73	29.92	0.09	0	1.45	0.01	0.04	68.69
	- pH	0.61	30.37	0.04	0	1	0.13	0	68.74
	- TC	0.74	23.99	0	0	7.38	0	0	68.78

- TN	0.86	24.52	0.14	0	6.85	0	0	68.64
- moisture	0.72	29.5	0	0	1.87	0.02	0	68.99
- soil cores	0.61	30.37	0.04	0	1	0.13	0	68.74
- soil type	0.74	30.25	0	0	1.11	0	0.04	68.78
Climate	0.74	24.94	0	0	6.43	0	0	68.78
- precipitation	0.74	30.72	0	0	0.65	0	0	68.78
- T(10)	0.86	24.82	0.23	0.08	6.54	0	0	68.55
- T(200)	0.69	29.51	0	0	1.85	0.05	0	68.95

Supplementary Table 5: Correlation of exuded compounds and environmental variables. Tables display the significantly correlated compound profiles of a) polar metabolite and b) semi-polar metabolite. Correlations between metabolites and variables were investigated by logistic regression in which metabolites were the dependent variable and environmental variables the independent variable. Compounds coloured in black represent all compounds correlated to the specific variable in highly significant ($p<0.001$) manner. Compounds coloured in orange represent all additional compounds being also highly significant ($p<0.01$) correlated to the specific variable. Compounds coloured in grey represent all additional compounds being significant ($p<0.05$) correlated to the specific variable. Table c) presents the percentage of correlated compounds per predictor as measured by the total number of compounds per dataset. The abbreviations are listed in Supplementary Table 10.

a) polar metabolites

	Forbs	grasses
**Cover**<br><br><u>forbs</u><br>*p<0.001* = 0<br>*p<0.01* = 5<br>*p<0.05* = 19<br>total = 24<br><br><u>grasses</u><br>*p<0.001* = 0<br>*p<0.01* = 5<br>*p<0.05* = 19<br>total = 24	compound (311) RT1362, compound (197) RT1637, compound (174) RT1212, Erythronic acid (292), compound (319) RT1466, compound (283) RT1774, compound (172) RT825, compound (223) RT983, compound (179) RT867, compound (117) RT1803, Benzoic acid (267), compound (217) RT1166, Glutamate (246), compound (207) RT454, compound (160) RT1909, compound (237) RT851, compound (158) RT944, compound (243) RT830, compound (117) RT529, compound (156) RT1226, Uracil (241), compound (57) RT1173, compound (186) RT642, compound (319) RT1487	compound (311) RT1362, compound (197) RT1637, compound (174) RT1212, Erythronic acid (292), compound (319) RT1466, compound (283) RT1774, compound (172) RT825, compound (223) RT983, compound (179) RT867, compound (117) RT1803, Benzoic acid (267), compound (217) RT1166, Glutamate (246), compound (207) RT454, compound (160) RT1909, compound (237) RT851, compound (158) RT944, compound (243) RT830, compound (117) RT529, compound (156) RT1226, Uracil (241), compound (57) RT1173, compound (186) RT642, compound (319) RT1487

**Richness**<br>forbs<br>$p<0.001$ = 23<br>$p<0.01$ = 23<br>$p<0.05$ = 35<br>total = 81	Adipic acid (111), Erythronic acid (292), compound (117) RT529, compound (324) RT1456, compound (283) RT1774, compound (361) RT1926, Leucine (158), Phenylalanine (192), Uracil (241), compound (186) RT642, compound (103) RT982, compound (223) RT983, compound (311) RT1362, compound (319) RT1466, compound (319) RT1487, compound (197) RT1637, compound (57) RT2144, compound (243) RT830, compound (217) RT1166, compound (57) RT1173, Tyrosine (218), compound (327) RT1456, Methionine (176),<br>Threonine (218), compound (117) RT1803, compound (117) RT443, compound (89) RT440, compound (132) RT1300, compound (259) RT1920, compound (217) RT1994, compound (204) RT1517, compound (172) RT825, compound (295) RT1171, Octadecenoic acid (339) , compound (204) RT1731, compound (204) RT1971, compound (263) RT932, Aspartate (232), compound (156) RT1226, compound (117) RT1037, Glutamate (246), compound (204) RT1340, compound (117) RT605, Xylitol (307), compound (292) RT1203, compound (75) RT722,<br>compound (167) RT1609, 2-Oxoglutarate (129), compound (119) RT2019, compound (281) RT548, compound (75) RT690, compound (204) RT1603, compound (191) RT2175, compound (158) RT944, fatty acid (339) RT1518, beta-Alanine (248), carbohydrate (204) RT1781, compound (142) RT973, Gluconate (333), compound (333) RT1325, compound (259) RT1657, Adenosine (236), compound (335) RT1385, compound (361) RT1919, compound (160) RT1319, compound (204) RT1756, compound (273) RT1297, compound (306) RT1865, compound (205) RT653, compound (255) RT1654, compound (319) RT1316, compound (285) RT1213, compound (204) RT1798, compound (69) RT1140, compound (57) RT1127, compound (179) RT867, Shikimate (204), compound (260) RT1676, compound (327) RT2065, compound (160) RT1909, compound (117) RT459	Adipic acid (111), Erythronic acid (292), compound (117) RT529, compound (324) RT1456, compound (283) RT1774, compound (361) RT1926, Leucine (158), Phenylalanine (192), Uracil (241), compound (186) RT642, compound (103) RT982, compound (223) RT983, compound (311) RT1362, compound (319) RT1466, compound (319) RT1487, compound (197) RT1637, compound (57) RT2144, compound (243) RT830, compound (217) RT1166, compound (57) RT1173, Tyrosine (218), compound (327) RT1456, Methionine (176),<br>Threonine (218), compound (117) RT1803, compound (117) RT443, compound (89) RT440, compound (132) RT1300, compound (259) RT1920, compound (217) RT1994, compound (204) RT1517, compound (172) RT825, compound (295) RT1171, Octadecenoic acid (339) , compound (204) RT1731, compound (204) RT1971, compound (263) RT932, Aspartate (232), compound (156) RT1226, compound (117) RT1037, Glutamate (246), compound (204) RT1340, compound (117) RT605, Xylitol (307), compound (292) RT1203, compound (75) RT722,<br>compound (167) RT1609, 2-Oxoglutarate (129), compound (119) RT2019, compound (281) RT548, compound (75) RT690, compound (204) RT1603, compound (191) RT2175, compound (158) RT944, fatty acid (339) RT1518, compound (77) RT466, beta-Alanine (248), carbohydrate (204) RT1781, compound (142) RT973, Gluconate (333), compound (333) RT1325, compound (259) RT1657, Adenosine (236), compound (335) RT1385, compound (361) RT1919, compound (160) RT1319, compound (204) RT1756, compound (273) RT1297, compound (306) RT1865, compound (205) RT653, compound (255) RT1654, compound (319) RT1316, compound (285) RT1213, compound (204) RT1798, compound (69) RT1140, compound (57) RT1127, compound (179) RT867, Shikimate (204), compound (260) RT1676, compound (327) RT2065, compound (160) RT1909, compound (117) RT459
grasses<br>$p<0.001$ = 23<br>$p<0.01$ = 23<br>$p<0.05$ = 36<br>total = 82		

**Shannon**<br>forbs<br>*p*<0.001 = 22<br>*p*<0.01 = 22<br>*p*<0.05 = 34<br>total = 78	Uracil (241), compound (117) RT529, compound (217) RT1994, Adipic acid (111), Leucine (158), Phenylalanine (192), Threonine (218), compound (186) RT642, compound (103) RT982, compound (217) RT1166, compound (57) RT1173, compound (311) RT1362, compound (324) RT1456, compound (319) RT1466, compound (319) RT1487, compound (57) RT2144, compound (197) RT1637, compound (283) RT1774, compound (259) RT1920, compound (191) RT2175, compound (259) RT1657, compound (318) RT1334,<br>compound (223) RT983, compound (191) RT1825, Erythronic acid (292), compound (361) RT1926, compound (75) RT690, compound (292) RT1203, Xylitol (307), compound (327) RT1456, Tyrosine (218), compound (255) RT1654, Methionine (176), compound (167) RT1609, compound (204) RT1340, Gluconate (333), carbohydrate (204) RT1781, 2-Oxoglutarate (129), fatty acid (339) RT1518, compound (263) RT932, Aspartate (232), compound (75) RT722, Benzoic acid (267), compound (243) RT830,<br>compound (204) RT1971, compound (204) RT1731, compound (117) RT1803, compound (119) RT2019, compound (204) RT1517, compound (361) RT1948, compound (158) RT533, compound (204) RT1603, compound (361) RT1919, compound (333) RT1325, compound (319) RT1316, compound (285) RT1213, Octadecenoic acid (339) , compound (173) RT1809, compound (158) RT944, compound (281) RT548, compound (362) RT1978, compound (319) RT1968, compound (69) RT1140, compound (156) RT1226, compound (295) RT1171, Glutamate (246), compound (273) RT1297, compound (142) RT973, compound (306) RT1865, compound (132) RT1300, compound (217) RT964, compound (204) RT1655, compound (207) RT436, Adenosine (236), compound (172) RT825, compound (160) RT1319, compound (149) RT1233, compound (261) RT1791	Uracil (241), compound (117) RT529, compound (217) RT1994, Adipic acid (111), Leucine (158), Phenylalanine (192), Threonine (218), compound (186) RT642, compound (103) RT982, compound (217) RT1166, compound (57) RT1173, compound (311) RT1362, compound (324) RT1456, compound (319) RT1466, compound (319) RT1487, compound (57) RT2144, compound (197) RT1637, compound (283) RT1774, compound (259) RT1920, compound (191) RT2175, compound (259) RT1657, compou11nd (318) RT1334,<br>compound (223) RT983, Erythronic acid (292), compound (361) RT1926, compound (75) RT690, compound (292) RT1203, Xylitol (307), compound (327) RT1456, Tyrosine (218), compound (255) RT1654, Methionine (176), compound (167) RT1609, compound (204) RT1340, Gluconate (333), carbohydrate (204) RT1781, 2-Oxoglutarate (129), fatty acid (339) RT1518, compound (263) RT932, Aspartate (232), compound (75) RT722, Benzoic acid (267), compound (243) RT830, compound (191) RT1825,<br>compound (204) RT1971, compound (204) RT1731, compound (117) RT1803, compound (119) RT2019, compound (217) RT1155, compound (204) RT1517, compound (361) RT1948, compound (158) RT533, compound (204) RT1603, compound (361) RT1919, compound (333) RT1325, compound (319) RT1316, compound (285) RT1213, Octadecenoic acid (339) , compound (173) RT1809, compound (158) RT944, compound (281) RT548, compound (362) RT1978, compound (319) RT1968, compound (69) RT1140, compound (156) RT1226, compound (295) RT1171, compound (77) RT466, Glutamate (246), compound (273) RT1297, compound (142) RT973, compound (306) RT1865, compound (132) RT1300, compound (217) RT964, compound (204) RT1655, compound (207) RT436, Adenosine (236), compound (172) RT825, compound (160) RT1319, compound (149) RT1233, compound (261) RT1791

**Fertilization**<br>forbs<br>$p<0.001$ = 0<br>$p<0.01$ = 1<br>$p<0.05$ = 8<br>total = 9<br><br>grasses<br>$p<0.001$ = 1<br>$p<0.01$ = 7<br>$p<0.05$ = 8<br>total = 41	carbohydrate (319) RT1314,<br>compound (117) RT1121, compound (75) RT824, compound (204) RT2068, compound (71) RT1316, Tyramine (174), compound (127) RT1780, compound (160) RT1909, Adenosine (236)	carbohydrate (319) RT1314,<br>compound (117) RT1121, compound (75) RT824, compound (71) RT1316, Tyramine (174), compound (127) RT1780, compound (160) RT1909, Adenosine (236)
**Grazing**<br>forbs<br>$p<0.001$ = 6<br>$p<0.01$ = 15<br>$p<0.05$ = 20<br>total = 41<br><br>grasses<br>$p<0.001$ = 6<br>$p<0.01$ = 14<br>$p<0.05$ = 34<br>total = 20	compound (75) RT1218, compound (160) RT1240, compound (333) RT1325, Methionine (176), Phenylalanine (192), Phosphoenolpyruvate (247),<br>Asparagine (231), compound (280) RT669, compound (223) RT983, compound (335) RT1385, compound (217) RT1994, compound (204) RT1340, compound (179) RT621, compound (319) RT1466, compound (362) RT1978, compound (361) RT1919, Uracil (241), compound (69) RT1140, Shikimate (204), compound (149) RT1233, compound (204) RT1798,<br>compound (207) RT436, Benzoic acid (267), Tyrosine (218), compound (57) RT1655, compound (204) RT2011, Glutamate (246), Aspartate (232), compound (319) RT1968, compound (103) RT1133, compound (361) RT1948, compound (217) RT964, compound (255) RT1654, compound (285) RT1213, compound (319) RT1316, compound (169) RT581, compound (205) RT653, compound (156) RT1226, Gluconate (333), compound (292) RT1203, Homoserine (218)	compound (75) RT1218, compound (160) RT1240, compound (333) RT1325, Phosphoenolpyruvate (247), Methionine (176), Phenylalanine (192),<br>compound (280) RT669, compound (223) RT983, compound (335) RT1385, compound (179) RT621, compound (204) RT1340, compound (217) RT1994, compound (319) RT1466, compound (362) RT1978, compound (361) RT1919, Uracil (241), Shikimate (204), compound (69) RT1140, compound (149) RT1233, compound (204) RT1798,<br>compound (207) RT436, Benzoic acid (267), Tyrosine (218), compound (57) RT1655, compound (204) RT2011, Glutamate (246), Aspartate (232), compound (319) RT1968, compound (103) RT1133, compound (217) RT964, compound (361) RT1948, compound (255) RT1654, compound (285) RT1213, compound (319) RT1316, compound (169) RT581, compound (205) RT653, compound (156) RT1226, Gluconate (333), compound (292) RT1203, Homoserine (218)

**Mowing**<br>forbs<br>$p<0.001$ = 0<br>$p<0.01$ = 3<br>$p<0.05$ = 4<br>total = 7<br><br>grasses<br>$p<0.001$ = 0<br>$p<0.01$ = 3<br>$p<0.05$ = 19<br>total = 22	Proline (142), Tyramine (174), Isoleucine (158),<br>compound (75) RT824, Salicylic acid (267), Serine (204), 5-Indolecarboxylic acid (305)	Proline (142), Tyramine (174), Isoleucine (158),<br>compound (174) RT433, compound (75) RT435, compound (112) RT451, compound (127) RT470, compound (58) RT470, compound (173) RT665, compound (245) RT1065, compound (103) RT1218, compound (285) RT1310, compound (239) RT1643, compound (83) RT1643, compound (91) RT1660, compound (204) RT1683, compound (204) RT1780, compound (204) RT2329, compound (75) RT824, Salicylic acid (267), Serine (204), 5-Indolecarboxylic acid (305)
**pH**<br>forbs<br>$p<0.001$ = 3<br>$p<0.01$ = 7<br>$p<0.05$ = 9<br>total = 19<br><br>grasses<br>$p<0.001$ = 3<br>$p<0.01$ = 7<br>$p<0.05$ = 9<br>total = 19	compound (261) RT1791, compound (174) RT924, compound (197) RT1637,<br>compound (149) RT1745, compound (132) RT1300, compound (295) RT1171, compound (69) RT1140, Glycerol 3-phosphate (357), compound (172) RT825, compound (204) RT1731,<br>compound (319) RT1487, compound (117) RT1803, compound (173) RT1809, compound (219) RT1776, compound (204) RT1655, Adipic acid (111), compound (186) RT642, compound (237) RT851, Octadecenoic acid (339)	compound (174) RT924, compound (197) RT1637, compound (261) RT1791,<br>compound (149) RT1745, compound (132) RT1300, compound (295) RT1171, compound (69) RT1140, Glycerol 3-phosphate (357), compound (172) RT825, compound (204) RT1731,<br>compound (319) RT1487, compound (117) RT1803, compound (173) RT1809, compound (219) RT1776, compound (204) RT1655, Adipic acid (111), compound (186) RT642, compound (237) RT851, Octadecenoic acid (339)
**moisture**<br>forbs<br>$p<0.001$ = 35<br>$p<0.01$ = 38<br>$p<0.05$ = 28<br>total = 101<br><br>grasses<br>$p<0.001$ = 34<br>$p<0.01$ = 38<br>$p<0.05$ = 29<br>total = 101	Adipic acid (111), Alanine (116), Leucine (158), Octadecenoic acid (339) , Threonine (218), Uracil (241), compound (319) RT1316, compound (204) RT1340, Aspartate (232), Glutamate (246), Glycerol 3-phosphate (357), Homoserine (218), Methionine (176), compound (280) RT669, compound (223) RT983, compound (217) RT1166, compound (57) RT1173, compound (167) RT1609, compound (283) RT1774, compound (204) RT1798, compound (361) RT1948, compound (57) RT2144, compound (204) RT2147, compound (75) RT722, compound (69) RT1140, compound (149) RT1233, compound (335) RT1385, compound (204) RT1603, fatty acid (339) RT1518, compound (281) RT548, compound (103) RT803,	Adipic acid (111), Alanine (116), Leucine (158), Octadecenoic acid (339) , Threonine (218), Uracil (241), compound (319) RT1316, compound (204) RT1340, Aspartate (232), Glutamate (246), Glycerol 3-phosphate (357), Homoserine (218), Methionine (176), compound (280) RT669, compound (223) RT983, compound (217) RT1166, compound (57) RT1173, compound (167) RT1609, compound (283) RT1774, compound (204) RT1798, compound (361) RT1948, compound (57) RT2144, compound (75) RT722, compound (69) RT1140, compound (149) RT1233, compound (335) RT1385, compound (204) RT1603, fatty acid (339) RT1518,

compound (243) RT830, compound (117) RT529, compound (205) RT653, compound (204) RT1615, compound (174) RT1143, compound (191) RT2175, Glucose-6-phosphate (387), Myo-Inositol-1-phosphate (318), Tyrosine (218), compound (158) RT533, compound (361) RT1919, 2-Aminoadipate (260), compound (207) RT436, compound (179) RT867, compound (273) RT1297, compound (174) RT1327, compound (103) RT982, Phosphoenolpyruvate (247), Phenylalanine (192), compound (311) RT1362, Rhamnose (117), compound (75) RT690, compound (217) RT964, compound (319) RT1466, cis-Aconitate (229), compound (260) RT1676, Tryptophan (202), compound (103) RT1243, compound (204) RT1731, compound (204) RT1517, compound (319) RT1968, compound (217) RT1180, Tartaric acid (292), compound (119) RT2019, compound (259) RT1920, Adenine (264), compound (217) RT2026, compound (217) RT1994, compound (204) RT2011, compound (117) RT1803, compound (299) RT1309, Gluconate (333), compound (89) RT575, Xylitol (307), compound (204) RT1971, compound (117) RT1037, Salicylic acid (267), Serine (204), compound (142) RT973, compound (295) RT1259, compound (361) RT1330, compound (75) RT1218, compound (103) RT1133, compound (333) RT1325, Ornithine / Citrullin (142), compound (255) RT1654, compound (156) RT1226, Sorbitol (217), compound (69) RT1150, compound (362) RT1978, compound (149) RT1745, compound (292) RT1203, compound (217) RT1877, Valine (144), compound (172) RT825, scyllo-inositol (204) , compound (261) RT1791, compound (160) RT1319, compound (217) RT1443, compound (160) RT1240

compound (281) RT548, compound (103) RT803, compound (243) RT830, compound (117) RT529, compound (205) RT653, compound (204) RT1615, compound (191) RT2175, compound (174) RT1143, Glucose-6-phosphate (387), compound (204) RT2011, compound (117) RT1803, compound (299) RT1309, Gluconate (333), Myo-Inositol-1-phosphate (318), Tyrosine (218), compound (158) RT533, compound (361) RT1919, 2-Aminoadipate (260), compound (207) RT436, compound (179) RT867, compound (273) RT1297, compound (174) RT1327, compound (103) RT982, Phosphoenolpyruvate (247), Phenylalanine (192), compound (311) RT1362, Rhamnose (117), compound (75) RT690, compound (217) RT964, compound (319) RT1466, cis-Aconitate (229), compound (260) RT1676, Tryptophan (202), compound (103) RT1243, compound (204) RT1731, compound (204) RT1517, compound (319) RT1968, compound (217) RT1180, Tartaric acid (292), compound (119) RT2019, compound (259) RT1920, Adenine (264), compound (217) RT2026, compound (217) RT1994, compound (89) RT575, Xylitol (307), compound (204) RT1971, compound (117) RT1037, Salicylic acid (267), Serine (204), compound (142) RT973, compound (295) RT1259, compound (361) RT1330, compound (75) RT1218, compound (103) RT1133, compound (333) RT1325, Ornithine / Citrullin (142), compound (255) RT1654, compound (156) RT1226, Aminomalonic acid (218) , Sorbitol (217), compound (69) RT1150, compound (362) RT1978, compound (149) RT1745, compound (292) RT1203, compound (217) RT1877, Valine (144), compound (172) RT825, scyllo-inositol (204) , compound (261) RT1791, compound (160) RT1319, compound (217) RT1443, compound (160) RT1240

**soil texture**<br>forbs<br>$p<0.001$ = 4<br>$p<0.01$ = 17<br>$p<0.05$ = 11<br>total = 32<br><br>grasses<br>$p<0.001$ = 4<br>$p<0.01$ = 16<br>$p<0.05$ = 12<br>total = 32	Leucine (158), compound (89) RT440, compound (91) RT2097, 2-Isopropylmalate (275),<br>compound (204) RT1958, compound (158) RT533, Xylose (217), compound (103) RT1055, compound (204) RT1834, compound (89) RT439, compound (158) RT944, Melibiose (361), compound (117) RT1803, compound (223) RT1937, compound (281) RT548, 4-Aminobutanoate [GABA] (174), compound (204) RT1749, compound (204) RT1655, compound (319) RT1487, compound (362) RT2071, compound (117) RT529,<br>compound (342) RT996, Lactic acid (191), compound (197) RT1637, Adipic acid (111), compound (75) RT824, Benzoic acid (267), compound (261) RT1791, compound (204) RT1939, Lactose (361), compound (132) RT1300, compound (273) RT1297	Leucine (158), compound (89) RT440, compound (91) RT2097, 2-Isopropylmalate (275),<br>compound (204) RT1958, compound (158) RT533, Xylose (217), compound (103) RT1055, compound (204) RT1834, compound (89) RT439, compound (158) RT944, Melibiose (361), compound (117) RT1803, compound (223) RT1937, compound (281) RT548, 4-Aminobutanoate [GABA] (174), compound (204) RT1749, compound (204) RT1655, compound (319) RT1487, compound (117) RT529,<br>compound (342) RT996, Lactic acid (191), compound (197) RT1637, Adipic acid (111), compound (75) RT824, Benzoic acid (267), compound (261) RT1791, compound (204) RT1939, Lactose (361), compound (132) RT1300, compound (217) RT1155, compound (273) RT1297

**soil type**<br>Forbs<br>*p<0.001* = 2<br>*p<0.01* = 3<br>*p<0.05* = 7<br>total = 12<br><br>Grasses<br>*p<0.001* = 1<br>*p<0.01* = 3<br>*p<0.05* = 7<br>total = 11	compound (89) RT440, compound (216) RT1788,<br>compound (160) RT1909, 2-Isopropylmalate (275), compound (89) RT439,<br>compound (91) RT2097, compound (204) RT2011, 4-Aminobutanoate [GABA] (174), compound (103) RT1264, compound (204) RT1902, Phosphoenolpyruvate (247), compound (342) RT996	compound (89) RT440,<br>compound (160) RT1909, 2-Isopropylmalate (275), compound (89) RT439,<br>compound (91) RT2097, compound (204) RT2011, 4-Aminobutanoate [GABA] (174), compound (103) RT1264, compound (204) RT1902, Phosphoenolpyruvate (247), compound (342) RT996
**TC**<br>forbs<br>*p<0.001* = 6<br>*p<0.01* = 7<br>*p<0.05* = 16<br>total = 29<br><br>grasses<br>*p<0.001* = 0<br>*p<0.01* = 6<br>*p<0.05* = 7<br>total = 15	compound (120) RT940, compound (295) RT1171, Glycerol 3-phosphate (357), compound (158) RT944, compound (324) RT1456, compound (207) RT436,<br>compound (191) RT1825, compound (273) RT1289, Myo-Inositol-1-phosphate (318), compound (319) RT1487, compound (263) RT932, compound (299) RT1309, compound (103) RT982,<br>2-Oxoglutarate (129), compound (361) RT1909, compound (132) RT1300, compound (204) RT2068, compound (281) RT610, compound (172) RT825, compound (160) RT1319, compound (327) RT1456, carbohydrate (319) RT1321, Tartaric acid (292), compound (204) RT2011, compound (297) RT2022, compound (204) RT1731, compound (174) RT924, compound (292) RT1203, compound (204) RT1655	compound (120) RT940, compound (295) RT1171, Glycerol 3-phosphate (357), compound (158) RT944, compound (324) RT1456, compound (207) RT436,<br>compound (191) RT1825, compound (273) RT1289, Myo-Inositol-1-phosphate (318), compound (319) RT1487, compound (263) RT932, compound (299) RT1309, compound (103) RT982,<br>2-Oxoglutarate (129), compound (361) RT1909, compound (132) RT1300, compound (281) RT610, compound (172) RT825, compound (160) RT1319, compound (327) RT1456, carbohydrate (319) RT1321, Tartaric acid (292), compound (204) RT2011, compound (297) RT2022, compound (204) RT1731, compound (174) RT924, compound (292) RT1203, compound (204) RT1655
**TN**<br>forbs<br>*p<0.001* = 3<br>*p<0.01* = 9<br>*p<0.05* = 13<br>total = 25	compound (207) RT436, compound (117) RT605, compound (324) RT1456,<br>compound (295) RT1171, compound (158) RT944, Glycerol 3-phosphate (357), compound (120) RT940, 2-Oxoglutarate (129), Myo-Inositol-1-phosphate (318), compound (299) RT1309, compound (319) RT1487, compound (204) RT2068,	compound (207) RT436, compound (117) RT605, compound (324) RT1456,<br>compound (295) RT1171, compound (158) RT944, Glycerol 3-phosphate (357), compound (120) RT940, 2-Oxoglutarate (129), Myo-Inositol-1-phosphate (318), compound (299) RT1309, compound (319) RT1487,

<u>grasses</u><br>$p<0.001$ = 3<br>$p<0.01$ = 8<br>$p<0.05$ = 13<br>total = 24	compound (273) RT1289, compound (361) RT1909, compound (132) RT1300, compound (197) RT1637, compound (263) RT932, compound (117) RT1803, compound (191) RT1825, carbohydrate (319) RT1321, compound (172) RT825, compound (297) RT2022, compound (318) RT1334, compound (281) RT610, Tyramine (174)	compound (273) RT1289, compound (361) RT1909, compound (132) RT1300, compound (197) RT1637, compound (263) RT932, compound (117) RT1803, compound (191) RT1825, carbohydrate (319) RT1321, compound (172) RT825, compound (297) RT2022, compound (318) RT1334, compound (281) RT610, Tyramine (174)
**Precipitation**<br><u>forbs</u><br>$p<0.001$ = 0<br>$p<0.01$ = 0<br>$p<0.05$ = 7<br>total = 7	compound (223) RT983, compound (204) RT1749, Glycerol 3-phosphate (357), compound (156) RT1226, compound (285) RT1213, Octadecenoic acid (339) , compound (87) RT435	Succinate (147),<br>Glycerol 3-phosphate (357), compound (223) RT983, compound (204) RT1749, compound (156) RT1226, compound (285) RT1213, Octadecenoic acid (339) , compound (87) RT435
<u>grasses</u><br>$p<0.001$ = 0<br>$p<0.01$ = 1<br>$p<0.05$ = 7<br>total = 8		
**T(10)**<br><u>forbs</u><br>$p<0.001$ = 0<br>$p<0.01$ = 3<br>$p<0.05$ = 10<br>total = 13	compound (204) RT2011, compound (159) RT650, compound (205) RT653,<br>Tyrosine (218), carbohydrate (319) RT1321, compound (227) RT945, compound (281) RT548, compound (201) RT1994, Methionine (176), Leucine (158), compound (297) RT2022, Pinitol (260) , compound (327) RT2065	compound (204) RT2011, compound (159) RT650, compound (205) RT653,<br>Tyrosine (218), carbohydrate (319) RT1321, compound (227) RT945, compound (281) RT548, compound (201) RT1994, Methionine (176), Leucine (158), compound (297) RT2022, Pinitol (260) , compound (327) RT2065
<u>grasses</u><br>$p<0.001$ = 0<br>$p<0.01$ = 3<br>$p<0.05$ = 10<br>total = 13		

**T(200)**<br>forbs<br>$p<0.001$ = 20<br>$p<0.01$ = 31<br>$p<0.05$ = 28<br>total = 79<br><br>grasses<br>$p<0.001$ = 21<br>$p<0.01$ = 29<br>$p<0.05$ = 28<br>total = 78	Adipic acid (111), compound (117) RT443, compound (281) RT548, compound (243) RT830, compound (273) RT1297, compound (204) RT1731, Leucine (158), compound (142) RT973, compound (283) RT1774, compound (57) RT2144, compound (327) RT2065, compound (204) RT1615, compound (103) RT982, compound (319) RT1487, Erythronic acid (292), compound (205) RT653, compound (117) RT1803, compound (75) RT722, compound (186) RT642, compound (117) RT529, Asparagine (231), Aspartate (232), Tryptophan (202), compound (280) RT669, compound (103) RT803, compound (361) RT1926, Methionine (176), compound (204) RT1603, Tyrosine (218), Uracil (241), Threonine (218), compound (204) RT1971, compound (103) RT1264, compound (174) RT1327, compound (217) RT1166, compound (167) RT1609, compound (260) RT1676, compound (179) RT867, compound (149) RT1745, Glutamate (246), compound (119) RT2019, compound (158) RT533, Glycerol 3-phosphate (357), compound (197) RT1637, compound (204) RT2011, compound (204) RT1796, scyllo-inositol (204) , compound (69) RT1150, compound (299) RT1309, compound (191) RT2175, Phenylalanine (192), compound (57) RT1127, compound (179) RT621, beta-Alanine (248), compound (311) RT1362, compound (227) RT945, compound (57) RT1173, Ornithine / Citrullin (142), compound (69) RT1140, compound (204) RT1517, Octadecenoic acid (339) , compound (223) RT983, Homoserine (218), compound (117) RT1037, compound (204) RT1340, fatty acid (339) RT1518, compound (156) RT1226, Pinitol (260) , compound (292) RT1203, compound (91) RT2097, Adenine (264), compound (319) RT1968, compound (259) RT1920, compound (172) RT825, compound (241) RT553, compound (217) RT964, compound (319) RT1466, Rhamnose (117), compound (355) RT515	Adipic acid (111), compound (117) RT443, compound (77) RT466, compound (281) RT548, compound (243) RT830, compound (273) RT1297, compound (204) RT1731, Leucine (158), compound (142) RT973, compound (283) RT1774, compound (57) RT2144, compound (327) RT2065, compound (204) RT1615, compound (103) RT982, compound (319) RT1487, Erythronic acid (292), compound (205) RT653, compound (117) RT1803, compound (75) RT722, compound (186) RT642, compound (117) RT529, Aspartate (232), Tryptophan (202), compound (280) RT669, compound (103) RT803, compound (361) RT1926, Methionine (176), compound (204) RT1603, Tyrosine (218), Uracil (241), Threonine (218), compound (204) RT1971, compound (103) RT1264, compound (174) RT1327, compound (217) RT1166, compound (167) RT1609, compound (260) RT1676, compound (179) RT867, compound (149) RT1745, Glutamate (246), compound (119) RT2019, compound (158) RT533, Glycerol 3-phosphate (357), compound (197) RT1637, compound (204) RT2011, scyllo-inositol (204) , compound (69) RT1150, compound (299) RT1309, compound (191) RT2175, Phenylalanine (192), compound (57) RT1127, compound (179) RT621, beta-Alanine (248), compound (311) RT1362, compound (227) RT945, compound (57) RT1173, Ornithine / Citrullin (142), compound (69) RT1140, compound (204) RT1517, Octadecenoic acid (339) , compound (223) RT983, Homoserine (218), compound (117) RT1037, compound (204) RT1340, fatty acid (339) RT1518, compound (156) RT1226, Pinitol (260) , compound (292) RT1203, compound (91) RT2097, Adenine (264), compound (319) RT1968, compound (259) RT1920, compound (172) RT825, compound (241) RT553, compound (217) RT964, compound (319) RT1466, Rhamnose (117), compound (355) RT515

b) semi-polar metabolites

	Forbs	Grasses
**Cover**<br>forbs<br>$p<0.001$ = 26<br>$p<0.01$ = 33<br>$p<0.05$ = 47<br>total = 106<br><br>grasses<br>$p<0.001$ = 14<br>$p<0.01$ = 12<br>$p<0.05$ = 37<br>total = 63	445.1907m/z_3.28min, 291.0874m/z_3.49min,<br>579.1308m/z_3.64min, 261.0434m/z_3.7min,<br>623.1629m/z_3.92min, 385.0547m/z_3.97min,<br>475.1722m/z_4.08min, 615.2199m/z_4.54min,<br>200.9959m/z_4.77min, 633.2376m/z_4.8min,<br>441.1079m/z_5.08min, 443.2276m/z_5.16min,<br>366.0968m/z_5.33min, 351.0115m/z_6.24min,<br>272.0338m/z_8.4min, 337.2032m/z_9.3min,<br>511.1454m/z_4.08min, 279.2323m/z_13.23min,<br>461.1306m/z_4.02min, 393.1711m/z_11.42min,<br>509.0895m/z_2.04min, 259.1079m/z_3.96min,<br>737.1919m/z_4.17min, 821.2118m/z_3.6min,<br>165.0184m/z_3.01min, 219.0869m/z_3.63min,<br>377.1792m/z_2.89min, 591.1649m/z_3.07min,<br>319.0859m/z_3.13min, 275.0856m/z_3.51min,<br>535.2379m/z_3.59min, 493.1943m/z_3.61min,<br>445.1185m/z_3.86min, 455.1035m/z_4.11min,<br>635.1613m/z_4.38min, 345.1539m/z_4.68min,<br>489.197m/z_5.48min, 517.2283m/z_5.85min,<br>259.0824m/z_6.02min, 445.2418m/z_6.06min,<br>257.1397m/z_6.29min, 401.124m/z_6.55min,<br>509.2225m/z_2.72min, 295.0134m/z_6min,<br>303.1513m/z_6.42min, 547.2386m/z_4.38min,<br>389.1117m/z_3.09min, 267.0871m/z_3.62min,<br>469.1666m/z_4.27min, 365.1495m/z_9.47min,<br>329.2324m/z_7.49min, 339.1991m/z_11.9min,<br>671.291m/z_6.56min, 379.2096m/z_9.07min,<br>357.2133m/z_1.3min, 456.2459m/z_2.74min,<br>281.14m/z_8.95min, 288.9479m/z_7.1min,<br>311.2219m/z_7.72min,<br>283.0368m/z_5.63min, 209.0792m/z_4.9min, 403.1956m/z_4.2min,<br>611.1828m/z_2.64min, 599.1941m/z_8.39min, 295.2269m/z_10.1min,	623.1629m/z_3.92min, 549.3402m/z_5.25min,<br>681.3819m/z_5.25min, 461.2202m/z_5.99min, 813.428m/z_5min,<br>377.2265m/z_6.45min, 689.4298m/z_6.88min,<br>329.2316m/z_7.29min, 325.1998m/z_7.78min,<br>337.2032m/z_9.3min, 279.2323m/z_13.23min,<br>393.1711m/z_11.42min, 229.0774m/z_5.35min,<br>533.3115m/z_6.79min,<br>303.1513m/z_6.42min, 547.2386m/z_4.38min,<br>267.0871m/z_3.62min, 469.1666m/z_4.27min,<br>365.1495m/z_9.47min, 329.2324m/z_7.49min,<br>671.291m/z_6.56min, 379.2096m/z_9.07min,<br>365.2304m/z_10.77min, 357.2133m/z_1.3min,<br>288.9479m/z_7.1min, 311.2219m/z_7.72min,<br>209.0792m/z_4.9min, 403.1956m/z_4.2min, 295.2269m/z_10.1min,<br>293.2111m/z_10.79min, 429.1762m/z_4.96min,<br>273.1701m/z_7.68min, 283.0368m/z_5.63min,<br>193.0504m/z_3.75min, 200.9961m/z_4.08min,<br>295.227m/z_10.35min, 293.2109m/z_9.69min,<br>327.2111m/z_7.58min, 242.1757m/z_6.64min,<br>355.2085m/z_8.17min, 239.1284m/z_4.91min,<br>339.1993m/z_12.63min, 227.2013m/z_12.53min,<br>580.965m/z_7.42min, 343.0515m/z_4.34min,<br>389.1087m/z_1.15min, 480.9715m/z_6.3min,<br>381.2311m/z_13.44min, 329.2324m/z_6.83min,<br>201.1129m/z_5.67min, 293.2111m/z_9.42min,<br>361.1622m/z_8.15min, 379.1578m/z_10.2min,<br>431.1703m/z_8.05min, 287.2221m/z_11.9min,<br>438.9672m/z_4.47min, 327.2169m/z_8.34min,<br>297.1522m/z_9.69min, 339.1995m/z_12.42min,<br>440.9281m/z_7.05min, 467.0597m/z_3.97min,<br>327.2168m/z_6.47min, 593.1416m/z_3.6min

	293.2111m/z_10.79min, 429.1762m/z_4.96min,<br>381.0951m/z_1.08min, 273.1701m/z_7.68min, 193.0504m/z_3.75min,<br>237.0557m/z_8.07min, 493.1749m/z_4.4min, 200.9961m/z_4.08min,<br>295.227m/z_10.35min, 293.2109m/z_9.69min, 283.0461m/z_6.61min,<br>327.2111m/z_7.58min, 242.1757m/z_6.64min, 355.2085m/z_8.17min,<br>239.1284m/z_4.91min, 339.1993m/z_12.63min,<br>227.2013m/z_12.53min, 580.965m/z_7.42min, 343.0515m/z_4.34min,<br>389.1087m/z_1.15min, 475.0883m/z_4.96min, 480.9715m/z_6.3min,<br>381.2311m/z_13.44min, 329.2324m/z_6.83min,<br>201.1129m/z_5.67min, 465.1179m/z_2.92min, 293.2111m/z_9.42min,<br>525.2182m/z_2.76min, 361.1622m/z_8.15min, 379.1578m/z_10.2min,<br>585.1615m/z_4.85min, 431.1703m/z_8.05min, 287.2221m/z_11.9min,<br>438.9672m/z_4.47min, 327.2169m/z_8.34min, 297.1522m/z_9.69min,<br>339.1995m/z_12.42min, 440.9281m/z_7.05min,<br>467.0597m/z_3.97min, 327.2168m/z_6.47min, 593.1416m/z_3.6min	
**Richness**<br><u>forbs</u><br>*p<0.001* = 18<br>*p<0.01* = 8<br>*p<0.05* = 30<br>total = 56<br><br><u>grasses</u><br>*p<0.001* = 18<br>*p<0.01* = 4<br>*p<0.05* = 30<br>total = 52	537.1584m/z_4.22min, 137.0244m/z_2.74min,<br>637.1419m/z_3.67min, 821.2118m/z_3.6min,<br>664.1887m/z_3.76min, 525.1612m/z_3.78min,<br>678.2013m/z_3.81min, 379.0093m/z_4.17min,<br>737.1919m/z_4.17min, 547.2386m/z_4.38min,<br>581.1881m/z_4.72min, 827.3337m/z_4.91min,<br>567.3263m/z_5.22min, 431.1703m/z_8.05min,<br>347.243m/z_8.19min, 461.1318m/z_2.82min,<br>239.1284m/z_4.91min, 267.0871m/z_3.62min,<br>401.1081m/z_2.92min, 253.0503m/z_7.02min,<br>389.1117m/z_3.09min, 397.0925m/z_8.24min,<br>297.0398m/z_7.29min, 611.185m/z_5.05min,<br>597.2157m/z_5.97min, 463.1626m/z_6.08min,<br>651.2143m/z_3.82min, 129.0553m/z_2.75min, 507.2356m/z_6.17min,<br>285.2064m/z_7.01min, 231.0301m/z_7.39min, 255.0302m/z_8.01min,<br>353.1941m/z_7.01min, 221.1538m/z_8.15min, 277.067m/z_4.92min,<br>403.1874m/z_4.13min, 329.2324m/z_6.83min, 369.0952m/z_8.75min,<br>269.0851m/z_9.66min, 429.1377m/z_3.26min, 467.1615m/z_3.73min,<br>267.0304m/z_9.64min, 449.1299m/z_2.3min, 469.1666m/z_4.27min,<br>242.1757m/z_6.64min, 653.2065m/z_6.17min, 627.1571m/z_5.51min,<br>299.0213m/z_5.23min, 493.1756m/z_4.14min, 589.0386m/z_6.18min,<br>507.2076m/z_3.72min, 339.1993m/z_12.63min,	637.1419m/z_3.67min, 664.1887m/z_3.76min,<br>678.2013m/z_3.81min, 547.2386m/z_4.38min,<br>607.1664m/z_4.67min, 699.3939m/z_4.6min,<br>815.4389m/z_4.83min, 813.4231m/z_5.11min,<br>567.3263m/z_5.22min, 329.2316m/z_7.29min,<br>431.1703m/z_8.05min, 537.1584m/z_4.22min,<br>359.2433m/z_7.84min, 448.1236m/z_5.1min,<br>239.1284m/z_4.91min, 661.3633m/z_6.01min,<br>465.2486m/z_4.88min, 267.0871m/z_3.62min,<br>269.0632m/z_4.06min, 221.0812m/z_7.35min,<br>588.2609m/z_6.15min, 611.185m/z_5.05min,<br>651.2143m/z_3.82min, 129.0553m/z_2.75min,<br>285.2064m/z_7.01min, 353.1941m/z_7.01min,<br>221.1538m/z_8.15min, 277.067m/z_4.92min,<br>329.2324m/z_6.83min, 429.1377m/z_3.26min,<br>467.1615m/z_3.73min, 451.1952m/z_4.57min,<br>553.3379m/z_4.68min, 231.1216m/z_4.76min,<br>659.3782m/z_4.89min, 315.0521m/z_4.97min,<br>381.0967m/z_5.34min, 617.2243m/z_5.73min, 297.04m/z_6.95min,<br>521.3466m/z_7.03min, 649.3931m/z_7.35min,<br>295.0239m/z_8.07min, 305.1741m/z_8.2min,<br>469.1666m/z_4.27min, 249.1136m/z_6.6min,

	281.0665m/z_3.75min, 449.1298m/z_2.23min, 439.2531m/z_6.15min,<br>361.1622m/z_8.15min	242.1757m/z_6.64min, 339.1993m/z_12.63min,<br>281.0665m/z_3.75min, 359.2095m/z_9.76min,<br>449.1298m/z_2.23min, 439.2531m/z_6.15min,<br>361.1622m/z_8.15min
**Shannon**		
forbs<br>*p<0.001* = 9<br>*p<0.01* = 15<br>*p<0.05* = 30<br>total = 54<br><br>grasses<br>*p<0.001* = 12<br>*p<0.01* = 66<br>*p<0.05* = 32<br>total = 110	547.2386m/z_4.38min, 415.1494m/z_5.09min,<br>431.1703m/z_8.05min, 537.1584m/z_4.22min,<br>213.0121m/z_4.88min, 709.3784m/z_5.89min,<br>461.1318m/z_2.82min, 297.0398m/z_7.29min,<br>267.0871m/z_3.62min,<br>325.1835m/z_11.45min, 221.1538m/z_8.15min,<br>597.2157m/z_5.97min, 239.1284m/z_4.91min,<br>353.1941m/z_7.01min, 473.1962m/z_5.42min,<br>397.0925m/z_8.24min, 489.16m/z_3.23min,<br>206.0218m/z_4.44min, 739.2449m/z_5.16min,<br>559.1567m/z_5.66min, 525.2182m/z_2.76min,<br>511.1875m/z_4.49min, 417.1743m/z_5.35min,<br>625.1776m/z_5.21min,<br>361.1622m/z_8.15min, 469.1666m/z_4.27min, 487.1812m/z_4.84min,<br>995.2638m/z_4.25min, 285.2064m/z_7.01min, 313.2374m/z_8.53min,<br>480.9715m/z_6.3min, 297.2426m/z_10.81min, 580.965m/z_7.42min,<br>525.3063m/z_4.42min, 243.1597m/z_7.72min, 191.0346m/z_4.29min,<br>449.1298m/z_2.23min, 269.0851m/z_9.66min, 507.2076m/z_3.72min,<br>204.1236m/z_1.7min, 429.1762m/z_4.96min, 171.066m/z_4.24min,<br>242.1757m/z_6.64min, 236.105m/z_8.15min, 524.9613m/z_6.3min,<br>389.1117m/z_3.09min, 439.2531m/z_6.15min,<br>269.2116m/z_10.42min, 417.1432m/z_3.12min,<br>401.1447m/z_3.46min, 296.9923m/z_3.29min, 339.199m/z_12.23min,<br>451.2167m/z_5.73min, 351.1662m/z_5.88min	547.2386m/z_4.38min, 643.3536m/z_5.41min,<br>549.3397m/z_5.78min, 547.3225m/z_6.19min,<br>377.2265m/z_6.45min, 547.3261m/z_7.39min,<br>867.4731m/z_7.86min, 431.1703m/z_8.05min,<br>537.1584m/z_4.22min, 213.0121m/z_4.88min,<br>325.1997m/z_7.09min, 267.0871m/z_3.62min,<br>325.1835m/z_11.45min, 221.1538m/z_8.15min,<br>239.1284m/z_4.91min, 353.1941m/z_7.01min,<br>511.1875m/z_4.49min, 417.1743m/z_5.35min,<br>285.206m/z_8.94min, 361.1622m/z_8.15min,<br>469.1666m/z_4.27min, 487.1812m/z_4.84min,<br>285.2064m/z_7.01min, 313.2374m/z_8.53min,<br>480.9715m/z_6.3min, 297.2426m/z_10.81min,<br>580.965m/z_7.42min, 525.3063m/z_4.42min,<br>243.1597m/z_7.72min, 191.0346m/z_4.29min,<br>249.1136m/z_6.6min, 449.1298m/z_2.23min, 204.1236m/z_1.7min,<br>373.1393m/z_4.65min, 487.1502m/z_5.44min,<br>377.2061m/z_6.05min, 429.1762m/z_4.96min,<br>171.066m/z_4.24min, 242.1757m/z_6.64min, 236.105m/z_8.15min,<br>329.2323m/z_7.63min, 524.9613m/z_6.3min,<br>439.2531m/z_6.15min, 269.2116m/z_10.42min,<br>417.1432m/z_3.12min, 401.1447m/z_3.46min,<br>296.9923m/z_3.29min, 339.199m/z_12.23min,<br>451.2167m/z_5.73min, 351.1662m/z_5.88min
**Fertilization**		
forbs<br>*p<0.001* = 23<br>*p<0.01* = 7<br>*p<0.05* = 27<br>total = 57<br><br>grasses	329.0882m/z_1.56min, 517.1154m/z_2.3min,<br>299.0775m/z_2.82min, 643.2024m/z_3.15min,<br>737.1919m/z_4.17min, 405.2103m/z_4.62min,<br>615.1831m/z_4.89min, 741.2561m/z_5.17min,<br>557.1862m/z_5.1min, 443.1919m/z_6.08min,<br>571.4201m/z_7.01min, 347.243m/z_8.19min,<br>351.1857m/z_13.79min, 176.0113m/z_4.29min,	299.0775m/z_2.82min, 405.2103m/z_4.62min,<br>352.1748m/z_5.29min, 797.4304m/z_5.43min,<br>661.3633m/z_6.01min, 443.1919m/z_6.08min,<br>567.3523m/z_6.11min, 571.4201m/z_7.01min,<br>221.0812m/z_7.35min, 505.3511m/z_8.25min,<br>381.2249m/z_9.47min, 351.1857m/z_13.79min,

p<0.001 = 16<br>*p<0.01* = 10<br>*p<0.05* = 34<br>total = 60	375.1282m/z_2.37min, 469.1666m/z_4.27min,<br>391.1066m/z_5.34min, 355.2122m/z_9.24min,<br>385.0547m/z_3.97min, 555.1304m/z_2.86min,<br>415.1494m/z_5.09min, 563.1684m/z_5.75min,<br>601.1761m/z_5.04min,<br>388.9898m/z_3.61min, 385.1129m/z_2.46min,<br>283.0368m/z_5.63min, 309.2791m/z_15.05min,<br>191.0197m/z_1.08min, 369.3m/z_13.34min,<br>619.348m/z_8.36min,<br>339.072m/z_3.61min, 527.3192m/z_2.73min,<br>321.2047m/z_12.98min, 593.2503m/z_3.92min,<br>287.2218m/z_8.17min, 441.2519m/z_11.41min,<br>243.1958m/z_8.64min, 365.1172m/z_4.25min,<br>358.1975m/z_2.29min, 549.123m/z_3.55min,<br>385.1118m/z_4.15min, 563.2333m/z_3.62min,<br>451.1265m/z_3.63min, 323.1677m/z_10.06min,<br>432.2342m/z_2.43min, 401.1809m/z_4.27min,<br>259.1287m/z_2.42min, 271.2274m/z_12.35min,<br>477.1801m/z_5.17min, 441.2022m/z_14.72min,<br>303.217m/z_6.74min, 395.1192m/z_3.12min,<br>446.2484m/z_3.19min, 197.0447m/z_3.71min,<br>407.1863m/z_12.36min, 533.1656m/z_4.43min,<br>241.1127m/z_4.78min	469.1666m/z_4.27min, 391.1066m/z_5.34min,<br>355.2122m/z_9.24min, 563.1684m/z_5.75min,<br>557.2409m/z_7.42min, 388.9898m/z_3.61min,<br>385.1129m/z_2.46min, 283.0368m/z_5.63min,<br>309.2791m/z_15.05min, 191.0197m/z_1.08min,<br>369.3m/z_13.34min, 341.1426m/z_7.06min, 355.1583m/z_7.59min,<br>619.348m/z_8.36min,<br>417.2274m/z_5.68min, 519.3296m/z_5.76min,<br>399.1082m/z_6.77min, 339.072m/z_3.61min,<br>527.3192m/z_2.73min, 321.2047m/z_12.98min,<br>311.2218m/z_8.92min, 593.2503m/z_3.92min,<br>577.2292m/z_4.29min, 287.2218m/z_8.17min,<br>441.2519m/z_11.41min, 243.1958m/z_8.64min,<br>365.1172m/z_4.25min, 358.1975m/z_2.29min,<br>549.123m/z_3.55min, 385.1118m/z_4.15min,<br>563.2333m/z_3.62min, 451.1265m/z_3.63min,<br>432.2342m/z_2.43min, 401.1809m/z_4.27min,<br>329.2316m/z_7.29min, 259.1287m/z_2.42min,<br>271.2274m/z_12.35min, 297.243m/z_11.79min,<br>477.1801m/z_5.17min, 441.2022m/z_14.72min,<br>303.217m/z_6.74min, 395.1192m/z_3.12min,<br>446.2484m/z_3.19min, 197.0447m/z_3.71min,<br>315.2171m/z_6.35min, 315.2171m/z_6.97min,<br>533.1656m/z_4.43min, 241.1127m/z_4.78min
**Grazing**<br><u>forbs</u><br>*p<0.001* = 25<br>*p<0.01* = 13<br>*p<0.05* = 21<br>total = 59<br><br><u>grasses</u><br>*p<0.001* = 6<br>*p<0.01* = 12<br>*p<0.05* = 9<br>total = 27	485.1296m/z_2.2min, 563.2302m/z_3.2min,<br>539.2308m/z_3.86min, 561.2156m/z_3.96min,<br>345.1546m/z_4.27min, 523.2246m/z_4.77min,<br>585.1615m/z_4.85min, 523.056m/z_4.92min,<br>621.2369m/z_5.41min, 193.0748m/z_5.49min,<br>211.0247m/z_3.29min, 551.1823m/z_3.89min,<br>579.1277m/z_4.04min, 379.0093m/z_4.17min,<br>607.1315m/z_5.01min, 569.1659m/z_5.7min,<br>451.1596m/z_6.15min, 609.1806m/z_5.16min,<br>529.1547m/z_3.6min, 313.0709m/z_7.2min,<br>947.2767m/z_3.64min, 593.1499m/z_4.69min,	345.1546m/z_4.27min, 229.0774m/z_5.35min,<br>211.0247m/z_3.29min, 551.1823m/z_3.89min,<br>607.1315m/z_5.01min, 635.1963m/z_7.23min,<br>241.0826m/z_1.06min, 609.4118m/z_10.79min,<br>281.0597m/z_6.97min, 193.0501m/z_4.31min,<br>469.1666m/z_4.27min, 301.2006m/z_6.67min,<br>255.2321m/z_13.83min, 347.2424m/z_7.85min,<br>357.2133m/z_1.3min, 527.3192m/z_2.73min,<br>325.1832m/z_11.33min, 125.0963m/z_4.9min,<br>565.2156m/z_3.84min, 295.2269m/z_10.1min,<br>421.2255m/z_11.51min, 242.1757m/z_6.64min,<br>297.0401m/z_7.58min, 309.1708m/z_8.13min,

	591.2266m/z_4.92min, 423.1283m/z_4.39min, 237.0557m/z_8.07min, 193.0501m/z_4.31min, 241.0826m/z_1.06min, 565.2156m/z_3.84min, 401.141m/z_5.86min, 469.1666m/z_4.27min, 301.2006m/z_6.67min, 255.2321m/z_13.83min, 456.2459m/z_2.74min, 281.14m/z_8.95min, 347.2424m/z_7.85min, 357.2133m/z_1.3min, 563.1408m/z_3.76min, 527.3192m/z_2.73min, 325.1832m/z_11.33min, 219.0869m/z_3.63min, 295.2269m/z_10.1min, 421.2255m/z_11.51min, 351.0414m/z_2.95min, 253.0504m/z_6.08min, 242.1757m/z_6.64min, 309.1708m/z_8.13min, 307.1382m/z_3.91min, 591.2079m/z_4.35min, 413.1614m/z_4.64min, 569.1719m/z_1.1min, 421.1269m/z_2.78min, 581.1645m/z_2.81min, 463.1384m/z_4.6min, 463.2154m/z_3.23min, 387.1124m/z_1.09min, 743.2203m/z_2.82min, 491.1744m/z_3.52min, 373.0696m/z_4.21min, 343.2116m/z_8.84min	387.1124m/z_1.09min, 491.1744m/z_3.52min, 343.2116m/z_8.84min
**Mowing**<br>forbs<br>*p<0.001* = 23<br>*p<0.01* = 20<br>*p<0.05* = 46<br>total = 89<br><br>grasses<br>*p<0.001* = 12<br>*p<0.01* = 18<br>*p<0.05* = 50<br>total = 80	171.1022m/z_5.75min, 356.0987m/z_2.52min, 530.1862m/z_3.25min, 363.1637m/z_3.31min, 455.1174m/z_3.44min, 291.0857m/z_3.88min, 465.1397m/z_4.16min, 469.1666m/z_4.27min, 629.1719m/z_4.73min, 243.0295m/z_4.75min, 671.1596m/z_4.91min, 899.2243m/z_4.93min, 599.1631m/z_4.95min, 803.2387m/z_4.9min, 741.2561m/z_5.17min, 785.3102m/z_5.21min, 443.2121m/z_5.45min, 359.1691m/z_5.75min, 563.1684m/z_5.75min, 605.2019m/z_6.51min, 279.2323m/z_13.23min, 351.1857m/z_13.79min, 563.2333m/z_3.62min, 565.2302m/z_3.65min, 445.1888m/z_3.73min, 441.1166m/z_4.51min, 533.1656m/z_4.43min, 432.2342m/z_2.43min, 651.1202m/z_4.23min, 395.203m/z_7.41min, 179.1074m/z_6.19min, 271.2274m/z_12.35min, 309.2039m/z_9.76min,	455.1174m/z_3.44min, 469.1666m/z_4.27min, 365.192m/z_5.75min, 563.1684m/z_5.75min, 477.2808m/z_6.33min, 531.3197m/z_7.46min, 279.2323m/z_13.23min, 171.1022m/z_5.75min, 351.1857m/z_13.79min, 563.2333m/z_3.62min, 355.1583m/z_7.59min, 563.3213m/z_5.93min, 533.1656m/z_4.43min, 432.2342m/z_2.43min, 651.1202m/z_4.23min, 395.203m/z_7.41min, 179.1074m/z_6.19min, 271.2274m/z_12.35min, 341.1426m/z_7.06min, 309.2039m/z_9.76min, 421.2255m/z_11.51min, 173.1117m/z_5.03min, 295.2269m/z_11.31min, 441.2519m/z_11.41min, 309.2791m/z_15.05min, 681.1298m/z_4.27min, 191.0197m/z_1.08min, 283.0368m/z_5.63min, 385.259m/z_10.16min, 301.2006m/z_6.67min, 581.1739m/z_4.26min, 565.2156m/z_3.84min, 311.2218m/z_8.92min, 649.1848m/z_7.89min,

421.2255m/z_11.51min, 173.1117m/z_5.03min,
295.2269m/z_11.31min, 441.2519m/z_11.41min,
309.2791m/z_15.05min, 681.1298m/z_4.27min,
191.0197m/z_1.08min, 283.0368m/z_5.63min,
385.259m/z_10.16min, 301.2006m/z_6.67min,
293.1782m/z_11.32min, 565.2156m/z_3.84min,
611.185m/z_5.05min, 491.2088m/z_2.75min,
319.138m/z_3.38min, 389.1087m/z_1.15min,
413.2898m/z_11.29min, 611.1828m/z_2.64min,
615.2247m/z_4.38min, 253.2167m/z_13min,
452.1914m/z_5.37min, 397.2129m/z_7.49min,
393.1711m/z_11.42min, 439.3414m/z_13.97min,
365.0792m/z_8.4min, 295.2269m/z_10.1min,
385.1129m/z_2.46min, 431.2259m/z_7.21min,
477.1801m/z_5.17min, 327.2161m/z_7min,
377.1578m/z_8.36min, 327.2169m/z_8.34min,
242.1757m/z_6.64min, 527.3192m/z_2.73min,
401.1809m/z_4.27min, 381.2311m/z_13.44min,
243.1958m/z_8.64min, 365.1172m/z_4.25min,
613.2764m/z_6.08min, 339.1993m/z_12.63min,
358.1975m/z_2.29min, 327.2168m/z_6.47min,
173.0816m/z_4.1min, 505.1133m/z_4.94min,
561.2172m/z_3.79min, 283.1909m/z_7.59min,
293.2111m/z_10.79min, 227.2013m/z_12.53min,
397.1813m/z_4.9min, 351.1807m/z_7.58min,
323.1677m/z_10.06min, 311.2219m/z_7.72min,
379.1578m/z_10.2min, 347.0439m/z_3.31min,
241.1127m/z_4.78min, 629.2697m/z_5.24min

611.185m/z_5.05min, 125.0963m/z_4.9min, 413.1498m/z_4.9min,
491.2088m/z_2.75min, 319.138m/z_3.38min,
389.1087m/z_1.15min, 413.2898m/z_11.29min,
615.2247m/z_4.38min, 253.2167m/z_13min,
397.2129m/z_7.49min, 393.1711m/z_11.42min,
439.3414m/z_13.97min, 365.0792m/z_8.4min,
285.0797m/z_4.86min, 295.2269m/z_10.1min,
385.1129m/z_2.46min, 431.2259m/z_7.21min,
477.1801m/z_5.17min, 327.2161m/z_7min, 377.1578m/z_8.36min,
327.2169m/z_8.34min, 242.1757m/z_6.64min,
527.3192m/z_2.73min, 401.1809m/z_4.27min,
381.2311m/z_13.44min, 243.1958m/z_8.64min,
535.3598m/z_7.81min, 365.1172m/z_4.25min,
613.2764m/z_6.08min, 339.1993m/z_12.63min,
358.1975m/z_2.29min, 327.2168m/z_6.47min,
173.0816m/z_4.1min, 505.1133m/z_4.94min,
561.2172m/z_3.79min, 283.1909m/z_7.59min,
293.2111m/z_10.79min, 227.2013m/z_12.53min,
397.1813m/z_4.9min, 351.1807m/z_7.58min,
311.2219m/z_7.72min, 379.1578m/z_10.2min,
347.0439m/z_3.31min, 241.1127m/z_4.78min,
311.201m/z_10.14min, 629.2697m/z_5.24min

pH

<u>forbs</u>
p<0.001 = 14
p<0.01 = 18
p<0.05 = 34
total = 66

<u>grasses</u>
p<0.001 = 11
p<0.01 = 18
p<0.05 = 40
total = 69

437.2375m/z_3.84min, 307.1382m/z_3.91min,
259.1079m/z_3.96min, 523.056m/z_4.92min,
601.1761m/z_5.04min, 311.2217m/z_9.26min,
293.2111m/z_10.79min, 453.0888m/z_4.61min,
313.2378m/z_8.24min, 613.2764m/z_6.08min,
507.2064m/z_3.57min, 629.2697m/z_5.24min,
631.1667m/z_4.48min, 645.1817m/z_4.78min,
607.2289m/z_4.26min, 579.1351m/z_4.88min,
415.1679m/z_5.92min, 285.2065m/z_7.82min,
283.1909m/z_7.59min, 278.0661m/z_4.87min,
387.0694m/z_4.3min, 377.1808m/z_4.13min,
803.3671m/z_4.26min, 611.2253m/z_4.12min,
491.182m/z_4.63min, 171.1022m/z_5.75min,
581.1858m/z_4.06min, 313.2376m/z_8.94min,
585.1615m/z_4.85min, 327.2169m/z_8.34min,
395.2037m/z_6.47min, 293.2109m/z_9.69min,
381.1805m/z_3.94min, 279.2323m/z_13.23min,
467.2166m/z_6.08min, 481.1372m/z_3.29min,
469.1666m/z_4.27min, 563.2333m/z_3.62min,
179.1074m/z_6.19min, 683.1849m/z_4.88min,
711.3953m/z_5.59min, 409.1002m/z_3.12min,
197.0447m/z_3.71min, 565.1658m/z_4.92min,
431.192m/z_4.36min, 347.2424m/z_7.85min,
563.2564m/z_6.68min, 439.0564m/z_1.79min,
421.0906m/z_3.42min, 533.1656m/z_4.43min,
307.1914m/z_8.98min, 337.2032m/z_9.3min,
559.1652m/z_3.15min, 513.1242m/z_2.59min,
243.1235m/z_5.01min, 273.0959m/z_5.67min,
267.196m/z_9.82min, 561.2172m/z_3.79min,
287.1194m/z_4.84min, 327.2168m/z_6.47min,
277.067m/z_4.92min, 325.1288m/z_6.05min,
391.0338m/z_3.5min, 561.1753m/z_3.3min,
271.05m/z_4.91min, 297.2431m/z_12.02min

293.2111m/z_10.79min, 437.2375m/z_3.84min,
311.2217m/z_9.26min, 453.0888m/z_4.61min,
313.2378m/z_8.24min, 613.2764m/z_6.08min,
487.3407m/z_9.7min, 551.3568m/z_6.4min, 629.2697m/z_5.24min,
567.3523m/z_6.11min, 365.2676m/z_9.63min,
607.2289m/z_4.26min, 415.1679m/z_5.92min,
285.2065m/z_7.82min, 283.1909m/z_7.59min,
549.3422m/z_7.18min, 269.2112m/z_11.31min,
387.0694m/z_4.3min, 567.3523m/z_5min, 803.3671m/z_4.26min,
611.2253m/z_4.12min, 491.182m/z_4.63min,
171.1022m/z_5.75min, 581.1858m/z_4.06min,
313.2376m/z_8.94min, 327.2169m/z_8.34min,
561.4099m/z_9.3min, 395.2037m/z_6.47min,
293.2109m/z_9.69min,
381.1805m/z_3.94min, 419.2319m/z_6.48min,
319.2275m/z_10.24min, 279.2323m/z_13.23min,
467.2166m/z_6.08min, 267.1249m/z_5.53min,
469.1666m/z_4.27min, 563.2333m/z_3.62min,
179.1074m/z_6.19min, 311.2216m/z_8.27min,
409.1002m/z_3.12min, 197.0447m/z_3.71min,
565.1658m/z_4.92min, 431.192m/z_4.36min,
295.2272m/z_11.84min, 347.2424m/z_7.85min,
563.2564m/z_6.68min, 439.0564m/z_1.79min,
533.1656m/z_4.43min, 525.2404m/z_5.35min,
307.1914m/z_8.98min, 337.2032m/z_9.3min,
559.1652m/z_3.15min, 469.1839m/z_5.89min,
243.1235m/z_5.01min, 273.0959m/z_5.67min,
267.196m/z_9.82min, 561.2172m/z_3.79min,
295.2273m/z_9.82min, 287.1194m/z_4.84min,
171.1024m/z_5.36min, 327.2168m/z_6.47min,
277.067m/z_4.92min, 325.1288m/z_6.05min, 391.0338m/z_3.5min,
540.221m/z_6.61min, 561.1753m/z_3.3min, 125.0963m/z_4.9min,
271.05m/z_4.91min, 297.2431m/z_12.02min

Soil moisture

forbs
$p<0.001$ = 51
$p<0.01$ = 72
$p<0.05$ = 36
total = 159

grasses
$p<0.001$ = 39
$p<0.01$ = 44
$p<0.05$ = 39
total = 122

581.1858m/z_4.06min, 427.1959m/z_4.52min,
239.1284m/z_4.91min, 285.2064m/z_7.01min,
269.2116m/z_10.42min, 351.0414m/z_2.95min,
545.1494m/z_3.06min, 445.1907m/z_3.28min,
579.1308m/z_3.64min, 637.1419m/z_3.67min,
607.2195m/z_3.71min, 563.1408m/z_3.76min,
664.1887m/z_3.76min, 678.2013m/z_3.81min,
379.0093m/z_4.17min, 469.1666m/z_4.27min,
581.1881m/z_4.72min, 633.2376m/z_4.8min,
206.984m/z_5.11min, 609.1806m/z_5.16min,
385.0918m/z_5.96min, 463.1626m/z_6.08min,
327.2168m/z_6.47min, 242.1757m/z_6.64min,
329.2324m/z_6.83min, 283.1909m/z_7.59min,
267.196m/z_9.82min, 401.1809m/z_4.27min,
547.2386m/z_4.38min, 255.0302m/z_6.51min,
505.1345m/z_6.43min, 819.4185m/z_6.81min,
253.0503m/z_7.02min, 243.1235m/z_5.01min,
255.0302m/z_8.01min, 585.2387m/z_5.72min,
285.2065m/z_8.13min, 467.2166m/z_6.08min,
283.0534m/z_8.43min, 563.2333m/z_3.62min,
369.0952m/z_8.75min, 273.0959m/z_5.67min,
511.1454m/z_4.08min, 607.1697m/z_3.94min,
431.0977m/z_5.62min, 266.0188m/z_3.72min,
259.1079m/z_3.96min, 299.0213m/z_5.23min,
507.2071m/z_5.2min, 267.0304m/z_9.64min,
423.0583m/z_3.65min,
456.2459m/z_2.74min, 281.14m/z_8.95min,
431.1703m/z_8.05min, 421.2255m/z_11.51min,
433.1873m/z_3.58min, 551.1765m/z_3.6min,
295.2269m/z_10.1min, 397.2129m/z_7.49min,
311.2219m/z_7.72min, 293.2111m/z_9.42min,
293.2111m/z_10.79min, 329.2324m/z_7.49min,
401.1808m/z_4.04min, 253.2167m/z_13min,
291.196m/z_10.14min, 255.2321m/z_13.83min,
327.2166m/z_7.41min, 313.2277m/z_9.69min,
165.0192m/z_3.09min, 373.1125m/z_1.64min,
467.1439m/z_2.45min, 681.223m/z_2.93min,

637.1419m/z_3.67min, 664.1887m/z_3.76min,
678.2013m/z_3.81min, 469.1666m/z_4.27min,
607.1664m/z_4.67min, 797.4304m/z_5.43min,
535.2907m/z_5.69min, 461.2202m/z_5.99min,
535.3267m/z_6.08min, 327.2168m/z_6.47min,
242.1757m/z_6.64min, 329.2324m/z_6.83min,
519.3298m/z_7.55min, 283.1909m/z_7.59min,
379.2098m/z_7.72min, 505.3511m/z_8.25min,
523.0414m/z_9.61min, 267.196m/z_9.82min,
521.028m/z_10.01min, 557.0016m/z_10.25min,
401.1809m/z_4.27min, 547.2386m/z_4.38min,
581.1858m/z_4.06min, 427.1959m/z_4.52min,
239.1284m/z_4.91min, 285.2064m/z_7.01min,
269.2116m/z_10.42min, 243.1235m/z_5.01min,
585.2387m/z_5.72min, 331.1632m/z_8.91min,
285.2065m/z_8.13min, 467.2166m/z_6.08min,
283.0534m/z_8.43min, 563.2333m/z_3.62min,
273.0959m/z_5.67min, 365.2676m/z_9.63min,
266.0188m/z_3.72min, 651.408m/z_7.73min,
423.0583m/z_3.65min,
371.1708m/z_4.64min, 463.2347m/z_6.62min,
431.1703m/z_8.05min, 421.2255m/z_11.51min,
433.1873m/z_3.58min, 551.1765m/z_3.6min,
295.2269m/z_10.1min, 397.2129m/z_7.49min,
311.2219m/z_7.72min, 293.2111m/z_9.42min,
293.2111m/z_10.79min, 329.2324m/z_7.49min,
401.1808m/z_4.04min, 253.2167m/z_13min,
291.196m/z_10.14min, 255.2321m/z_13.83min,
327.2166m/z_7.41min, 313.2277m/z_9.69min,
179.1074m/z_6.19min, 803.3671m/z_4.26min,
239.1283m/z_4.7min, 295.2273m/z_9.82min,
563.2564m/z_6.68min, 267.1312m/z_5.99min,
561.4099m/z_9.3min, 409.0444m/z_2.6min, 327.2169m/z_8.34min,
293.2109m/z_9.69min, 533.1656m/z_4.43min,
553.2367m/z_3.8min, 611.2253m/z_4.12min,
241.0799m/z_1.39min, 337.2032m/z_9.3min,
613.2764m/z_6.08min, 389.1236m/z_4.37min,

173.0236m/z_3.17min, 300.0873m/z_3.33min,
567.2278m/z_3.62min, 625.219m/z_3.65min,
683.2633m/z_3.97min, 788.2898m/z_3.97min,
665.2427m/z_3.98min, 829.3392m/z_4.25min,
551.1554m/z_4.26min, 531.1447m/z_4.2min,
541.1143m/z_4.53min, 429.1638m/z_4.59min,
481.0991m/z_5.08min, 745.3056m/z_5.2min,
388.1394m/z_5.64min, 481.1394m/z_5.69min,
269.0463m/z_5.97min, 481.1333m/z_5.98min,
381.1684m/z_5.99min, 343.1714m/z_5min,
607.2495m/z_6.04min, 637.1892m/z_6.63min,
179.1074m/z_6.19min, 239.0351m/z_7.94min,
803.3671m/z_4.26min, 239.1283m/z_4.7min,
563.2564m/z_6.68min, 267.1312m/z_5.99min,
409.0444m/z_2.6min, 327.2169m/z_8.34min,
293.2109m/z_9.69min, 533.1656m/z_4.43min,
553.2367m/z_3.8min, 515.1521m/z_6.25min,
611.2253m/z_4.12min, 241.0799m/z_1.39min,
337.2032m/z_9.3min, 613.2764m/z_6.08min,
389.1236m/z_4.37min, 589.0386m/z_6.18min,
457.2062m/z_4.69min, 397.0925m/z_8.24min,
295.2269m/z_11.31min, 631.1637m/z_3.93min,
383.3513m/z_15min, 395.203m/z_7.41min,
239.0349m/z_6.21min, 521.1852m/z_4.12min,
339.1988m/z_12.17min, 563.2179m/z_3.98min,
625.1763m/z_5.07min, 295.227m/z_10.35min,
499.1984m/z_3.82min, 389.1232m/z_4.43min,
671.291m/z_6.56min, 171.1022m/z_5.75min,
565.1771m/z_3.45min, 271.2274m/z_12.35min,
285.2065m/z_7.82min, 311.2217m/z_9.26min,
313.2378m/z_8.24min, 439.0564m/z_1.79min,
255.1034m/z_6.19min, 269.2099m/z_9.3min,
173.1117m/z_5.03min, 279.2323m/z_13.23min,
325.1288m/z_6.05min, 433.2051m/z_3.99min,
431.2638m/z_7.55min, 299.0209m/z_6.2min,
297.1522m/z_9.69min, 373.0696m/z_4.21min,
411.1901m/z_5.02min, 563.1406m/z_3.88min,
309.2038m/z_8.66min, 197.0452m/z_3.77min,
363.2138m/z_10.35min, 593.1603m/z_4.02min,

457.2062m/z_4.69min, 351.2155m/z_7.02min,
295.2269m/z_11.31min, 631.1637m/z_3.93min,
383.3513m/z_15min, 395.203m/z_7.41min, 521.1852m/z_4.12min,
339.1988m/z_12.17min, 563.2179m/z_3.98min,
319.2275m/z_10.24min, 295.227m/z_10.35min,
499.1984m/z_3.82min, 389.1232m/z_4.43min,
671.291m/z_6.56min, 171.1022m/z_5.75min, 125.0963m/z_4.9min,
271.2274m/z_12.35min, 285.2065m/z_7.82min,
311.2217m/z_9.26min, 313.2378m/z_8.24min,
439.0564m/z_1.79min, 255.1034m/z_6.19min,
525.2404m/z_5.35min, 309.2051m/z_8min, 269.2099m/z_9.3min,
173.1117m/z_5.03min, 279.2323m/z_13.23min,
325.1288m/z_6.05min, 433.2051m/z_3.99min,
476.2757m/z_9.66min, 431.2638m/z_7.55min,
297.1522m/z_9.69min, 411.1901m/z_5.02min,
424.9673m/z_5.14min, 574.958m/z_6.85min,
563.1406m/z_3.88min, 309.2038m/z_8.66min,
197.0452m/z_3.77min, 363.2138m/z_10.35min,
593.1603m/z_4.02min, 297.2426m/z_10.81min,
299.1757m/z_9.54min, 293.2101m/z_10.65min,
295.2263m/z_11.17min, 431.2259m/z_7.21min,
348.9199m/z_7.09min, 397.2192m/z_6.83min,
180.9897m/z_4.28min

	297.2426m/z_10.81min, 299.1757m/z_9.54min,<br>293.2101m/z_10.65min, 295.2263m/z_11.17min,<br>431.2259m/z_7.21min, 348.9199m/z_7.09min,<br>397.2192m/z_6.83min, 180.9897m/z_4.28min	
**soil texture**<br>forbs<br>*p<0.001* = 38<br>*p<0.01* = 20<br>*p<0.05* = 37<br>total = 95<br><br>grasses<br>*p<0.001* = 14<br>*p<0.01* = 17<br>*p<0.05* = 47<br>total = 78	485.1296m/z_2.2min, 241.0716m/z_2.67min,<br>137.0244m/z_2.74min, 525.2182m/z_2.76min,<br>530.1862m/z_3.25min, 523.2316m/z_3.3min,<br>655.2221m/z_3.4min, 339.1023m/z_3.55min,<br>623.1629m/z_3.92min, 340.1031m/z_3.99min,<br>379.0093m/z_4.17min, 631.1667m/z_4.48min,<br>761.2136m/z_4.49min, 615.2199m/z_4.54min,<br>409.1094m/z_4.63min, 475.2175m/z_4.6min,<br>645.1817m/z_4.78min, 653.149m/z_4.83min,<br>441.1079m/z_5.08min, 443.2276m/z_5.16min,<br>313.0358m/z_5.21min, 366.0968m/z_5.33min,<br>179.0708m/z_5.71min, 709.3784m/z_5.89min,<br>347.243m/z_8.19min, 591.2266m/z_4.92min,<br>447.1655m/z_7.29min, 337.2032m/z_9.3min,<br>655.2235m/z_4.42min, 509.0895m/z_2.04min,<br>299.0775m/z_2.82min, 461.1306m/z_4.02min,<br>507.1528m/z_4.71min, 741.2561m/z_5.17min,<br>267.0871m/z_3.62min, 669.2214m/z_3.33min,<br>213.0121m/z_4.88min, 549.123m/z_3.55min,<br>493.1571m/z_2.1min, 501.32m/z_9.34min,<br>241.0826m/z_1.06min, 521.1852m/z_4.12min,<br>279.2323m/z_13.23min, 389.1117m/z_3.09min,<br>393.1711m/z_11.42min, 547.2386m/z_4.38min,<br>329.2324m/z_7.49min, 469.1666m/z_4.27min,<br>389.1087m/z_1.15min, 379.2096m/z_9.07min,<br>456.2459m/z_2.74min, 281.14m/z_8.95min,<br>671.291m/z_6.56min, 193.0504m/z_3.75min,<br>357.2133m/z_1.3min, 381.2311m/z_13.44min,<br>288.9479m/z_7.1min, 283.0461m/z_6.61min,<br>367.0868m/z_3.48min, 329.2324m/z_6.83min,<br>385.1118m/z_4.15min, 395.2428m/z_8.5min,<br>555.1466m/z_5.49min, 429.1377m/z_3.26min,<br>467.1615m/z_3.73min, 331.205m/z_8.51min,	337.2032m/z_9.3min, 339.1023m/z_3.55min,<br>623.1629m/z_3.92min, 667.1503m/z_4.39min,<br>229.0774m/z_5.35min, 305.1396m/z_5.8min,<br>353.2327m/z_7.33min, 533.3441m/z_8.44min,<br>655.2235m/z_4.42min, 299.0775m/z_2.82min,<br>549.123m/z_3.55min, 267.0871m/z_3.62min,<br>669.2214m/z_3.33min, 213.0121m/z_4.88min,<br>241.0826m/z_1.06min, 609.4118m/z_10.79min,<br>521.1852m/z_4.12min, 365.2304m/z_10.77min,<br>279.2323m/z_13.23min, 393.1711m/z_11.42min,<br>547.2386m/z_4.38min, 329.2324m/z_7.49min,<br>469.1666m/z_4.27min, 357.2133m/z_1.3min,<br>381.2311m/z_13.44min, 671.291m/z_6.56min,<br>193.0504m/z_3.75min, 389.1087m/z_1.15min,<br>379.2096m/z_9.07min, 288.9479m/z_7.1min,<br>309.1955m/z_9.15min,<br>861.2448m/z_5.31min, 367.0868m/z_3.48min,<br>329.2324m/z_6.83min, 385.1118m/z_4.15min,<br>395.2428m/z_8.5min, 429.1377m/z_3.26min,<br>467.1615m/z_3.73min, 451.1952m/z_4.57min,<br>553.3379m/z_4.68min, 231.1216m/z_4.76min,<br>659.3782m/z_4.89min, 315.0521m/z_4.97min,<br>381.0967m/z_5.34min, 617.2243m/z_5.73min, 297.04m/z_6.95min,<br>521.3466m/z_7.03min, 649.3931m/z_7.35min,<br>295.0239m/z_8.07min, 305.1741m/z_8.2min, 331.205m/z_8.51min,<br>295.2269m/z_10.1min, 293.2109m/z_9.69min,<br>275.0919m/z_5.38min, 242.1757m/z_6.64min,<br>323.219m/z_13.85min, 431.1703m/z_8.05min,<br>365.1495m/z_9.47min, 311.2219m/z_7.72min,<br>355.2085m/z_8.17min, 200.9961m/z_4.08min,<br>293.2111m/z_9.42min, 227.2013m/z_12.53min,<br>343.0515m/z_4.34min, 480.9715m/z_6.3min,<br>297.2426m/z_10.81min, 599.1424m/z_4.41min,

	581.182m/z_3.62min, 295.2269m/z_10.1min,<br>293.2109m/z_9.69min, 275.0919m/z_5.38min,<br>861.2448m/z_5.31min, 242.1757m/z_6.64min,<br>323.219m/z_13.85min, 431.1703m/z_8.05min,<br>365.1495m/z_9.47min, 311.2219m/z_7.72min,<br>355.2085m/z_8.17min, 200.9961m/z_4.08min,<br>589.0386m/z_6.18min, 293.2111m/z_9.42min,<br>227.2013m/z_12.53min, 343.0515m/z_4.34min,<br>465.1179m/z_2.92min, 480.9715m/z_6.3min,<br>297.2426m/z_10.81min, 327.2168m/z_6.47min,<br>295.227m/z_10.35min, 239.1284m/z_4.91min,<br>467.0597m/z_3.97min, 201.1129m/z_5.67min,<br>440.9281m/z_7.05min, 361.1622m/z_8.15min,<br>388.9898m/z_3.61min, 327.2111m/z_7.58min,<br>731.14m/z_5.5min	327.2168m/z_6.47min, 295.227m/z_10.35min,<br>239.1284m/z_4.91min, 467.0597m/z_3.97min,<br>201.1129m/z_5.67min, 285.0797m/z_4.86min,<br>440.9281m/z_7.05min, 361.1622m/z_8.15min,<br>463.2347m/z_6.62min, 388.9898m/z_3.61min,<br>327.2111m/z_7.58min
**soil type**		
forbs<br>*p<0.001* = 19<br>*p<0.01* = 13<br>*p<0.05* = 34<br>total = 66<br><br>grasses<br>*p<0.001* = 15<br>*p<0.01* = 10<br>*p<0.05* = 30<br>total = 55	525.2182m/z_2.76min, 375.1311m/z_3.29min,<br>505.1898m/z_3.45min, 607.2195m/z_3.71min,<br>527.136m/z_3.93min, 345.1546m/z_4.27min,<br>507.2066m/z_4.98min, 473.1653m/z_5.31min,<br>709.3784m/z_5.89min, 385.0918m/z_5.96min,<br>431.1703m/z_8.05min, 423.1283m/z_4.39min,<br>547.2386m/z_4.38min, 461.1318m/z_2.82min,<br>221.1538m/z_8.15min, 351.0115m/z_6.24min,<br>485.1296m/z_2.2min, 655.2799m/z_4.97min,<br>297.0398m/z_7.29min,<br>507.2071m/z_5.2min, 269.2481m/z_14.22min,<br>353.1941m/z_7.01min, 285.2064m/z_7.01min,<br>389.1117m/z_3.09min, 325.1835m/z_11.45min,<br>511.1875m/z_4.49min, 417.1743m/z_5.35min,<br>359.0985m/z_1.6min, 389.1117m/z_3.09min,<br>325.1835m/z_11.45min, 313.2374m/z_8.53min,<br>487.1812m/z_4.84min,<br>367.1042m/z_3.25min, 236.105m/z_8.15min,<br>293.1751m/z_8.15min, 329.2324m/z_7.49min,<br>191.0346m/z_4.29min, 231.0301m/z_7.39min,<br>357.2133m/z_1.3min, 469.1666m/z_4.27min,	345.1546m/z_4.27min, 699.3939m/z_4.6min,<br>815.4389m/z_4.83min, 813.4231m/z_5.11min,<br>321.1701m/z_6.94min, 593.403m/z_7min, 431.1703m/z_8.05min,<br>317.1389m/z_8.15min, 355.249m/z_10.36min,<br>547.2386m/z_4.38min, 603.3586m/z_5.56min,<br>573.3478m/z_5.85min, 587.3618m/z_6.31min,<br>733.4564m/z_6.96min, 221.1538m/z_8.15min,<br>285.2064m/z_7.01min, 353.1941m/z_7.01min,<br>325.1835m/z_11.45min, 303.0909m/z_4.04min,<br>267.0871m/z_3.62min, 511.1875m/z_4.49min,<br>417.1743m/z_5.35min, 313.2374m/z_8.53min,<br>487.1812m/z_4.84min, 329.2323m/z_7.63min,<br>469.1666m/z_4.27min, 367.1042m/z_3.25min,<br>317.2119m/z_10.6min, 236.105m/z_8.15min,<br>293.1751m/z_8.15min, 369.1718m/z_8.13min,<br>329.2324m/z_7.49min, 191.0346m/z_4.29min,<br>357.2133m/z_1.3min, 243.1597m/z_7.72min, 171.066m/z_4.24min,<br>297.2426m/z_10.81min, 239.1284m/z_4.91min,<br>269.2116m/z_10.42min, 325.1833m/z_11.51min,<br>429.1762m/z_4.96min, 361.1622m/z_8.15min,<br>285.2065m/z_7.82min, 379.2096m/z_9.07min,

	243.1597m/z_7.72min, 171.066m/z_4.24min,<br>625.1776m/z_5.21min, 297.2426m/z_10.81min,<br>239.1284m/z_4.91min, 269.2116m/z_10.42min,<br>325.1833m/z_11.51min, 429.1762m/z_4.96min,<br>507.2076m/z_3.72min, 369.0952m/z_8.75min,<br>995.2638m/z_4.25min, 361.1622m/z_8.15min,<br>285.2065m/z_7.82min, 379.2096m/z_9.07min,<br>397.1813m/z_4.9min, 269.0851m/z_9.66min,<br>371.0972m/z_3.8min, 397.2129m/z_7.49min,<br>381.2311m/z_13.44min, 431.2259m/z_7.21min,<br>427.1959m/z_4.52min, 339.199m/z_12.23min,<br>433.1873m/z_3.58min, 291.196m/z_9.42min,<br>473.3262m/z_12.65min, 327.2169m/z_8.34min	397.1813m/z_4.9min, 371.0972m/z_3.8min, 397.2129m/z_7.49min,<br>381.2311m/z_13.44min, 431.2259m/z_7.21min,<br>427.1959m/z_4.52min, 339.199m/z_12.23min,<br>433.1873m/z_3.58min, 291.196m/z_9.42min,<br>473.3262m/z_12.65min, 327.2169m/z_8.34min
**TC**<br>forbs<br>$p<0.001$ = 15<br>$p<0.01$ = 21<br>$p<0.05$ = 15<br>total = 51<br><br>grasses<br>$p<0.001$ = 19<br>$p<0.01$ = 5<br>$p<0.05$ = 15<br>total = 39	329.0882m/z_1.56min, 461.1301m/z_2.57min,<br>643.2213m/z_3.44min, 637.1419m/z_3.67min,<br>664.1887m/z_3.76min, 525.1612m/z_3.78min,<br>678.2013m/z_3.81min, 340.1031m/z_3.99min,<br>761.2136m/z_4.49min, 615.2199m/z_4.54min,<br>433.0282m/z_4.99min, 441.1079m/z_5.08min,<br>295.1364m/z_8.45min, 625.2039m/z_8.75min,<br>251.0559m/z_3.64min,<br>377.1792m/z_2.89min, 591.1649m/z_3.07min,<br>319.0859m/z_3.13min, 275.0856m/z_3.51min,<br>535.2379m/z_3.59min, 493.1943m/z_3.61min,<br>445.1185m/z_3.86min, 455.1035m/z_4.11min,<br>635.1613m/z_4.38min, 345.1539m/z_4.68min,<br>489.197m/z_5.48min, 517.2283m/z_5.85min,<br>259.0824m/z_6.02min, 445.2418m/z_6.06min,<br>257.1397m/z_6.29min, 401.124m/z_6.55min,<br>311.1681m/z_10.05min, 421.0906m/z_3.42min,<br>215.1284m/z_6.38min, 507.2064m/z_3.57min,<br>358.1975m/z_2.29min,<br>293.1751m/z_8.15min, 633.1408m/z_3.41min,<br>423.0583m/z_3.65min, 239.1284m/z_4.91min,<br>385.1129m/z_2.46min, 266.0188m/z_3.72min,<br>293.1787m/z_11.2min, 351.0414m/z_2.95min,<br>297.2423m/z_10.74min, 519.186m/z_4.64min,	461.1301m/z_2.57min, 637.1419m/z_3.67min,<br>664.1887m/z_3.76min, 678.2013m/z_3.81min,<br>667.1503m/z_4.39min, 607.1664m/z_4.67min,<br>433.0282m/z_4.99min, 975.5116m/z_6.73min,<br>533.3303m/z_7.16min, 329.2316m/z_7.29min,<br>353.2327m/z_7.33min, 325.1998m/z_7.78min,<br>505.3511m/z_8.25min, 547.3252m/z_6.98min,<br>797.4304m/z_5.43min, 533.3441m/z_8.44min,<br>573.2494m/z_4.26min, 545.2227m/z_4.89min,<br>251.0559m/z_3.64min,<br>311.1681m/z_10.05min, 215.1284m/z_6.38min,<br>268.116m/z_4.81min, 491.2121m/z_4.98min,<br>358.1975m/z_2.29min,<br>293.1751m/z_8.15min, 423.0583m/z_3.65min,<br>239.1284m/z_4.91min, 385.1129m/z_2.46min,<br>266.0188m/z_3.72min, 293.1787m/z_11.2min,<br>297.2423m/z_10.74min, 519.186m/z_4.64min,<br>335.1889m/z_11.52min, 529.1855m/z_4.56min,<br>393.171m/z_10.92min, 311.2214m/z_9.06min,<br>269.0454m/z_6.92min, 229.1804m/z_7.96min,<br>653.0978m/z_7.03min

	335.1889m/z_11.52min, 529.1855m/z_4.56min,<br>311.2214m/z_9.06min, 269.0454m/z_6.92min,<br>229.1804m/z_7.96min	
**TN**<br>forbs<br>$p<0.001$ = 4<br>$p<0.01$ = 9<br>$p<0.05$ = 22<br>total = 35<br><br>grasses<br>$p<0.001$ = 3<br>$p<0.01$ = 8<br>$p<0.05$ = 20<br>total = 31	455.1174m/z_3.44min, 555.1466m/z_5.49min,<br>200.1286m/z_4.83min, 327.2161m/z_7min,<br>311.1681m/z_10.05min, 239.1284m/z_4.91min,<br>297.2423m/z_10.74min, 295.2269m/z_10.1min,<br>293.1787m/z_11.2min, 617.1089m/z_3.54min,<br>215.1284m/z_6.38min, 423.0583m/z_3.65min,<br>585.1836m/z_3.67min,<br>456.2459m/z_2.74min, 281.14m/z_8.95min,<br>329.2324m/z_7.49min, 563.2898m/z_5.57min,<br>311.2214m/z_9.06min, 229.1804m/z_7.96min,<br>313.2376m/z_8.94min, 266.0188m/z_3.72min,<br>341.1073m/z_5.74min, 589.1887m/z_4.35min,<br>651.2143m/z_3.82min, 611.185m/z_5.05min,<br>529.1855m/z_4.56min, 585.2387m/z_5.72min,<br>269.0454m/z_6.92min, 335.1889m/z_11.52min,<br>547.2386m/z_4.38min, 372.123m/z_8.35min,<br>201.1129m/z_5.67min, 293.2111m/z_10.79min,<br>529.0899m/z_8.99min, 551.1823m/z_3.89min	455.1174m/z_3.44min, 200.1286m/z_4.83min, 327.2161m/z_7min,<br>311.1681m/z_10.05min, 239.1284m/z_4.91min,<br>297.2423m/z_10.74min, 295.2269m/z_10.1min,<br>293.1787m/z_11.2min, 585.1836m/z_3.67min,<br>215.1284m/z_6.38min, 423.0583m/z_3.65min,<br>329.2324m/z_7.49min, 563.2898m/z_5.57min,<br>311.2214m/z_9.06min, 229.1804m/z_7.96min,<br>311.201m/z_10.14min, 313.2376m/z_8.94min,<br>266.0188m/z_3.72min, 341.1073m/z_5.74min,<br>651.2143m/z_3.82min, 611.185m/z_5.05min,<br>529.1855m/z_4.56min, 585.2387m/z_5.72min,<br>269.0454m/z_6.92min, 335.1889m/z_11.52min,<br>547.2386m/z_4.38min, 393.171m/z_10.92min,<br>201.1129m/z_5.67min, 293.2111m/z_10.79min,<br>144.0458m/z_4.62min, 551.1823m/z_3.89min
**Precipitation**<br>forbs<br>$p<0.001$ = 12<br>$p<0.01$ = 31<br>$p<0.05$ = 52<br>total = 95<br><br>grasses<br>$p<0.001$ = 9<br>$p<0.01$ = 22<br>$p<0.05$ = 48<br>total = 79	517.1154m/z_2.3min, 595.2582m/z_3.65min,<br>213.0121m/z_4.88min, 683.1849m/z_4.88min,<br>557.1862m/z_5.1min, 567.3263m/z_5.22min,<br>711.3953m/z_5.59min, 321.2095m/z_13.8min,<br>479.269m/z_6.24min, 381.0621m/z_7.17min,<br>328.212m/z_8.92min, 338.1388m/z_8.53min,<br>479.269m/z_6.24min, 381.0621m/z_7.17min,<br>328.212m/z_8.92min, 338.1388m/z_8.53min,<br>587.2182m/z_5.35min, 267.1597m/z_5.66min,<br>613.2667m/z_5.66min, 463.1453m/z_1.05min,<br>197.0453m/z_1.42min, 387.0977m/z_2.06min,<br>491.1753m/z_3.39min, 343.137m/z_3.8min,<br>413.2169m/z_5.37min, 325.0916m/z_5.56min,<br>421.1158m/z_5.6min, 311.1681m/z_10.63min,	475.1251m/z_7.03min, 321.2095m/z_13.8min,<br>213.0121m/z_4.88min, 567.3263m/z_5.22min,<br>331.1632m/z_8.91min, 533.345m/z_7.63min, 479.269m/z_6.24min,<br>328.212m/z_8.92min, 365.2676m/z_9.63min,<br>631.1637m/z_3.93min, 401.1809m/z_4.27min,<br>323.219m/z_13.85min, 337.2032m/z_9.3min,<br>321.0538m/z_4.14min, 350.9167m/z_7.05min,<br>543.1993m/z_4.16min, 303.1195m/z_4.19min,<br>251.0917m/z_4.74min, 273.1695m/z_5.1min,<br>356.2425m/z_10.21min, 525.2404m/z_5.35min,<br>351.2143m/z_5.19min, 531.2955m/z_6.62min,<br>659.4715m/z_6.83min, 411.1901m/z_5.02min,<br>561.4127m/z_9.51min, 144.0458m/z_4.62min,

631.1637m/z_3.93min, 401.1809m/z_4.27min,
323.219m/z_13.85min, 337.2032m/z_9.3min,
441.1166m/z_4.51min, 321.0538m/z_4.14min,
350.9167m/z_7.05min, 507.2071m/z_5.2min,
324.1236m/z_7.94min, 537.1978m/z_3.9min,
659.4715m/z_6.83min, 411.1901m/z_5.02min,
973.4448m/z_3.79min, 561.4127m/z_9.51min,
273.0959m/z_5.67min, 389.1117m/z_3.09min,
271.2274m/z_12.35min, 377.1776m/z_4.31min,
295.2263m/z_11.17min,
293.1751m/z_8.15min, 283.0719m/z_5.8min,
283.0534m/z_8.43min, 365.1495m/z_9.47min,
447.1152m/z_2.45min, 643.2079m/z_4.22min,
269.0483m/z_7.72min, 269.2116m/z_10.42min,
563.2564m/z_6.68min, 547.2386m/z_4.38min,
313.2376m/z_8.94min, 463.1818m/z_4.3min,
239.1284m/z_4.91min, 391.1601m/z_3.36min,
529.0899m/z_8.99min, 507.2356m/z_6.17min,
277.0712m/z_1.4min, 231.0297m/z_4.3min,
377.1806m/z_3.7min, 313.2378m/z_8.24min,
565.1771m/z_3.45min, 489.1246m/z_2.58min,
311.2219m/z_7.72min, 363.2138m/z_10.35min,
431.1338m/z_5.86min, 481.1363m/z_3.57min,
655.4203m/z_6.48min, 455.3366m/z_12.92min,
393.1711m/z_11.42min, 327.2169m/z_8.34min,
329.0875m/z_1.86min, 423.1636m/z_4.01min,
291.196m/z_10.14min, 745.2468m/z_4.93min,
293.2109m/z_9.69min, 365.1394m/z_9.54min,
327.2111m/z_7.58min, 551.1765m/z_3.6min,
283.0359m/z_6.15min, 297.2426m/z_10.81min,
563.1449m/z_5.35min, 519.186m/z_4.64min,
371.1121m/z_5.59min, 373.113m/z_2.01min,
259.0999m/z_7.07min, 619.1862m/z_3.12min,
393.2488m/z_8.98min, 163.04m/z_4min, 487.3414m/z_8.82min,
395.1667m/z_4.76min, 295.2269m/z_11.31min,
593.1416m/z_3.6min

273.0959m/z_5.67min, 271.2274m/z_12.35min,
377.1776m/z_4.31min, 295.2263m/z_11.17min,
293.1751m/z_8.15min, 339.1987m/z_11.79min,
283.0719m/z_5.8min, 283.0534m/z_8.43min,
365.1495m/z_9.47min, 447.1152m/z_2.45min,
269.0483m/z_7.72min, 269.2116m/z_10.42min,
563.2564m/z_6.68min, 547.2386m/z_4.38min,
319.2275m/z_10.24min, 313.2376m/z_8.94min,
239.1284m/z_4.91min, 277.0712m/z_1.4min,
313.2378m/z_8.24min, 433.1855m/z_5.14min,
311.2219m/z_7.72min, 363.2138m/z_10.35min,
481.1363m/z_3.57min, 655.4203m/z_6.48min,
455.3366m/z_12.92min, 393.1711m/z_11.42min,
327.2169m/z_8.34min, 329.0875m/z_1.86min,
423.1636m/z_4.01min, 291.196m/z_10.14min,
337.1945m/z_10.53min, 293.2109m/z_9.69min,
365.1394m/z_9.54min, 327.2111m/z_7.58min,
295.2273m/z_9.82min, 551.1765m/z_3.6min,
297.2426m/z_10.81min, 563.1449m/z_5.35min,
353.2317m/z_7.98min, 519.186m/z_4.64min,
371.1121m/z_5.59min, 373.113m/z_2.01min,
259.0999m/z_7.07min, 619.1862m/z_3.12min,
393.2488m/z_8.98min, 163.04m/z_4min, 399.1662m/z_4.4min,
487.3414m/z_8.82min, 395.1667m/z_4.76min,
295.2269m/z_11.31min, 557.3813m/z_9.82min,
593.1416m/z_3.6min

**T(10)**		
forbs<br>*p<0.001* = 66<br>*p<0.01* = 13<br>*p<0.05* = 25<br>total = 46<br><br>grasses<br>*p<0.001* = 4<br>*p<0.01* = 14<br>*p<0.05* = 27<br>total = 45	563.2302m/z_3.2min, 947.2767m/z_3.64min, 340.1031m/z_3.99min, 415.1494m/z_5.09min, 663.2823m/z_6.72min, 461.1301m/z_2.57min, 455.1174m/z_3.44min, 441.1403m/z_4.66min, 627.1898m/z_3.99min, 397.2192m/z_6.83min, 643.26m/z_5.59min, 337.2043m/z_12.49min, 577.1735m/z_5min, 317.1233m/z_3.26min, 443.1915m/z_3.3min, 475.2179m/z_3.74min, 317.1026m/z_5.33min, 481.1372m/z_3.29min, 277.2167m/z_12.49min, 313.2376m/z_8.94min, 421.0732m/z_2.91min, 204.1236m/z_1.7min, 507.1571m/z_4.11min, 431.1703m/z_8.05min, 659.4715m/z_6.83min, 372.123m/z_8.35min, 327.2168m/z_6.47min, 311.2217m/z_9.26min, 529.0899m/z_8.99min, 293.2109m/z_9.69min, 259.1287m/z_2.42min, 381.1805m/z_3.94min, 593.1603m/z_4.02min, 297.2431m/z_12.02min, 221.0456m/z_4.46min, 242.1757m/z_6.64min, 393.1709m/z_11.22min, 287.2221m/z_11.9min, 311.1679m/z_10.51min, 397.0525m/z_3.84min, 283.1378m/z_8.91min, 329.1063m/z_5.71min, 369.0981m/z_6.53min, 485.1091m/z_1.62min, 432.1255m/z_2.84min, 461.2192m/z_3.52min, 375.1215m/z_3.73min, 501.1525m/z_3.77min, 539.2298m/z_4.37min, 641.2347m/z_4.64min, 466.7693m/z_4.78min, 535.106m/z_4.84min, 447.1122m/z_5.22min, 385.1159m/z_2.35min, 361.0723m/z_3.61min, 415.143m/z_4.85min	663.2823m/z_6.72min, 461.1301m/z_2.57min, 455.1174m/z_3.44min, 441.1403m/z_4.66min, 627.1898m/z_3.99min, 397.2192m/z_6.83min, 643.26m/z_5.59min, 337.2043m/z_12.49min, 577.1735m/z_5min, 317.1233m/z_3.26min, 443.1915m/z_3.3min, 475.2179m/z_3.74min, 457.2059m/z_4.37min, 441.1741m/z_4.4min, 553.1935m/z_5.14min, 263.1283m/z_6.06min, 277.2167m/z_12.49min, 313.2376m/z_8.94min, 204.1236m/z_1.7min, 373.1393m/z_4.65min, 487.1502m/z_5.44min, 377.2061m/z_6.05min, 507.1571m/z_4.11min, 217.123m/z_6.31min, 431.1703m/z_8.05min, 659.4715m/z_6.83min, 285.0797m/z_4.86min, 327.2168m/z_6.47min, 311.2217m/z_9.26min, 293.2109m/z_9.69min, 259.1287m/z_2.42min, 381.1805m/z_3.94min, 419.2319m/z_6.48min, 593.1603m/z_4.02min, 297.2431m/z_12.02min, 242.1757m/z_6.64min, 393.1709m/z_11.22min, 287.2221m/z_11.9min, 311.1679m/z_10.51min, 397.0525m/z_3.84min, 283.1378m/z_8.91min, 329.1063m/z_5.71min, 385.1159m/z_2.35min, 361.0723m/z_3.61min, 415.143m/z_4.85min
**T(200)**		
forbs<br>*p<0.001* = 23<br>*p<0.01* = 47<br>*p<0.05* = 62<br>total = 132	397.2129m/z_7.49min, 547.2386m/z_4.38min, 487.1991m/z_4.39min, 391.1278m/z_4.66min, 477.1979m/z_4.69min, 239.1284m/z_4.91min, 655.2799m/z_4.97min, 475.2169m/z_5.13min, 327.2168m/z_6.47min, 283.1909m/z_7.59min,	547.2386m/z_4.38min, 239.1284m/z_4.91min, 327.2168m/z_6.47min, 283.1909m/z_7.59min, 431.1703m/z_8.05min, 277.2167m/z_12.49min, 397.2129m/z_7.49min, 327.2169m/z_8.34min, 293.2109m/z_9.69min, 487.1812m/z_4.84min,

grasses
p<0.001 = 17
p<0.01 = 52
p<0.05 = 53
total = 122

431.1703m/z_8.05min, 277.2167m/z_12.49min,
487.1812m/z_4.84min, 295.2269m/z_10.1min,
327.2169m/z_8.34min, 293.2109m/z_9.69min,
182.989m/z_15.14min, 511.1875m/z_4.49min,
311.2217m/z_9.26min, 253.2167m/z_12.85min,
266.0188m/z_3.72min, 291.196m/z_9.42min,
265.0581m/z_4.21min,
469.1666m/z_4.27min, 329.2324m/z_6.83min,
177.0549m/z_3.37min, 239.0199m/z_3.81min,
445.2057m/z_3.4min, 417.212m/z_4.85min,
329.2324m/z_7.49min, 313.2376m/z_8.94min,
611.2253m/z_4.12min, 361.1987m/z_10.79min,
493.2279m/z_5.48min, 242.1757m/z_6.64min,
397.1813m/z_4.9min, 291.196m/z_10.14min,
563.2333m/z_3.62min, 643.26m/z_5.59min,
401.1808m/z_4.04min, 309.2038m/z_8.66min,
201.1129m/z_5.67min, 311.2219m/z_7.72min,
297.2426m/z_10.81min, 509.223m/z_3.78min,
397.2192m/z_6.83min, 311.2214m/z_9.06min,
363.2138m/z_10.35min, 295.2269m/z_11.31min,
241.0799m/z_1.39min, 803.3671m/z_4.26min,
255.1034m/z_6.19min, 317.1026m/z_5.33min,
327.2161m/z_7min, 309.2064m/z_7.25min,
239.1283m/z_4.7min, 277.0712m/z_1.4min,
447.1669m/z_5.95min, 613.2764m/z_6.08min,
379.2096m/z_9.07min, 513.1825m/z_3.41min,
381.2311m/z_13.44min, 435.2223m/z_3.89min,
299.1757m/z_9.54min, 631.1637m/z_3.93min,
537.1675m/z_4.87min, 431.192m/z_4.36min,
467.2166m/z_6.08min, 511.1694m/z_4.64min,
369.0952m/z_8.75min,
393.1758m/z_2.58min, 293.1751m/z_8.15min,
563.1449m/z_5.35min, 401.1809m/z_4.27min,
321.2095m/z_13.8min, 389.1232m/z_4.43min,
243.1235m/z_5.01min, 507.2013m/z_3.87min,
273.0959m/z_5.67min, 463.1818m/z_4.3min,
563.2179m/z_3.98min, 509.218m/z_3.25min,
313.2378m/z_8.24min, 427.1959m/z_4.52min,
615.2247m/z_4.38min, 311.0771m/z_4.13min,

295.2269m/z_10.1min, 511.1875m/z_4.49min,
311.2217m/z_9.26min, 253.2167m/z_12.85min,
266.0188m/z_3.72min, 291.196m/z_9.42min,
265.0581m/z_4.21min,
469.1666m/z_4.27min, 329.2324m/z_6.83min,
177.0549m/z_3.37min, 365.2676m/z_9.63min,
445.2057m/z_3.4min, 417.212m/z_4.85min, 329.2324m/z_7.49min,
313.2376m/z_8.94min, 611.2253m/z_4.12min,
361.1987m/z_10.79min, 303.2321m/z_13.11min,
125.0963m/z_4.9min, 493.2279m/z_5.48min,
242.1757m/z_6.64min, 397.1813m/z_4.9min,
291.196m/z_10.14min, 563.2333m/z_3.62min, 643.26m/z_5.59min,
401.1808m/z_4.04min, 309.2038m/z_8.66min,
201.1129m/z_5.67min, 311.2219m/z_7.72min,
297.2426m/z_10.81min, 509.223m/z_3.78min,
397.2192m/z_6.83min, 311.2214m/z_9.06min,
363.2138m/z_10.35min, 295.2269m/z_11.31min,
241.0799m/z_1.39min, 431.192m/z_4.36min,
803.3671m/z_4.26min, 455.317m/z_13.87min,
255.1034m/z_6.19min, 327.2161m/z_7min, 309.2064m/z_7.25min,
267.1249m/z_5.53min, 295.2272m/z_11.84min,
239.1283m/z_4.7min, 277.0712m/z_1.4min, 447.1669m/z_5.95min,
613.2764m/z_6.08min, 379.2096m/z_9.07min,
565.3362m/z_5.57min, 381.2311m/z_13.44min,
435.2223m/z_3.89min, 299.1757m/z_9.54min,
631.1637m/z_3.93min, 537.1675m/z_4.87min,
467.2166m/z_6.08min, 537.3211m/z_5.45min,
449.2105m/z_4.9min, 511.1694m/z_4.64min,
563.1449m/z_5.35min, 293.1751m/z_8.15min,
321.2095m/z_13.8min, 401.1809m/z_4.27min,
389.1232m/z_4.43min, 243.1235m/z_5.01min,
273.0959m/z_5.67min, 563.2179m/z_3.98min,
313.2378m/z_8.24min, 427.1959m/z_4.52min,
615.2247m/z_4.38min, 311.0771m/z_4.13min,
293.2111m/z_9.42min, 283.0534m/z_8.43min,
267.0871m/z_3.62min, 217.123m/z_6.31min, 309.2051m/z_8min,
611.185m/z_5.05min, 348.9199m/z_7.09min,
279.2323m/z_13.23min, 449.2022m/z_4.42min,
311.1679m/z_10.51min, 431.2638m/z_7.55min,

293.2111m/z_9.42min, 283.0534m/z_8.43min,<br>267.0871m/z_3.62min, 507.2356m/z_6.17min,<br>269.0851m/z_9.66min, 611.185m/z_5.05min,<br>348.9199m/z_7.09min, 499.181m/z_4.39min,<br>279.2323m/z_13.23min, 449.2022m/z_4.42min,<br>311.1679m/z_10.51min, 625.1776m/z_5.21min,<br>431.2638m/z_7.55min, 271.2274m/z_12.35min,<br>297.0398m/z_7.29min, 333.1368m/z_9.45min,<br>403.1956m/z_4.2min, 389.1087m/z_1.15min,<br>481.1372m/z_3.29min, 337.2043m/z_12.49min,<br>309.1949m/z_9.45min, 309.2039m/z_9.76min,<br>439.0564m/z_1.79min, 447.2224m/z_5.5min,<br>293.2111m/z_10.79min, 173.0816m/z_4.1min,<br>307.1907m/z_7.68min, 441.2519m/z_11.41min,<br>209.1179m/z_6.32min, 231.0297m/z_4.3min,<br>395.2428m/z_8.5min, 267.0661m/z_7.73min,<br>425.1805m/z_4.99min, 337.2032m/z_9.3min,<br>383.3513m/z_15min, 221.1538m/z_8.15min,<br>269.2116m/z_10.42min, 327.2166m/z_7.41min,<br>533.1656m/z_4.43min, 351.1807m/z_7.58min,<br>395.2037m/z_6.47min, 561.1753m/z_3.3min,<br>285.2065m/z_7.82min, 297.2423m/z_10.74min,<br>285.2065m/z_8.13min, 551.1765m/z_3.6min	271.2274m/z_12.35min, 333.1368m/z_9.45min,<br>403.1956m/z_4.2min, 389.1087m/z_1.15min,<br>337.2043m/z_12.49min, 309.1949m/z_9.45min,<br>309.2039m/z_9.76min, 439.0564m/z_1.79min,<br>271.2271m/z_10min, 293.2111m/z_10.79min,<br>173.0816m/z_4.1min, 307.1907m/z_7.68min,<br>441.2519m/z_11.41min, 209.1179m/z_6.32min,<br>395.2428m/z_8.5min, 267.0661m/z_7.73min,<br>425.1805m/z_4.99min, 337.2032m/z_9.3min, 383.3513m/z_15min,<br>221.1538m/z_8.15min, 269.2116m/z_10.42min,<br>327.2166m/z_7.41min, 533.1656m/z_4.43min,<br>351.1807m/z_7.58min, 395.2037m/z_6.47min,<br>561.1753m/z_3.3min, 285.2065m/z_7.82min,<br>297.2423m/z_10.74min, 285.2065m/z_8.13min,<br>551.1765m/z_3.6min

c) Percentage of correlated compounds per predictor

Metabolite polarity	Growth form	Predictor	LUI	soil	climate	LNH	Total
**Semi-polar**	forb	significantly affected compounds [%]	19.37	52.82	30.28	34.15	65.14
	grass		23.94	52.11	30.28	34.86	69.72
**polar**	forb	significantly affected compounds [%]	6.05	12.20	8.27	6.15	21.90
	grass		4.68	9.94	7.14	4.68	17.49

Supplementary Table 6: Putative classification of the significantly species specific semi-polar metabolites. The table contains no. of metabolites, species, retention time (RT), m/z value, p value for significance of occurrence in single species, type of adduct, putative elemental composition, putative classification, observed neutral losses and fragment ions upon CID. The identifier ions (see Supplementary Table 7) were marked in bold. * = precursor ion in addition to fragment ions and neutral losses used for annotation of features. All measurements were obtained in negative ionisation mode.

No.	Species	RT [min]	m/z	p -value	Type of adduct	Putative Elemental composition	Putative Class	Fragment ions and neutral losses detected in CID (identifier ions in bold)	
								**neutral losses**	**fragment ion**
1	*A. millfefolium*	2.6	409.0444	$1.62x10^{-14}$	[M-H]-	$C_{14}H_{18}O_{12}S$	Unclassified, sulfate/phosphate residue[1]	212.0004, 198.054, 227.0276, 312.0816, 210.9981, 218.9993, 312.1053, 145.0984, 284.0586, 262.9787, 339.2814, 208.0553, 197.0461, 32.1573, 50.9537	197.0453, 210.9946, 182.0217, **96.9568**, 198.0467, 190.0527, 96.9336, 263.9458, 124.9758, 146.063, 69.7617, 200.9865, 74.724, 167.0497, 185.071

2	*A. millfefolium*	3.12	619.1862	$1.05x10^{-08}$	[M-H]-	C26H36O17	Hydroxycinnamic acid, glycosylated	474.1642, 502.1571, 278.0764, 506.1669, 473.1586, 500.1504, 488.145, 440.1207, 476.1574, 357.1224, 296.1033	**145.0209**, 117.0335, 341.1108, **113.0231**, 146.0322, 119.0359, 131.0378, **179.0672**, 143.0333, 262.0666, **323.0905**
3	*A. millfefolium*	3.46	515.139	$1.11x10^{-14}$	[M-H]-	C22H28O14	Phenylpropanoid, Coumarin derivative[1]	309.1171, 294.0955, 324.1371, 308.1123	206.0189, 221.0502, 190.996, **207.0349**
4	*A. millfefolium*	3.72	577.2099	$6.40x10^{-12}$			Unclassified		
5	*A. millfefolium*	3.94	607.1697	$1.12x10^{-07}$	[M-H]-	C20H36N2O17S	Unclassified	382.1073, 397.132, 381.1031	225.0533, 210.031, 226.0558, 223.0388
6	*A. millfefolium*	4.06	581.1858	$1.44x10^{-06}$			Unclassified		
7	*A. millfefolium*	4.46	221.0456	$1.12x10^{-07}$			Unclassified		
8	*A. millfefolium*	4.6	475.2175	$1.10x10^{-06}$			Unclassified		

9	*A. millfefolium*	4.77	509.2194	$6.54x10^{-15}$	[M-H]-	C22H38O13	Glycoside, Hydroxycarbonic acid residue	178.0517, 276.154, 396.1957, 360.1758, 318.1651, 378.185, 348.1771, 177.0455, 384.1958, 46.0095, 366.1882, 408.1954, 258.148	331.1765, 233.0658, **113.0231**, 149.0453, **191.0543**, 131.0378, 161.0446, 332.1799, 125.0251, 463.2163, 143.0333, **101.0229**, 251.0699
10	*A. millfefolium*	4.82	503.1812	$5.56x10^{-17}$	[M-H]-	C22H32O13	Glycoside	355.1187, 340.095, 378.1496, 390.151, 354.1195, 270.1076	148.043, **163.0702**, 125.0251, **113.023**1, 149.0602, 233.0658
11	*A. millfefolium*	4.84	517.1912	$3.15x10^{-22}$	[M-H]-	C22H32O11	Glycoside[1]	369.1435, 354.1195, 270.1182, 374.1608	148.043, **163.0702**, 247.0788, 143.0333
12	*A. millfefolium*	4.89	547.2022	$4.70x10^{-24}$	[M-H]-	C24H36O14	Hydroxycinnamic acid	354.1195, 369.1435, 422.1791, 384.1565, 353.1128, 300.1209	193.0852, 178.061, 125.0251, **163.0379**, 194.0828, 247.0788, **179.0672**

13	*A. millfefolium*	4.9	689.2999	$2.15x10^{-16}$	[M-H]-	C30H48N3O15	Unclassified	502.2034, 564.2056, 501.2007	187.1029, 125.0948, 188.1097
14	*A. millfefolium*	5.02	487.1807	$1.30x10^{-16}$	[M-H]-	C22H32O12	Glycoside[1]	374.1504, 372.1714	113.0231, 115.0009, *116.0109*, ***175.0257***
15	*A. millfefolium*	5.13	469.1704	$1.34x10^{-18}$	[M-H]-	C17H30N2O13	Unclassified	252.0464, 208.0553, 251.0431	217.1273, 261.1183, 218.127
16	*A. millfefolium*	5.37	452.1914	$1.26x10^{-14}$	[M-H]-	C23H33O7S	Glycoside, sulfated/phosphorylated	355.2365, 93.7395, 339.1698, 185.1303, 272.2962, 240.113, 232.1912, 322.1442, 99.0917, 172.1712, 279.1086, 119.1266, 197.1374, 80.4742, 133.0596	**96.9568**, **113.0231**, 358.4508, 267.0669, 179.8941, 212.0831, 130.0557, 219.9961, 173.0896, 280.0169, 353.0986, 333.0583, 255.0523, 157.0404, 223.0388
17	*A. millfefolium*	5.39	505.225	$3.31x10^{-12}$	[M-H]-	C21H42O9S	Glycoside, sulfated, hydroxycarbonic acid	392.2027, 272.1498, 380.2012, 344.178, 346.1952, 404.2041, 314.1683, 354.1195,	**113.0231**, 233.0658, 125.0251, **161.0446**, 159.0277, **101.0229**, **191.0543**, 151.1072,

								362.2023, 342.1676, 376.2111	143.0333, 163.0568, 129.0167, *119.0359*
18	*A. millfefolium*	5.61	493.2278	1.42x10^{-06}	[M-H]-	C22H38O12	Glycoside[1]	178.0517, 46.0095, 332.1813, 177.0455	315.1787, 447.221, **161.0446**, 316.1873
19	*A. millfefolium*	5.88	273.1701	7.27x10^{-10}	[M-H]-	C9H26N2O7	Unclassified	158.1232, 18.0104, 144.1132, 128.1194, 80.009, 134.0531, 146.0581, 98.0194, 118.0644, 36.0189, 162.1357	273.1682, 115.039, 255.1592, 129.0568, 145.0603, 193.1583, 139.1142, 127.1175, 175.1505, 155.1104, 237.1528, 111.0432
20	*A. millfefolium*	5.91	177.0552	1.30x10^{-10}			Unclassified		
21	*A. millfefolium*	6.1	508.2429	1.30x10^{-10}	[M-H]-	C49H78O22	Glycoside	395.2192, 100.9131, 299.1167, 255.1396, 301.1845, 323.1252	**113.0231**, 407.3307, 209.1159, 253.1134, **207.0689**, 185.1131
22	*A. millfefolium*	8.77	645.4327	1.10x10^{-06}	[M-H]-	C36H60N3O7	Unclassified	452.3543, 334.2151, 438.3361, 444.3181, 352.2271,	193.0852, 311.228, 207.0908, 201.1212, 293.2057,

								370.2336, 474.3344, 460.3196	275.2032, 171.1069, 185.1131
23	*A. millfefolium*	8.89	286.1802	1.26×10^{-14}	[M-H]-	C18H25NO2	Unclassified	120.0572, 150.1077	286.1903, 166.1256, 136.0773
24	*G. mollugo*	2.58	393.1758	5.26×10^{-15}	[M-H]-	C16H28O8	Flavonoid	164.255, 283.5116, 43.1881, 292.5814, 242.1361, 245.0963, 256.1353, 192.1184, 204.23, 183.1512, 263.3766, 216.165, 103.2068, 154.241, 222.5841	228.9224, 109.6651, 349.9885, 100.5952, **151.0413**, **137.0259,** 201.0544, 148.0824, 188.9466, 210.031, 129.8001, 289.9826, […], 170.5926, 209.1159
25	*G. mollugo*	3.01	519.1702	5.86×10^{-13}	[M-H]-	C29H28O9	Hydroxycinnamic acid	340.095, 398.1402, 355.1187, 373.1277, 354.1518, 358.1025, 397.132	**179.0672**, 121.0303, **164.0454**, 146.0322, 165.0216, 161.0601, 122.0342, **113.0231**

26	*G. mollugo*	3.05	391.1576	$1.48x10^{-13}$	[M-H]-	C17H28O10	Hydroxycinnamic acid, glycosylated	198.1164, 190.171, 168.1308, 272.1252, 280.0771, 76.1027, 131.0851, 160.0235, 62.0868, 75.0328, 343.5843, 189.0801, 278.1277, 174.0308, 144.1132	193.0497, 200.9865, 223.0388, 119.0359, 111.0818, 315.0499, 260.0687, 231.1328, 47.5766, 316.1281, 329.0818, **113.0231**, […], 217.1273, 199.017, **178.0276**
27	*G. mollugo*	3.09	165.0192	$7.81x10^{-14}$	[M-H]-	C6H4N3O3	Unclassified, aromatic acid[1]	**43.989**, 23.0548	121.0303, 141.9658
28	*G. mollugo*	3.36	391.1601	$8.05x10^{-16}$	[M-H]-	C24H26NO2S	Unclassified	194.1111, 193.1121, 238.1003, 189.1605, 190.171, 136.0447, 154.9775, 156.0962, 209.1341, 314.9471, 163.0483, 239.2411, 216.0961, 37.0474, 289.9756	197.0453, 198.0467, 153.0575, 202.0021, 200.9865, 255.1176, 236.1812, 235.0632, 76.2137, 182.0217, 228.1068, 151.9285, 354.1048, 101.1812, 175.0619

29	*G. mollugo*	3.39	377.1791	$1.83x10^{-08}$	[M-H]-	C17H30O9	Flavonoid, Kaempferol derivative[1]	311.2672, 78.0308, 146.1246, 230.1554, 162.1357, 176.1981, 151.1389, 176.1042, 161.9119, 229.146, 222.4788, 186.1652, 103.1389, 226.2397, 183.1034	65.9136, 299.1498, 231.0557, 147.0334, 215.0327, 200.9865, 226.0323, 201.0772, 148.043, 215.2684, 154.7016, 191.0163, 274.0467, 150.9364, 136.0447, 194.0828, […], **255.0294**
30	*G. mollugo*	3.4	581.1875	$2.57x10^{-10}$	[M-H]-	C30H32NO11	Unclassified	369.1011, 368.097, 381.1328	212.0831, 213.0898, 200.0544
31	*G. mollugo*	3.57	507.2064	$4.60x10^{-11}$			Unclassified		
32	*G. mollugo*	3.73	401.111	$9.32x10^{-11}$			Unclassified		
33	*G. mollugo*	3.93	527.136	$9.38x10^{-19}$	[M-H]-	C21H26N3O13	Unclassified	325.1092, 324.1021, 314.0802	202.0256, 203.0356, 213.0539

34	*G. mollugo*	4.13	377.1808	$1.49x10^{-15}$	[M-H]-	C18H26N4O5	Unclassified	160.0532, 120.1355, 89.0959, 70.1055, 133.0881, 161.1321, 190.1113, 146.0843, 265.0856, 162.0547, 132.0699, 176.1981, 258.1007, 88.0964, 183.0566	217.1273, 257.0462, 288.0859, 244.0925, 307.0801, 216.0419, 231.1006, 187.0653, 112.096, 215.124, 245.1126, […], 93.0304, […], 205.1235
35	*G. mollugo*	4.15	527.104	$4.69x10^{-14}$	[M-H]-	C23H20N4O11	Unclassified	325.0858, 324.0765	202.0256, 203.0356
36	*G. mollugo*	4.37	389.1236	$1.24x10^{-12}$	[M-H]-	C20H22O8	Hydroxycinnamic acid	224.0607, 196.0708, 239.0878, 107.0383, 255.0854, 211.087, 209.0788, 194.0539, 150.0549, 267.0877, 225.0701, 139.0619, 195.0678, 254.0869, 135.0641	165.0558, **193.0497,** 150.038, 282.0876, 134.0357, 178.0276, 180.042, 195.0709, 239.0709, 122.0342, **164.0454**, 250.0566, 194.0551, **135.0437**, 254.0558, 121.0303, 149.0142, […],**151.0413**

37	*G. mollugo*	4.37	389.1236	$1.24x10^{-12}$	[M-H]-	C20H22O8	Hydroxycinnamic acid	224.0723, 255.0854, 196.0708, 239.0878, 107.0383, 211.0984, 240.0705, 150.0549, 268.096, 106.0398, 122.0631, 195.0678, 139.0619, 209.0788, 225.0701	165.0558, 134.0357, **193.0497**, 150.038, 282.0876, **178.0276**, 149.0602, 239.0709, 121.0303, 283.1, 267.0669, 194.0551, 250.0566, 180.042, **164.0454**
38	*G. mollugo*	4.98	507.2066	$1.74x10^{-07}$	[M-H]-, [M-H-H2O]-	C22H36O13	Flavonoid, glycosylated	305.1819, 270.1357, 223.1699, 208.1579, 305.1145, 346.166, 270.2334, 360.1568, 140.1467, 276.1678, 296.0789, 284.2023, 268.167, 376.1676, 149.2726	202.0256, 237.0561, **284.0313**, 299.0483, 202.0964, **161.0446**, 147.0459, 236.9701, 211.1362, 231.0299, 367.0587, 239.0357, […], 357.9329

39	*G. mollugo*	5.05	313.0357	1.26x10^{-06}	[M-H]-	C14H8N3O6	Unclassified, imin	87.0185, **43.9987**, **59.0156**	226.0323, 269.044, 254.0167, 227.0341
40	*G. mollugo*	5.15	577.1552	2.01x10^{-14}	[M-H]-	C27H30O14	Hydroxycinnamic acid, glycosylated	356.0863325.1092 324.1021340.0954 16.1043464.13224 46.1132	221.065, 252.0408, 253.0499, 237.0561, **161.0446**, **113.0231**, 236.0486, 131.0378, *222.0664*, ***101.0229***, *224.0455*, ***179.0672***, ***193.0497***
41	*G. mollugo*	5.2	507.2071	9.38x10^{-19}	[M-H]-	C17H38N3O12S	Unclassified	305.1819, 304.1743, 338.0845, 306.1887, 252.1182, 58.9521, 375.1782, 315.1467, 376.2111, 289.1956, 310.1393, 219.0976, 192.1629, 294.1511, 379.193	202.0256, 203.0356, 169.1139, 201.0119, 255.0892, 448.2562, 132.0295, 192.0649, 131.0014, 197.0713, 218.0128, 213.0539, 58.0232, 315.0499, 128.0193

42	*G. mollugo*	5.49	547.1437	$1.40x10^{-17}$	[M-H]-	C24H26N3O12	Unclassified	295.1038, 294.0955, 297.0948, 310.0917, 278.1277, 323.1025, 282.1465, 282.0569, 334.062, 309.0735, 292.1415, 222.0919, 244.1754, 328.0758, 308.0726	252.0408, 253.0499, 250.0566, 237.0561, 269.0075, 224.0455, 264.9976, 265.092, 213.0898, 238.0726, 255.0025, 325.0584, 219.072, 302.9707, 239.0709
43	*G. mollugo*	5.74	419.134	$1.82x10^{-13}$	[M-H]-	C21H24O9	Flavonoid[1]	207.0486, 163.0633, 206.04, 192.0948, 213.0769	212.0831, 256.0748, 213.0898, 227.0341, 206.0671, 200.0544, **255.0294**
44	*G. mollugo*	5.79	415.1013	$1.88x10^{-08}$	[M-H]-	C21H20O9	Flavonoid[1]	120.0772, 94.0907, 178.0425, 163.0633, 160.0735, 119.072, 161.0716	295.0236, 321.0029, 237.0561, 252.0408, **255.0294**, 254.0316, 209.1159, 268.0375, 253.0499

45	*G. mollugo*	5.89	669.2385	$2.02x10^{-12}$	[M-H]-	C18H44N2O21	Unclassified	385.1305, 384.1321, 370.1145	284.1041, 285.1101, 299.1222
46	*G. mollugo*	6.03	337.1075	$1.49x10^{-11}$	[M-H]-	C20H20O6	Polyketide[1]	30.0455, 58.0429, 46.0391, 29.0407, 15.0225, 31.0211, 45.0353, 71.0265, 86.0358, 43.0172, **42.0168**	307.0609, 279.0696, 291.0655, 322.0769, 306.0859, 292.0747, 266.083, 251.0699, 294.0872, 295.1001
47	*G. mollugo*	6.08	253.0504	$1.73x10^{-21}$	[M-H]-	C20H20O6	Polyketide[1]	43.0172, **42.0168**	210.031, 253.0499, *211.0398, 223.0388*
48	*G. mollugo*	6.13	269.0452	$2.57x10^{-10}$			Unclassified		
49	*G. mollugo*	6.25	515.1521	$9.38x10^{-19}$	[M-H]-	C14H31N5O13	Unclassified, methoxylated	231.0401, 286.0994, 290.0612, 308.0726, 230.0483, 274.095	284.1041, 229.0505, 225.0892, 207.0908, 285.1101, 241.0482
50	*G. mollugo*	6.44	253.0508	$2.38x10^{-11}$	[M-H]-	C15H10O4	Polyketide[1]	43.0172, **42.0168**	210.031, 253.0499, 211.0398
51	*G. mollugo*	6.45	267.0298	$1.51x10^{-09}$	[M-H]-	C6H14N5OS3	Unclassified	**43.989**, 71.9859, 42.9854	223.0388, 195.0451, 224.0455

52	*G. mollugo*	6.8	285.0405	9.11×10^{-08}	[M-H]-	C15H10O6	Flavonoid	44.9997, **46.0007**, 72.9917, **43.9987**, **27.9962**, 88.9936, 15.0225	**285.0426**, 240.0384, **239.0357**, 212.0454, 241.0482, 257.0462, 196.0531, 270.0217
53	*G. mollugo*	6.81	489.1752	5.86×10^{-13}	[M-H]-	C12H34N4O14S	Unclassified	205.0691, 260.13, 264.0868, 282.0933, 248.1248, 204.066, 263.0795, 220.0928, 281.0887	284.1041, 229.0505, 225.0892, 207.0908, 241.0482, 285.1101, 226.0911, 269.0837, 208.0806
54	*G. mollugo*	6.9	283.0257	1.83×10^{-08}			Unclassified		
55	*G. mollugo*	7.17	381.0621	9.11×10^{-08}	[M-H]-	C9H22N2O10S2	Unclassified	**43.989**, 42.9854, 61.9988, 71.9859, 18.0104, 99.0465, 139.0329, 87.9783, 70.9819, 127.041, 149.9638, 94.8676, 143.0218, 71.897, 18.1036	337.0782, 338.0735, 319.0581, 309.0732, 363.0423, 282.0119, 242.0326, 293.077, 310.0785, 254.0167, 231.1006, 286.1903, 238.0404, 309.1665, 362.9718

56	*G. mollugo*	7.17	491.1549	1.14x10^{-20}	[M-H]-	C23H26O9	Hydroxycinnamic acid[1]	208.0553, 267.0696, 268.0747, 209.0668, 240.0845, 282.0933, 266.0616, 284.0706, 207.0486	283.1, 224.0745, **223.0655**, 282.0876, 251.0699, 209.0613, 225.0892, 207.0908, 284.1041
57	*G. mollugo*	7.27	239.0351	6.32x10^{-16}	[M-H]-	C6H14N2O7S	Unclassified	**27.9962**, 29.0025, 81.0227, 77.9057, 26.991, 81.4106, 42.9854	211.0398, 210.031, 238.0273, 158.0214, 161.1349, 238.1167
58	*G. mollugo*	7.27	501.0589	2.20x10^{-10}	[M-H]-	C20H14N4O12	Terpene, Sesqueterpene[1]	262.0255, 261.0287	**239.0357**, 240.0384
59	*G. mollugo*	7.63	299.0537	2.37x10^{-11}	[M-H]-	C16H12O6	Flavonoid, Kaempferol derivative	15.0225, **44.0226**, 14.0188	**284.0313**, **255.0294**, **285.0426**
60	*G. mollugo*	7.73	267.0661	4.26x10^{-22}	[M-H]-	C16H12O4	Polyketide	43.0172, 15.0225, **42.0168**	224.0455, 267.0669, 252.0408, 225.0533
61	*G. mollugo*	7.74	224.047	1.88x10^{-08}			Unclassified		
62	*G. mollugo*	8.57	253.0481	2.57x10^{-10}			Unclassified		
63	*G. mollugo*	9.67	283.0247	1.38x10^{-13}	[M-H]-	C16H4N4O2	Terpene, Sesqueterpene	**43.989**, 87.9783, 42.9854, 71.9859	**239.0357**, 195.0451, 240.0384, 211.0398

64	*G. mollugo*	9.84	367.1184	$9.11x10^{-08}$	[M-H]-	C21H20O6	Unclassified	70.0755, 58.0759, 57.074, 15.0225, 69.0738	297.0412, 309.0401, 310.0321, 352.0935, 298.047
65	*G. verum*	8.24	397.0925	1.02x10-06	[M-H]-	C21H18O8	Flavonoid	102.0702, 114.0704, 76.1027, **59.0156**, 101.0738, 18.0104	295.0236, **283.0318,** 321.0029, 338.0735, 379.0867, **284.0313**
66	*Galium spp.*	2.03	417.1033	$4.12x10^{-08}$	[M-H]-	C17H22O12	Hydroxycinnamic acid	224.052, 213.0956, 256.0736, 284.0706, 294.0542, 268.0376, 198.0706, 290.0612, 212.0888, 206.04, 223.0476, 316.078, 266.0616, 197.0673, 255.074	**193.0497**, 204.0101, **161.0265**, 133.0335, 123.0439, 149.0602, 219.0272, 127.0411, 205.0178, 211.0615, 194.0551, **101.0229**, **151.0413**, 220.0415, 162.035
67	*Galium spp.*	2.67	449.1295	$6.13x10^{-31}$	[M-H]-	C17H24O11	Hydroxycinnamic acid, glycosylated	310.0799, 208.0553, 240.0705, 348.0897, 206.04, 258.0849, 322.0782, 238.0638, 254.0532,	139.0405, 241.072, 209.0456, **101.0229**, 243.0919, 191.0368, 127.0411, 211.0615, 195.0709,

								226.0665, 324.1021, 328.0912, 338.1138, 309.0735	**223.0655**, 125.0251, 121.0303, 111.0098
68	*Galium spp.*	2.97	355.1012	$7.48x10^{-14}$	[M-H]-	C16H20O9	Hydroxycinnamic acid	162.0547, 221.062, 206.04, 177.0753, 220.0571, 188.062	**193.0497**, 134.0357, 149.0602, **178.0276**, **135.0437**, 167.0339
69	*Galium spp.*	3.25	509.218	$2.80x10^{-12}$			Unclassified		
70	*Galium spp.*	3.4	355.1026	$4.79x10^{-09}$	[M-H]-	C23H16O4	Hydroxycinnamic acid, glycosylated	**162.0418**, 221.062, 177.0753, 206.04, 161.0481, 241.0424, 59.9545, 247.0824	**193.0497**, 134.0357, **178.0276**, 149.0602, 194.0551, 114.0562, 295.1493, 108.0202, 249.0584, […], 151.1072
71	*Galium spp.*	3.8	577.2098	$2.61x10^{-35}$	[M-H]-	C17H40NO20	Terpene, Sesqueterpene[1]	464.2245, 338.1698, 339.1795, 275.9658, 460.2774, 338.097, 378.167, 143.0172, 361.1723, 380.1574,	112.9827, **239.0357**, 238.0273, 301.245, 116.9292, 239.1014, 199.043, 216.0419, 534.193, 197.0453, 170.8584, 335.089,

								406.3482, 242.1173, 380.078, 66.462, 643.989	197.1306, 510.744, 153.0575
72	*Galium spp.*	3.82	499.1984	$4.79x10^{-09}$	[M-H]-	C13H34N5O15	Unclassified	258.1194, 281.1192, 440.1816, 168.0139, 128.2059, 127.9336, 259.9124, 404.9913, 298.1503, 355.1484, 258.9818, 254.841, 277.1299, 167.7446, 337.269	241.072, 218.0722, 59.0104, 331.1765, 370.9866, 94.1978, 371.2544, 239.2731, 201.0544, 144.0525, 222.0664, 240.2049, 244.3515, 497.2539, 161.9298
73	*Galium spp.*	4.09	509.223	$5.55x10^{-11}$	[M-H]-	C22H38O13	Glycoside	178.0425, 348.1771, 276.154, 177.0455, 396.1957, **46.0007**	331.1765, **161.0446**, 233.0658, 332.1799, **113.0231**, 463.2163, *149.0453*

74	*Galium spp.*	4.31	521.2021	$2.08x10^{-13}$	[M-H]-	C26H34O11	Hydroxycinnamic acid	192.0659, 346.1264, 191.0724, 322.1811, 282.1649, 299.1693, 318.1812, 319.2001, 319.1666, 251.0431, 402.1568, 256.3123, 185.0876, 231.7082, 306.1406	329.1413, 175.0776, 330.1251, 199.017, 239.0357, […], **207.0689**, […], **151.0413**, […], **179.0368**
75	*Galium spp.*	4.42	655.2235	$2.04x10^{-35}$	[M-H]-	C16H43N4O18P	Unclassified	371.1161, 370.1145, 277.1299	284.1041, 285.1101, 378.0999
76	*Galium spp.*	4.53	547.2385	$3.86x10^{-12}$	[M-H]-	C25H40O13	Glycoside	434.2131, 338.0845, 337.0776, 176.0262, 118.0197, 372.2099	**113.0231**, 209.1516, 371.2055, 429.2187, **175.0257**
77	*Galium spp.*	4.77	535.1165	$1.19x10^{-14}$	[M-H]-	C12H32N4O13S3	Unclassified	333.0777, 332.0725, 322.0542	202.0256, 203.0356, 213.0539
78	*Galium spp.*	4.78	467.122	$2.26x10^{-16}$	[M-H]-	C11H26N5O13S	Unclassified	265.0953, 264.0868	202.0256, 203.0356

79	*Galium spp.*	4.95	563.1403	$5.13x10^{-34}$	[M-H]-	C14H36N4O13S3	Unclassified	312.1053, 294.0955, 311.1003, 326.0809, 298.0884, 293.0893	251.0354, 269.044, 252.0408, 237.0561, 265.051
80	*Galium spp.*	5.01	533.1317	$4.79x10^{-09}$	[M-H]-	C12H32N5O14S2	Terpene, Sesqueterpene[1]	294.0955, 293.0893	**239.0357**, 240.0384
81	*Galium spp.*	5.07	625.1763	$1.59x10^{-19}$	[M-H]-	C25H36N6O7P2S	Unclassified	369.1011, 413.0935, 412.0863, 368.097	256.0748, 212.0831, 213.0898, 257.0826, *227.0341*
82	*Galium spp.*	5.16	609.1806	$3.99x10^{-10}$	[M-H]-	C28H34O15	Glycoside	488.145, 340.1301, 478.1305, 460.1193, 342.1139, 358.1453, 496.161, 418.1303, 487.1496, 495.1459, 402.1869, 353.1021, 356.0971, 338.0845, 381.1031	121.0303, 269.044, 131.0378, 149.0602, 267.0669, 251.0354, ***113.0231,*** *[…], 271.1001, […], 97.0278, […],* ***161.0446***
83	*Galium spp.*	5.23	299.0213	$7.46x10^{-19}$	[M-H]-	C15H8O7	Flavonoid	**43.9987**, 18.0104, 42.9854, 17.0123, 29.9765	**255.0294**, 281.0195, 256.0349, 282.0119, 269.044

84	*Galium spp.*	5.29	359.1494	$1.49x10^{-23}$	[M-H]-	C20H24O6	Terpene / Hydroxycinnamic acid	184.072, 199.0918, 167.0673, 30.0082, 181.0848, 166.0672, 182.0937, 151.0681, 108.1096	175.0776, 160.051, 192.084, 329.1413, 178.061, 193.0852, 177.0541, 208.0806, 251.0354, […], *137.0619*, *159.0451*, ***239.0357***, *281.0676*, ***179.0672***
85	*Galium spp.*	5.29	595.1663	$3.17x10^{-24}$			Unclassified		
86	*Galium spp.*	5.38	275.0919	$1.09x10^{-32}$	[M-H]-	C15H16O5	Unclassified	88.0516, 87.0518, 116.0505, 18.0104	187.0368, 188.0465
87	*Galium spp.*	5.47	595.1663	$2.93x10^{-36}$	[M-H]-	C33H28N2O9	Unclassified	339.0913, 383.0826, 338.0845, 368.1348	256.0748, 212.0831, 257.0826, 227.0341
88	*Galium spp.*	5.62	431.0977	$8.50x10^{-11}$	[M-H]-	C21H20O10	Hydroxycinnamic acid	266.0421, 238.0464, 282.0413, 297.0632, 324.0476, 284.0586, 265.0419	165.0558, **193.0497**, 149.0602, 134.0357, 107.0499, 147.0459, *112.9827*, ***178.0276***
89	*Galium spp.*	5.73	463.1281	$9.23x10^{-32}$	[M-H]-	C20H22N3O10	Unclassified	207.0486, 251.0431, 250.0348, 206.04, 236.0892	256.0748, 212.0831, 213.0898, 257.0826, 227.0341

90	*Galium spp.*	5.84	547.1453	$6.99x10^{-23}$	[M-H]-	C26H28O13	Unclassified	294.0955, 293.0893	253.0499, 254.0558
91	*Galium spp.*	6.17	653.2065	$1.75x10^{-13}$	[M-H]-	C25H38N2O18	Unclassified	370.1145, 369.1113	283.1, 284.1041
92	*Galium spp.*	6.21	239.0349	$7.46x10^{-19}$	[M-H]-	C15H4N4	Terpene, Sesqueterpene	**27.9962**, **43.989**, 26.991, 29.0025	**239.0357**, 211.0398, 195.0451
93	*Galium spp.*	6.24	447.1654	$1.18x10^{-34}$	[M-H]-	C20H27N5O5P	Unclassified	163.0633, 222.0789, 218.1138, 240.0845, 206.122, 162.0547	284.1041, 225.0892, 229.0505, 207.0908, 241.0482, 285.1101
94	*Galium spp.*	6.6	267.0297	$6.76x10^{-25}$	[M-H]-	C15H8O5	Unclassified		
95	*Galium spp.*	6.92	269.0454	$9.75x10^{-29}$	[M-H]-	C15H10O5	Unclassified	18.0104, 17.0123	251.0354, 252.0408
96	*Galium spp.*	7.02	253.0503	$1.39x10^{-27}$			Unclassified		
97	*Galium spp.*	7.2	313.0709	$4.12x10^{-08}$	[M-H]-	C17H14O6	Flavonoid, Kaempferol derivative	**30.0455**, 15.0225, 58.0429, 29.0407	**283.0318**, 298.047, **255.0294**, **284.0313**, 313.0576
98	*Galium spp.*	7.29	447.1655	$3.07x10^{-26}$	[M-H]-	C24H24N4O5	Unclassified	163.0633, 218.1138, 240.0845, 222.0789, 206.1074, 162.0547, 239.075	284.1041, 229.0505, 207.0908, 225.0892, 241.0482, 285.1101, 208.0806

99	*Galium spp.*	7.57	311.0559	$3.99x10^{-10}$	[M-H]-	C17H12O6	Polyketide[1]	43.0172, **42.0168**, 73.0329	268.0375, 311.0516, 269.044, 238.0273
100	*Galium spp.*	7.72	269.0483	$6.25x10^{-13}$	[M-H]-	C16H6N4O	Unclassified, imin, aliphatic acid	15.0225, 43.989, **46.0007**, **27.9962**, **43.0172**, 18.0104, 44.9997, **59.0156**, 58.0101, 71.9859, 29.0769, 72.9917, 29.0025, 26.991, 73.9989	254.0167, 225.0533, 223.0388, 241.0482, 226.0323, 251.0354, 224.0455, 210.031, 211.0398, 197.0573, 239.9687, 196.0531, 240.0608, 242.0578, 267.0371
101	*Galium spp.*	7.85	269.0458	$2.79x10^{-32}$			Unclassified		
102	*Galium spp.*	7.94	239.0351	$1.09x10^{-32}$			Unclassified		
103	*Galium spp.*	8.01	255.0302	$3.62x10^{-16}$	[M-H]-	C9H8N2O7	Unclassified	255.0294, 227.0341	-27.9962
104	*Galium spp.*	8.75	232.0375	$7.96x10^{-15}$	[M-H]-	C12H9O5	Unclassified	29.0025, 124.0171, 89.1289, 88.0516, 90.2506, 111.4848, 134.0749, 131.6607, 153.5161,	203.0356, 108.0202, 142.9091, 231.0299, 231.1478, 143.9826, 141.7874, 120.5484, 97.9631,

								69.4696, 174.1558, 149.4254, 40.7212, 79.4318, 174.6441	100.3686, 78.5219, 57.876, 162.5684, 82.6126, 57.4019
105	*Galium spp.*	8.75	301.1078	$4.86x10^{-30}$	[M-H]-	C15H16N3O4	Unclassified	101.0954, 113.0963, 69.0738	200.0144, 188.0014, 232.0384
106	*Galium spp.*	9.64	267.0304	$3.28x10^{-22}$	[M-H]-	C16H4N4O	Terpene, Sesqueterpene	**27.9962**, 55.9997, 71.9859, 26.991	**239.0357**, 211.0398, 195.0451, 267.0371, 240.0384
107	*P. lanceolata*	1.08	381.0951	$5.02x10^{-11}$	[M-H]-	C13H34S6	Unclassified, sulfate/phosphate residue[1]	140.0882, 284.1351	241.0025, **96.9568**
108	*P. lanceolata*	1.09	391.124	$1.35x10^{-18}$	[M+HCOOH-H]-, [M-H]-	C15H22O9	Hydroxycinnamic acid, glycosylated	226.0665, 252.082, 254.0657, 208.0553, 270.0957, 272.0794, 244.0786, 182.0774, 256.0736, 238.0638, 278.0952	165.0558, 139.0405, 137.0619, 183.0547, 121.0303, **119.0474**, 147.0459, 209.0456, **135.0437**, 153.0575, **113.0231**
109	*P. lanceolata*	1.44	409.0453	$1.54x10^{-12}$	[M-H]-	C13H19N2O9PS	Unclassified, sulfate/phosphate residue[1]	168.0443, 312.0922	241.0025, **96.9568**

110	*P. lanceolata*	1.79	439.0564	6.60×10^{-12}	[M-H]-	C14H21N2O10PS	Unclassified, sulfate/phosphate residue[1]	198.054, 342.0947	241.0025, **96.9568**
111	*P. lanceolata*	2.01	373.113	2.81×10^{-13}	[M-H]-	C16H22O10	Unclassified	206.0835, 250.074, 265.0953, 221.0989	167.0339, 123.0439, 108.0202, 152.0105, *149.0602*
112	*P. lanceolata*	2.48	463.1445	2.62×10^{-07}			Unclassified		
113	*P. lanceolata*	2.95	351.0414	9.20×10^{-13}	[M-H]-	C13H12N4O6S	Unclassified	153.9984, 169.0232, 152.9927	197.0453, 182.0217, 198.0467
114	*P. lanceolata*	3.13	433.1335	8.67×10^{-22}	[M-H]-	C16H24N3O11	Unclassified	236.0892, 251.1142, 295.1038, 235.0862	197.0453, 182.0217, 138.0261, 198.0467

115	*P. lanceolata*	3.14	497.1303	$3.45x10^{-09}$	[M-H]-	C22H26O13	Hydroxycinnamic acid	344.1063, 302.1003, 242.0719, 334.0936, 300.0852, 200.0688, 256.1095, 388.097, 362.0796, 360.0663, 332.1016, 259.0787, 278.026, 237.9732, 171.9826	153.0188, 195.0281, 255.0523, **163.0379**, 197.0453, 297.065, 241.0208, 109.0304, **135.0437**, 137.0619, 165.0216, 259.1539, […], **151.0413**, […], 259.0083, […], *313.1123, 305.1755*
116	*P. lanceolata*	3.2	563.2302	$3.01x10^{-08}$	[M-H]-	C32H36O9	Glycoside	450.2024, 358.1025, 356.0863, 440.1816, 396.1957, 374.1001, 388.203, 270.2028, 352.2007, 404.1653, 366.1882, 357.2167, 370.1786, 329.1172, 516.9181	1**13.0231**, 205.1235, 207.1389, 123.0439, 167.0339, 206.1272, 189.1289, **175.0257**, 153.0918, […], 223.1375, […], **161.0446**, […], **101.0229**

117	*P. lanceolata*	3.34	379.1593	6.06x10^{-11}	[M+Na-H]-, [M+HCOOH-H]-	C18H23N5O3	Unclassified	192.1316, 154.0811, 176.1637, 147.1295, 234.1001, 34.818, 168.1636, 111.0893, 188.062, 140.1033, 159.0669, 73.3389, 173.0712, 196.1066, 223.0476	187.0368, 225.0892, 202.9933, 232.0384, 145.0603, 210.9946, 344.3371, 268.0732, 191.1059, 220.1006, 239.0535, 149.0602, 245.1126
118	*P. lanceolata*	3.4	445.2057	1.43x10^{-15}	[M-H]-	C28H30O5	Hydroxycinnamic acid, glycosylated	208.0767, 206.04, 344.178, 314.1323, 162.0547, 180.0629, 222.0653, 143.8394, 284.1621, 207.082, 132.1511, 224.0723, 354.9964, 178.0425, 385.0764	237.1127, 239.1632, **101.0229**, 131.0749, 283.1466, 265.1399, 223.1375, 301.3603, **161.0265**, 238.1167, 313.0576, 221.1291, 90.2033, 267.1609, 269.1412
119	*P. lanceolata*	3.45	131.0708	3.96x10^{-10}	[M-H]-	C17H18N3	Unclassified	11.8495, 40.1688, 62.2914, 32.8592, 78.4634	90.9014, 119.2207, 68.7789, 98.211, 52.6068

120	*P. lanceolata*	3.46	401.1447	$8.37x10^{-23}$	[M-H]-	C18H26O10	Hydroxycinnamic acid	208.0934, 267.1058, 222.1065	**193.0497,** 134.0357, **179.0368**, 149.0602, 251.0354, *214.0609, 199.017, 209.1159*
121	*P. lanceolata*	3.47	467.155	$4.54x10^{-11}$	[M-H]-	C22H28O11	Hydroxycinnamic acid	330.1244, 228.1005, 288.1134, 186.0789, 258.1007, 272.0794	**137.0259**, 239.0535, **179.0368**, 281.0676, 209.0456, 195.0709
122	*P. lanceolata*	3.55	549.123	$6.05x10^{-22}$			Unclassified		
123	*P. lanceolata*	3.57	481.1363	$8.47x10^{-16}$	[M-H]-	C21H27N2O9P	Unclassified, sulfate/phosphate residue[1]	222.124, 260.0223, 282.1465, 278.026, 240.134	259.0083, 221.1134, 198.9932, 203.1113, 241.0025, *239.1276, **96.9568***
124	*P. lanceolata*	3.64	511.1454	$1.89x10^{-06}$			Unclassified		
125	*P. lanceolata*	3.67	931.2829	$5.98x10^{-13}$	[M-H]-	C42H50N3O21	Terpene, Iridio glycoside	466.1461, 650.2194, 465.1435, 692.23, 794.262	**465.1352**, 281.0676, 239.0535, **137.0259**

126	*P. lanceolata*	3.7	261.0434	$3.01x10^{-08}$	[M-H]-	$C_{14}H_{14}OS_2$	Unclassified, sulfate/phosphate residue[1]	164.08, 18.010, 443.989	**96.9568**, 261.0392, 243.0397
127	*P. lanceolata*	3.72	699.2702	$4.53x10^{-10}$	[M-H]-	$C_{29}H_{48}O_{19}$	Unclassified	476.1707, 386.1521, 478.1494, 180.0629, 520.1596, 404.1653, 548.2029, 434.1592, 564.1513, 506.1483, 418.2034, 475.1701, 374.1337, 433.1501, 494.1764	223.0949, 313.1123, 221.1134, 519.206, 179.1051, 295.1001, 151.0714, 265.1089, 135.1155, 193.1226, 281.0676, 224.0989, 325.1246, 266.1096, 205.0896
128	*P. lanceolata*	3.76	495.1491	$3.12x10^{-24}$	[M-H]-	$C_{21}H_{26}N_3O_{11}$	Unclassified	286.0994, 328.1099, 184.0599, 226.0759, 199.0918, 240.0845, 372.0902	209.0456, 167.0339, 311.0789, 269.0666, 296.0601, 255.0523, 123.0439, *151.0045, 108.0202, 195.0281, 152.0105, 210.053, 139.0405, 137.0619*

129	*P. lanceolata*	3.78	381.175	$3.01x10^{-08}$	[M-H]-	C16H30O10	Flavonoid[1]	184.1287, 132.0413, 187.1729, 220.0928, 188.1116, 171.1144, 189.1036, 210.0648, 175.1943, 246.091, 27.3037, 209.1341, 152.8221, 266.8692, 288.6575	197.0453, 249.135, 194.0057, 161.076, 193.0668, 210.053, 192.0649, 171.1069, 135.0848, 205.9717, 353.8729, 172.0542, 114.3074, 228.3545, […], **151.0413**, […], 317.2094
130	*P. lanceolata*	3.9	415.1597	$1.13x10^{-13}$			Unclassified		
131	*P. lanceolata*	3.94	565.2126	$4.53x10^{-10}$	[M-H]-	C24H38O15	Hydroxycinnamic acid, glycosylated	342.1255, 300.1104, 428.1936, 341.1179, 386.117, 299.1167,	223.0949, 265.1089, **137.0259**, 224.0989, 179.1051, 266.1096, *281.0549,* ***193.0497,*** *221.1134,* *239.0535,* ***119.0474**,* ***179.0368**,* *1**13.0231,*** *241.0025, 191.0368*

132	*P. lanceolata*	3.99	431.1912	$4.41x10^{-09}$	[M-H]-	C17H31N5O6P	Unclassified	226.0665, 225.0701	205.1235, 206.1272
133	*P. lanceolata*	4.01	415.1571	$2.93x10^{-18}$	[M-H]-	C19H28O10	Hydroxycinnamic acid	**44.0226**, 266.1134, 319.5587, 236.0892, 368.3196, 369.0683, 213.0769, 192.0948, 370.3739, 193.9941, 326.7262, 220.0928, 86.1686, 123.0615, 244.0786	371.1343, 149.0453, 95.6, **179.0672**, 46.8418, 46.0904, 202.0768, **223.0655**, 44.7848, 221.165, 88.4325, 195.0709, 328.9901, 292.1031, 255.0892
134	*P. lanceolata*	4.05	465.1398	$9.20x10^{-13}$	[M-H]-	C25H24NO8	Hydroxycinnamic acid amid	180.0629, 328.1099, 329.1172, 224.052, 344.1063, **179.0589**, 300.0852, 184.072	285.0724, **137.0259**, 136.0176, 241.0859, 121.0303, 165.0558, 281.0676

135	*P. lanceolata*	4.07	501.1253	$3.45x10^{-09}$	[M-H]-	C38H16NO	Terpene, Iridio glycoside	216.0379, 364.0792, 215.029, 307.0846, 259.9977, 220.0378, 366.0745, 304.0484, 363.0765, 300.0149, 293.0068, 277.9911, 336.0541, 308.7639, 384.0738	285.0724, **137.0259**, 136.0176, 194.0207, 241.1218, 281.0676, 135.0276, […], 205.0896, […], **113.0231,** […], **465.1352**, […], **291.1985**, **239.0357**
136	*P. lanceolata*	4.18	495.1239	$3.01x10^{-08}$	[M-H]-	C33H20O5	Glycoside, sulfated/phosphorylated	374.1001, 348.0897, 372.0902, 328.0912, 360.1005, 373.0879, 344.1229, 256.0736, **180.0515**, 358.1025, 239.0569, 378.1099, 398.1749, 306.0736, 284.0706	121.0303, 147.0334, 123.0439, 167.0339, 135.0276, 151.0045, 122.0342, 239.0535, 315.0784, 166.0261, **137.0259**, […], **96.9568**

137	*P. lanceolata*	4.31	611.1987	$3.71x10^{-09}$	[M-H]-	C35H32O10	Hydroxycinnamic acid[1]	388.1334, 416.1293, 403.16, 386.117, 401.141, 387.1241, 402.115	**223.0655**, 195.0709, 208.0414, 225.0737, 210.053, 224.0745, 209.0792, *241.0025*
138	*P. lanceolata*	4.48	601.1553	$3.96x10^{-10}$	[M-H]-	C36H26O9	Hydroxycinnamic acid, glycosylated	464.1322, 320.0893, 362.1029, 346.1053, **120.023**, 406.1256, 304.0964, 319.0831, 422.1189	**137.0259**, 281.0676, 239.0535, 255.0523, 481.1337, 195.0281, 297.065, 282.0647, **179.0368**, […], 209.0456, 241.0025, […], 285.0724,
139	*P. lanceolata*	4.48	631.1667	$2.62x10^{-07}$	[M-H]-	C30H32O15	Hydroxycinnamic acid	422.1189, 494.1412, 320.0893, 464.1322, 362.1029, 434.1186, 421.1157, 319.0831, 334.0936	209.0456, 137.0259, 311.0789, 167.0339, 269.0666, 197.0453, 210.053, 312.0897, 297.065, **135.0437**, […], **151.0413**

140	*P. lanceolata*	4.52	343.0853	$3.96x10^{-10}$	[M-H]-	C18H16O7	Flavonoid[1]	133.0131, 234.0514, 150.0187, 283.4155, 122.0194, 125.0103, 22.1424, 90.0397, 14.5222, 109.3641, 129.9858, 45.0353, 166.5308, 147.054, 138.0736	109.0304, 193.0668, 59.6702, 218.0722, 221.065, 320.9433, 253.0499, **283.0318**, 315.2312,
141	*P. lanceolata*	4.55	595.203	$1.19x10^{-17}$	[M-H]-	C35H32O9	Hydroxycinnamic acid, glycosylated	386.117, 372.1338, 401.141, 388.1334, 387.1603, 392.1254, 195.0872, 385.1101, 390.0676, 464.1632, 482.1704, 371.1442, 402.1744, 346.084, 256.0736	209.0792, **223.0655**, 194.0551, **207.0689**, 208.0414, 203.0717, 400.1042, 210.0895, 131.0378, 205.1235, **113.0231**, **101.0229**, […], 285.0724, […], 137.0259

142	*P. lanceolata*	4.62	405.2103	$5.02x10^{-11}$	[M-H]-	C26H30O4	Glycoside	206.04, 129.0568, 229.2584, 240.1509, 135.0641, 339.344, 123.1261, 182.1555, 192.1316, 120.0369, 140.0205, 143.166, 291.1697, 190.1113, 194.1305	199.1759, 276.1589, 175.951, 165.0679, 270.1502, 65.8654, 282.0876, 223.052, 285.1612, […], **113.0231**, […], **207.0689**, [...], 253.0499
143	*P. lanceolata*	4.69	457.2062	$2.60x10^{-10}$	[M-H]-	C29H32NO2S	Unclassified, sulfate/phosphate residue [1]	238.0638, 226.0665, 256.0736, 130.0646, 225.0553, 237.0681, 252.082, 254.0657, 360.244, 296.1033	219.1376, 231.1328, 201.1212, 327.1386, 232.1484, 220.1396, 205.1235, 203.1425, **96.9568**, *161.102, 285.0724, 223.0388*
144	*P. lanceolata*	4.7	235.0292	$4.23x10^{-20}$	[M-H-H]2-, [M-H]-	C31H12N4S	Unclassified	138.0736	96.9568

145	*P. lanceolata*	4.73	459.1889	$3.01x10^{-08}$	[M-H]-	C21H32O11	Flavonoid, glycosylated	240.0587, 160.1377, 258.0849, 262.17, 219.0598, 220.0378, 262.0611, 328.1672, 190.0562, 218.2008, 318.1317, 227.096, 268.2253, 156.074, 114.0704	219.1376, 299.0483, 201.1034, 197.0247, 240.1344, 239.1632, 131.0378, 197.1306, 269.1412, 241.0025, **101.0229**, **284.0313**
146	*P. lanceolata*	4.78	645.1817	$2.62x10^{-07}$	[M-H]-	C38H30O10	Flavonoid	448.1318, 406.1256, 360.1005, 420.1363, 508.1587, 462.1513, 405.1185, 434.1186, 346.1053, 524.1493, 447.135, 404.0961, 494.1412	197.0453, 239.0535, 285.0724, 225.0533, **137.0259**, 183.0269, 240.0608, 211.0615, 299.0802, 121.0303, 198.0467, 241.072, **151.0413**
147	*P. lanceolata*	4.84	487.1812	$1.17x10^{-22}$			Unclassified		
148	*P. lanceolata*	4.85	417.212	$2.37x10^{-08}$			Unclassified		

149	*P. lanceolata*	4.85	585.1615	$6.06x10^{-11}$	[M-H]-	C29H30O13	Hydroxycinnamic acid, glycosylated	300.0852, 448.1401, 304.0964, 346.1053, 406.1256, **120.023**, 299.0826, 344.0761, 303.0885	285.0724, **137.0259**, 281.0676, 239.0535, **179.0368**, **465.1352**, 241.0859, 136.0176, 282.0647, *303.0888, 240.0608, 209.0456*
150	*P. lanceolata*	4.91	467.2123	$4.54x10^{-11}$			Unclassified		
151	*P. lanceolata*	4.92	421.1171	$3.96x10^{-10}$	[M-H]-	C13H26O15	Hydroxycinnamic acid, glycosylated	224.0723, 213.0769, 239.1095, 198.054, 324.1594, 282.0802, 268.0644, 214.046, 219.0598	197.0453, 208.0414, 182.0217, **223.0655**, **96.9568**, 139.0405, 153.0575, **207.0689**, […], *241.0025*,
152	*P. lanceolata*	4.96	429.1762	$1.93x10^{-13}$	[M-H]-	C18H31N4O4PS	Unclassified	190.043, 224.052, 234.0315, 180.0629, 204.066, 233.0387, 189.0382, 230.1554, 268.0376, 186.0566, 223.0476,	239.1276, 205.1235, 195.1405, 249.111, 225.11, 240.1344, 199.017, 161.1349, 243.1191, 206.1272, 207.0908

								222.0789, 316.1603	
153	*P. lanceolata*	4.99	236.0371	$9.04x10^{-16}$	[M-H-H]2-	C16H26O12S2	Unclassified	139.0782	96.9568
154	*P. lanceolata*	5.22	517.1903	$5.21x10^{-12}$	[M-H]-	C23H34O13	Glycoside	404.1653, 240.0135, 183.9854, 356.1414, 239.0144, 380.1701, 368.1802, 297.1105, 406.1852, 388.1716, 317.1711	**113.0231**, 277.1859, 333.2139, **161.0446**, **137.0259**, 149.0142, 220.0741, 111.0098, 129.0167, 200.0144, *241.0025*
155	*P. lanceolata*	5.28	443.1913	$2.43x10^{-17}$	[M-H]-	C21H32O10	Hydroxycinnamic acid, glycosylated	330.1763, 312.1657, 206.3121, 327.1493, 308.1546, 346.166, 299.1693, 398.0877, 132.0413, 270.2668, 240.0587, 380.1226, 198.054, 180.1778, 332.1922	**113.0231**, 131.0378, 236.8825, 116.0436, **135.0437**, 97.0278, 144.0211, 45.1113, 311.153, 172.9278, 203.1425, 63.0727, 263.0289, 111.0098, 245.1308

156	*P. lanceolata*	5.35	711.3941	2.93×10^{-18}	[M-H]-	C38H56N4O9	Unclassified	208.0553, 207.0486	503.3365
157	*P. lanceolata*	5.39	303.0538	5.98×10^{-13}	[M-H]-	C12H16O7S	Unclassified, sulfate/phosphate residue[1]	206.1074	**96.9568**, 303.063
158	*P. lanceolata*	5.51	427.1967	1.42×10^{-15}	[M-H]-	C21H32O9	Hydroxycinnamic acid amid[1]	224.052, 180.0629, 292.1142, 226.0665, 223.0476, **179.0589**	203.1425, 247.1363, 135.0848, 201.1212, 248.1343
159	*P. lanceolata*	5.64	473.2015	2.62×10^{-07}	[M-H]-	C22H34O11	Hydroxycinnamic acid, glycosylated	270.0571, 226.0665, 338.1138, 376.2461, 208.0553, 225.0701, 270.1182, 272.0649, 354.1518, 372.6861, 352.1338, 297.6142, 395.5417, 342.1565, 269.0468	203.1425, 247.1363, 135.0848, **96.9568**, 265.1399, 248.1343, 203.0854, 201.1212, **119.0474**, 100.5144, 121.0666, ***161.0446***
160	*P. lanceolata*	5.65	393.1218	3.45×10^{-09}	[M-H]-	C23H24NOS2	Unclassified, sulfate/phosphate residue[1]	152.1228, 136.1242, 296.1629, 154.127, 44.0731	241.0025, 393.1141, 257.0019, **96.9568**, 239.0009, 349.0557

161	*P. lanceolata*	5.89	499.2544	$3.90x10^{-09}$	[M-H]-	C25H40O10	Jasmonate derivative, glycosylated	208.0553, 263.1739, 335.1664, 236.0404, 123.1576, 286.2019, 362.2213, 270.0957, 200.3785, 307.2391, 328.958, 306.1887, 385.2589, 296.2336, 328.1997	**291.1985,** 236.0884, 164.0758, 263.2105, 376.081, 137.0259, 213.0539, 192.0115, 229.1565, 298.8654, 170.2973, 193.0668, 203.0174, **113.9943**, 113.0231
162	*P. lanceolata*	6.02	327.0906	$1.37x10^{-14}$	[M-H]-	C12H24O6S2	Unclassified	104.0434, 103.0463, 118.0644, 87.0185	239.0709, 223.0388, 224.0455, 209.0222, 240.0608
163	*P. lanceolata*	6.08	443.1919	$3.79x10^{-10}$	[M-H]-	C19H30N3O9	Unclassified	256.0868, 180.0722, 224.0607	187.1029, 263.1313, 219.1376
164	*P. lanceolata*	6.67	695.3998	$4.54x10^{-11}$	[M-H]-	C30H58N5O13	Unclassified	208.0553, 207.0486	487.3394, 207.1389
165	*R. acris*	1.42	355.1239	$7.93x10^{-15}$	[M-H]-	C13H24O11	Glycoside[1]	240.0845, 78.0308, 254.1077	115.039, 277.0888, **101.0229**
166	*R. acris*	2.11	315.1074	$7.93x10^{-15}$			Unclassified		

167	*R. acris*	2.59	513.1242	$8.91x10^{-08}$	[M-H]-	C21H24O12	Unclassified	314.0478, 270.0571, 296.0371, 313.0423, 358.04, 298.011	
168	*R. acris*	2.74	137.0244	$6.51x10^{-07}$	[M-H]-	C4H10O3S	Unclassified	43.989	93.0304
169	*R. acris*	2.88	375.1288	$8.91x10^{-08}$	[M-H]-	C23H20O5	Hydroxycinnamic acid	178.0813, 166.0885, 241.0909, 164.0621, 177.0753, 252.082, 268.0747, 179.0983, 182.0937, 165.0762, 176.0582, 224.1105, 222.0377, 208.0553, 254.1748	197.0453, 209.0456, 134.0357, 211.0615, 198.0467, 123.0439, 107.0499, 196.0308, **193.0497**, 153.0918, […], **137.0259**, […], 207.0908
170	*R. acris*	2.99	477.1606	$1.02x10^{-09}$	[M-H]-	C27H26O8	Hydroxycinnamic acid, glycosylated	364.1365, 208.0553, 316.1063, 232.092, 352.1338, 334.1267, 376.1352, 207.069, 276.1007,	**113.0231**, 269.1031, **161.0446**, 245.0666, 125.0251, 143.0333, **101.0229**, 270.0875, 201.0544, 263.0875

								214.0638, 332.1016, 233.1578, 265.0149, 385.0764, 273.2598	, *[…],* ***355.0771,*** *[…], 191.0368, […]*
171	*R. acris*	3.4	327.1082	$1.90x10^{-23}$			Unclassified		
172	*R. acris*	3.65	405.2115	$6.51x10^{-07}$			Unclassified		
173	*R. acris*	3.73	403.1955	$9.10x10^{-11}$	[M-H]-	C19H32O9	Hydroxycinnamic acid[1]	180.0629, **179.0589**	223.1375, 403.2004
174	*R. acris*	3.84	437.2375	$2.80x10^{-10}$	[M-H]-	C13H36N5O11	Unclassified	311.2297, 245.0832, 232.1056, 299.1693, 262.1517, 244.163, 279.1605, 199.1304, 180.1089, 309.3672, 294.1511, 300.1765, 155.0775, 258.1194, 214.1384	126.0036, 192.1501, 205.1235, 138.0614, 175.0776, 193.0668, 158.068, 257.1197, 238.1, 127.8661, 137.0619, […], 332.1799, 285.1101

175	*R. acris*	3.89	435.2223	$5.30x10^{-14}$	[M-H]-	C20H36O10	Glycoside, acidified	189.134, 322.1988, 216.1147, 246.1423, 166.2978, 250.1509, 248.1118, 268.1292, 210.0648, 298.1878, 123.0935, 169.1362, 286.2019, 232.2052, 296.2336	**113.0231**, 246.0869, 219.1092, 189.0853, 268.9209, 185.071, 187.1029, 167.0902, 225.1551, **137.0259**
176	*R. acris*	3.98	515.1759	$8.34x10^{-12}$	[M-H]-	C13H34N5O14S	Unclassified	312.1053, 294.0955, 324.1021, 311.1003	203.0717, 221.0818, 191.0681, 204.0741
177	*R. acris*	4.13	383.1389	$6.17x10^{-11}$	[M-H]-	C16H22N3O8	Hydroxycinnamic acid amid[1]	162.0547, 180.0629, 161.0481, **179.0589**	221.0818, 203.0717, 222.0885, 204.0741, *191.0681*
178	*R. acris*	4.2	403.1956	$1.45x10^{-21}$			Unclassified		
179	*R. acris*	4.47	405.1769	$6.17x10^{-11}$	[M-H]-	C18H30O10	Unclassified	180.0629, 182.0445, 224.052, 181.0362	225.11, 223.1375, 181.1207, *403.2004, 207.0908*

180	*R. acris*	4.79	427.196	6.51x10^{-07}	[M-H]-	C11H34N5O10S	Unclassified	206.1074, 224.1315, 224.052, 266.0616, 223.1161, 223.0476	221.0818, 203.0717, 203.1425, 161.1349
181	*R. acris*	4.83	383.1283	6.51x10^{-07}			Unclassified		
182	*R. acris*	4.84	429.1684	3.87x10^{-07}	[M-H]-	C27H26O5	Glycoside, acidified	190.043, 234.0315, 186.1345, 224.0393, 188.0227, 226.0217, **180.0515**, 208.0767, 242.0555, 189.9641, 322.1142, 268.0376, 44.0413, 189.0382, 316.1716	239.1276, 195.1405, 243.0397, 205.1235, 241.1415, 203.1425, 249.111 , […], 119.4981, **137.0259**
183	*R. acris*	5.24	269.0107	1.39x10^{-20}	[M-H]-	C8H7N4O5P	Unclassified	*71.9859*	269.0075
184	*A. pratensis*	3.31	347.0439	1.30x10^{-08}	[M-H]-	C16H12O9	Hydroxycinnamic acid	153.9984, 169.0232, 152.9927, 213.0146	**193.0497**, **178.0276**, 194.0551, 134.0357, 149.0602

185	*A. pratensis*	3.77	197.0452	$4.74x10^{-07}$	[M-H]-	C7H8N3O4	Unclassified, methoxylated	74.0398, **30.0455**, 76.0075, 91.0479, 15.0225	123.009, 166.9979, 121.0303, 105.9976
186	*A. pratensis*	4.43	389.1232	$6.05x10^{-06}$			Unclassified		
187	*A. pratensis*	5.17	477.1801	$7.31x10^{-07}$	[M-H]-	C24H30O10	Flavonoid, glycosylated	236.177, 380.2189, 176.1637, 228.1218, 252.1681, 192.1447, 278.1856, 235.1754, 140.1033, 254.1907	241.0025, **96.9568**, 301.0129, 249.0584, 225.0151, **285.0426**, 198.9932, 242.003, […], **207.0689**, […], 259.0083, **113.0231**
188	*A. pratensis*	7.42	557.2409	$1.55x10^{-06}$	[M-H]-	C25H40N3O9S	Unclassified		-
189	*A. pratensis*	3.15	445.1711	$7.15x10^{-07}$	[M-H]-, [M+HCOOH-H]-	C20H30O11	Hydroxycinnamic acid	240.098, 326.1145, 314.1323, 330.1244, 260.1647, 222.0653, 204.0467, 344.1475, 248.1248, 232.1343, 238.1944, 322.124, 202.0427,	205.0667, **119.0474**, 131.0378, 115.039, 185.0035, 223.1113, 241.1218, **101.0229**, 197.0453, 213.0379, 206.9821, 123.0439, 243.1191, **193.0497**, 96.9568,

								264.0997, 252.1182	
190	*A. elatius*	5	567.3523	$2.03x10^{-11}$	[M-H]-	C28H50N5O5S	Unclassified, aliphatic acid[1]	**46.0007**, 324.2067, 212.1099, 342.2376, 44.986, 330.2911, 371.258, 160.1377, 351.2545, 234.1441, 426.2234, 346.2339, 296.2557, 412.2794, 232.2547, 268.2253, 374.2232, 397.284, 239.2619, 419.27, 251.2875, […], 322.2388	521.3444, 243.1355, 355.2324, 225.11, 522.3602, 237.0561, 196.0895, 407.2, […], 271.1001, […], 193.1226, […], 277.1859, […], 335.222, 86.7158, 203.0356, 223.1375
191	*A. elatius*	5	815.4404	$3.27x10^{-08}$	[M-H]-	C42H64N4O12	Unclassified	178.0517, 310.0917, 177.0455, 309.0887	637.3967, 505.3441

192	*A. elatius*	5.15	683.3985	$2.79x10^{-09}$	[M-H]-	C36H60O12	Hydroxycinnamic acid	**46.0007**, 366.1566, 552.3563, 440.2834, 44.986, 414.3207, 514.3099, 462.298, 458.288, 262.13, 526.3577, 412.3319, 534.3425, 468.3587, 354.2501	637.3967, 317.2382, 131.0378, 243.1191, 269.0666, 169.0875, 221.0969, 225.11, 421.2697, 157.0404, **271.0641**, 149.0453, […], 280.0651, 119.0359, 195.0709, 161.011, 356.2882, 507.3065, 202.0964, 215.124, 373.2316, 217.1001, 111.0098, 199.1314, 127.0411, 64.5409, 188.0663, […], 414.2356, 553.2325, 505.3441, 104.5848, 195.2681, 330.0706, 339.1384, 638.301, **223.0655**, 205.1235, 68.0812

193	*A. elatius*	5.25	681.3819	$3.84x10^{-07}$	[M-H]-	C36H58O12	Glycoside	324.2403, 467.8713, 568.3635, 384.2952, 461.3946, 458.288, 454.3821, 472.2283, 484.3326, 448.4163, 468.3058, 552.3361, 474.309, 527.3238, 564.3278, 567.3276, 234.196	357.148, 213.5164, **113.0231**, 297.0916, 219.9961, 209.1516, 197.0573, 223.0949, 227.0132, 129.0568, 213.0765, 232.9686, **207.0689**, 117.054, 114.0562
194	*A. elatius*	5.38	565.3367	$3.84x10^{-07}$	[M-H]-	C27H54N2O6S2	Unclassified, aliphatic acid[1]	292.1493, 234.1118, 76.0075, 291.1441, 248.1248, 242.0909, 124.039, 344.2492, 178.0674, 396.2479, 166.0885, 344.1991, 75.0144, **46.0007**	273.1844, 331.2252, 489.3266, 274.1883, 317.2094, 323.2398, […], 329.2154, 369.2372, 121.0666, 272.0955, 271.1689, […], 521.3444, […] 149.0602, […], 223.1375

195	*A. elatius*	5.57	565.3362	$2.32x10^{-08}$	[M-H]-	C29H48N3O8	Unclassified, aliphatic acid	292.1493, 160.0532, 130.0401, 234.1001, **46.0007**, 78.0162, 76.0075, 225.2359, 352.2007, 142.0402, 291.1441, 210.2094, 204.0774, 262.13, 94.0213	273.1844, 405.2783, 435.2854, 331.242, 519.335, 487.3098, 489.3266, 340.087, 213.13, 423.29, 274.1883, 355.1211, 361.245, 303.1995, […], 323.2398, 317.2094, 329.2154
196	*A. elatius*	5.74	341.1073	$1.01x10^{-06}$			Unclassified		
197	*A. elatius*	5.93	563.3213	$3.27x10^{-08}$	[M-H]-	C33H46N3O3S	Unclassified	76.0075, 248.1118, 75.0009, 43.9742, 41.965, 106.0016, 46.0233, 260.1117, 424.2433, 234.0893, 62.0082, 138.0285, 246.1031, 186.0789, 290.1257	487.3098, 315.2042, 519.335, 521.3444, 457.3035, 517.2826, 518.3384, 303.1995, 139.0677, 329.2154, 501.2964, 425.2881, 317.2094, […], 316.1873, 239.1276, 205.1235, [...], 245.1126

198	*A. elatius*	5.99	461.2202	$2.49x10^{-06}$	[M-H]-	C22H30N3O5	Unclassified	329.1921, 252.1023, 204.0774, 264.1511	132.0295, 209.1159, 257.1437, 197.0713
199	*A. elatius*	6.05	551.3564	$1.07x10^{-15}$	[M-H]-	C29H50N3O7	Unclassified, aliphatic acid	**46.0007**, 44.986, 110.046, 212.1099, 218.1015, **43.9987**	505.3441, 441.3025, 339.2311, 333.2504, *507.3547, 287.2074, 261.1478, 327.218, 289.2173, 417.2452, 315.2312, 101.0535, 419.2563, 506.4809, 277.1309*
200	*A. elatius*	6.4	551.3568	$1.07x10^{-15}$	[M-H]-	C28H48N3O5	Unclassified, aliphatic acid	130.0401, **46.0007**, 44.9997, 129.0391, 236.1211, 286.1751, 78.0308, 48.0118, 338.2234, 268.167, 148.0624, 266.1499, 285.1717, 296.1994, 64.0136	421.3025, 505.3441, 315.2312, 265.1819, 473.3278, 503.3365, 213.13, 283.1942, 403.2943, 285.2004, 255.1592, 487.3394, 284.1947, 355.261, 298.1401

201	*A. elatius*	6.69	549.3393	$1.34x10^{-15}$	[M-H]-	C30H52N3O2S2	Unclassified, aliphatic acid	**46.0007**, **43.989**, 44.9997, 234.1118	503.3365, 505.3441, 315.2312
202	*A. elatius*	6.79	533.3115	$3.84x10^{-07}$	[M-H]-	C24H48N5O4S2	Unclassified, aliphatic acid[1]	216.0961, 106.0686, **46.0007**, 232.1175, 268.1292, 318.2188, 410.2298, 286.1751, 122.0631, 242.1361, 163.0633, 15.9992, 170.0707, 209.0958, 187.0831	317.2094, 427.243, 487.3098, 301.1902, 265.1819, 215.1011, 123.0819, 247.1363, 411.2511, 291.1644, 370.244, 517.3189, […], 149.0602, […], *153.0918*
203	*A. elatius*	6.84	565.3362	$1.47x10^{-13}$	[M-H]-	C29H48N3O8	Terpene, Sesqueterpene[1]	262.13, 76.0075, **46.0007**, 267.1899, 222.1065, 94.0213, 75.0144, 112.0342, 261.1299, 44.9997, 248.1248, 380.2189, 210.1013, 314.1683, 106.0239	303.1995, 489.3266, 519.335, 298.1401, 343.2291, 471.3123, 453.305, 304.2075, 317.2094, 185.1131, 355.2324, […], 329.2154, […], 383.2587, 413.2626, 521.3444

204	*A. elatius*	7.18	549.3422	$1.26x10^{-14}$	[M-H]-	C28H46N3O5	Terpene, Sesqueterpene, methoxylated[1]	130.0401, 45.9861, 78.0308, 262.1167, 306.1887, 44.9997, 47.9903, 220.1206, 210.1013, 222.124, 364.1855, 390.9772, 308.1939, 488.6523, 273.1702	419.2905, 503.3365, 471.3123, 287.2074, 243.15, 501.3325, 329.2154, […], 315.2312,, […], 401.2742, **383.2587**
205	*A. elatius*	7.28	545.3097	$2.79x10^{-09}$	[M-H]-	C21H46N4O12	Terpene, Sesqueterpene[1]	41.965, 43.9742, 144.0263, 126.0133, 202.0772, 258.1007, 76.0075, 234.1118, 216.0961, 384.3476, 192.0659, 292.1493, 232.092, 218.0786, 344.1991	503.3365, 501.3325, 401.2742, 419.2905, […], 469.3009, 311.1974, 329.2154, 160.9552, 353.2457, 253.1635, 313.2146, 327.218, 201.1212, […], 239.1276, […], 153.1273, **383.2587**

206	*A. elatius*	7.29	517.3155	2.32x10^{-08}	[M-H]-	C30H46O7	Jasmonate derivative[1]	204.0979, 80.009, 186.0789, 98.0194, 230.1057, 214.0824, 304.1743, 284.1494, 61.9988, 252.1182, 242.1054, 188.0931, 96.0272, 302.1653, 18.0104	313.2146, 437.3018, 331.2252, 419.2905, 287.2074, 303.2249, 213.13, 233.1619, 455.3146, 265.1819, 275.2032, 329.2154, 421.2697, 215.1512, **263.1705**
207	*A. elatius*	7.54	547.3261	2.19x10^{-09}	[M-H]-	C30H48N2O5S	Unclassified	**43.989**, 338.1971, 42.9854, 260.1117, 230.1057, 128.023, 258.1007, 312.183, 203.0903, 350.1924, 274.1389, 246.091, 248.1475, 44.9997	503.3365, 209.1159, 287.2074, 317.2094, 419.2905, 289.2173, 235.134, 344.2309, 197.1306, 273.1844, 301.2162, 329.2154,

208	*A. elatius*	7.63	533.345	$2.32x10^{-08}$	[M-H]-	C30H48N2O5S	Unclassified	190.1113, 242.1054, 189.1171, 164.1048, 264.133, 280.1259, 272.1921, 107.0383, 46.0095, 121.0477, 132.1096, 314.1179, 126.0566, 391.234, 108.0228	343.2291, 291.2381, 344.2309, 369.2372, 269.2125, 253.2208, 261.1478, 426.3014, 487.3394, 412.3063, 401.229, 219.2272, 142.118, 407.2874, 425.3276
209	*A. elatius*	7.73	651.408	$2.79x10^{-09}$	[M-H]-	C36H54N4O4	Unclassified	438.3059, 442.2545, 342.1982, 348.1771, 389.242, 316.1886, 386.2276, 368.1802, 434.2931, 292.2296, 320.2811, 359.2915, 439.2593, 184.1096, 120.1707	213.1007, 209.1516, 309.1961, 303.2249, 262.1675, 335.222, 265.1819, 283.2213, 217.1001, […], 119.0359, 239.1632, 284.1947, 313.2146, […], 235.1667, 205.1235, […], 305.1755
210	*A. elatius*	7.74	501.3198	$2.32x10^{-08}$	[M-H]-	C23H54N2O3S3	Unclassified	304.1572	197.1636

211	*A. elatius*	8.19	537.3245	$3.27x10^{-08}$	[M-H]-	C30H50O6S	Unclassified		-
212	*A. elatius*	8.32	545.3111	$9.23x10^{-06}$	[M-H]-	C32H50O3S2	Unclassified	392.2177, 232.092, 334.1379, 216.0961, 89.9852, 306.1406, 204.0979, 258.1007, 350.1707, 290.1547, **43.9987**, 202.0772, 234.0893, 328.2884, 408.2147	153.0918, 313.2146, 211.1654, 329.2154, 455.3146, 239.1632, 341.2086, 287.2074, 328.2169, 195.1405, 255.1592, 501.2964, 343.2291, 311.228, 217.0273
213	*A. elatius*	8.88	535.3098	$3.84x10^{-07}$	[M-H]-	C30H48O6S	Unclassified		-
214	*A. elatius*	9.18	529.3155	$2.32x10^{-08}$			Unclassified		
215	*A. elatius*	9.92	517.3519	$3.84x10^{-07}$	[M-H]-	C30H50N2O3S	Unclassified, sulfate/phosphate residue, aliphatic acid	220.2027, 247.2371, 421.3033, 298.3552, 206.1554, 45.0353, 242.2199, 424.1264, 258.1731, 294.1686,	297.1417, 270.1163, 96.0502, 218.9983, 311.1974, 275.123, 472.3191, 93.2271, 200.1165, 223.1853, 259.1748, 273.1844, **96.9568**,

								317.2381, 244.163, **46.0007**	[...], 245.1126, 201.0119,
216	*L. perenne*	5.59	305.1389	$9.06x10^{-07}$	[M-H]-	C17H22O5	Hydroxycinnamic acid[1]	96.0272, 198.0828, 103.0652, 80.009, 102.0227, 18.0104, 194.0539, 142.1069, 144.0754, 113.1488, 100.0532, 102.0702, 156.1176, 230.9023, 90.0052	209.1159, 305.1357, 107.0499, 202.0768, 225.1254, 203.1113, 287.1273, 111.0818, **163.0379**, 161.0601, 191.9791, 205.0896, 303.2521, 203.0717, 149.0142

217	*P. pratensis*	4.5	453.086	$5.13x10^{-06}$	[M-H]-	C23H18O10	Glycoside[1]	123.9555, 168.9858, 241.9507, 326.0192, 216.0104, 364.5956, 235.015, 339.0741, 337.0241, 167.9776, 168.032, 317.0434, 256.0736, 234.9089, 344.0579	329.1413, 284.1041, 211.1362, 127.0707, 237.0779, 88.5034, 218.0722, 114.0154, 116.07, 136.0447, 285.1101, […], 313.1123, […], **163.0702**

[1] annotation bases on one characteristic fragment or neutral loss

Supplementary Table 7: Identifier fragments and characteristic masses. The table contains all neutral losses and fragment ions which were used for classification of features as compounds in Supplementary Table 6.

**identifier fragments**	**masses (m/z)**
neutral losses	
Mehtyl residue 15.0225	15.0225
CO residue 27.9912	27.9912
Methoxylated aromatic compounds 30.0455	30.0455
Polyketides 42.0168	42.0168
CO2 residue 43.9890	43.989
CO2 residue 43.9987	43.9987
2-hydroxylated (Propyl)Chromones 44.0226	44.0226
Aliphatic acid 46.0007	46.0007
Glycoside C-Glycoside 120.023	120.023
Hexose 162.0418	162.0418
Hydroxycinnamic acid amid (C9H9NO3) 179.0589	179.0589
Hexose 180.0515	180.0515
Fragment ions	
Sulfate fragment (HSO4-)/Phosporous group (H2PO4-) 96.9568	96.9568
Carbohydrates fragment 101.0229	101.0229
Carbohydrates fragment 113.0231	113.0231
Coumaroyl fragment 119.0474	119.0474
Caffeoyl fragment 135.0437	135.0437
Salicylate fragment 137.0259	137.0259
Caffeoyl fragment 145.0209	145.0209
Myricetin fragment 151.0413	151.0413
Coumarine fragment 161.0265	161.0265
Desoxyglycosylated fragment 161.0446	161.0446
Coumaroyl fragment 163.0379	163.0379
Rhamnoside fragment 163.0702	163.0702
Sinapoyl fragment 164.0454	164.0454
Ferulic acid fragment 178.0276	178.0276
Caffeoyl fragment 179.0368	179.0368
Sinapoyl fragment 179.0672	179.0672
Quinic acid fragment 191.0543	191.0543
Ferulic acid fragment 193.0497	193.0497
Lauric- acid 199.1759	199.1759
Fraxetin fragment 207.0349	207.0349
1-O-methyl-β-D-glucuronate fragment 207.0689	207.0689
Sinapic acid fragment 223.0655	223.0655
Aglycon of sesquiterpene glycosides 239.0357	239.0357
Kaempferol backbone fragment 255.0294	255.0294

Jasmonate fragment 263.1705	263.1705
Naringenine fragment 271.0641	271.0641
Cyanidin fragment 283.0318	283.0318
Kaempferol fragment 284.0313	284.0313
Kaempferol fragment, Anthocyanidine-backbone 285.0426	285.0426
Jasmonate fragment 291.1985	291.1985
Disaccharide fragment 323.0905	323.0905
Esculetin(4-O-8)G fragment 355.0771	355.0771
Sesqueterpene fragment 383.2587	383.2587
Iridoid glycoside fragment 465.1352	465.1353

Supplementary Table 8: **Individual explained variance of the semi-polar metabolite composition of exudates by single variables of the factors LNH, Soil and Climate**. Table contains the explained amount of variance (in %) of semi-polar metabolites by different environmental variables. A detailed description of the abbreviation is given in Supplementary Table 10. Residuals = remaining unexplained variation.

	Environ-mental factor and included single variables (SV)	Species	Plot	SV	Species +Plot	Plot +SV	SV +Species	Species +Plot +SV	Residuals
forb	LNH	19.60	9.22	0.03	0.00	0.28	0.03	0.10	75.10
	- Cover	19.79	9.22	0.12	0.00	0.28	0.00	0.06	75.01
	- Richness	19.60	9.22	0.03	0.00	0.28	0.03	0.10	75.10
	- Shannon	19.76	9.59	0.19	0.00	0.00	0.00	0.08	74.94
	LUI	19.64	9.74	0.34	0.00	0.00	0.00	0.00	74.79
	- fertilization	19.68	9.77	0.37	0.00	0.00	0.00	0.00	74.76
	- grazing	19.64	9.74	0.34	0.00	0.00	0.00	0.00	74.79
	- mowing	19.77	9.53	0.01	0.00	0.00	0.00	0.11	75.12
	Soil	19.67	9.34	0.00	0.00	0.15	0.00	0.07	75.36
	- pH	19.73	9.39	0.00	0.00	0.11	0.00	0.03	75.37
	- TC	19.74	8.95	0.00	0.00	0.54	0.00	0.21	75.16
	- TN	19.50	8.69	0.00	0.00	0.80	0.13	0.00	75.22
	- moisture	19.57	9.21	0.05	0.00	0.29	0.06	0.04	75.08
	- soil core	19.73	9.39	0.00	0.00	0.11	0.00	0.03	75.37
	- soil type	19.47	9.54	0.15	0.00	0.00	0.16	0.00	74.98
	Climate	19.66	9.09	0.05	0.00	0.41	0.00	0.02	75.08
	- precipitation	19.60	9.22	0.03	0.00	0.28	0.03	0.10	75.10
	- T(10)	19.79	9.22	0.12	0.00	0.28	0.00	0.06	75.01
	- T(200)	19.60	9.22	0.03	0.00	0.28	0.03	0.10	75.10
grass	LNH	2.89	13.13	0.10	0.00	0.35	0.00	0.09	84.25
	- Cover	2.78	12.96	0.00	0.00	0.51	0.04	0.00	84.45
	- Richness	2.89	13.13	0.10	0.00	0.35	0.00	0.09	84.25
	- Shannon	2.60	13.84	0.52	0.00	0.00	0.21	0.00	83.83
	LUI	2.71	13.90	0.96	0.00	0.00	0.11	0.00	83.39
	- fertilization	2.69	13.87	1.12	0.00	0.00	0.12	0.00	83.23
	- grazing	2.71	13.90	0.96	0.00	0.00	0.11	0.00	83.39
	- mowing	2.76	13.29	0.11	0.00	0.19	0.05	0.00	84.24

Soil	2.89	13.90	0.39	0.00	0.00	0.00	0.06	83.96
- pH	2.82	13.88	0.42	0.00	0.00	0.00	0.00	83.94
- TC	2.51	13.49	0.57	0.00	0.00	0.31	0.00	83.78
- TN	2.78	12.71	0.00	0.00	0.76	0.04	0.00	84.42
- moisture	2.83	13.11	0.00	0.00	0.37	0.00	0.05	84.37
- soil core	2.82	13.88	0.42	0.00	0.00	0.00	0.00	83.94
- soil type	2.85	13.44	0.00	0.00	0.04	0.00	0.06	84.37
Climate	2.86	11.62	0.00	0.00	1.85	0.00	0.05	84.66
- precipitation	2.89	13.13	0.10	0.00	0.35	0.00	0.09	84.25
- T(10)	2.78	12.96	0.00	0.00	0.51	0.04	0.00	84.45
- T(200)	2.89	13.13	0.10	0.00	0.35	0.00	0.09	84.25

Supplementary Table 9: List of samples. Table lists the number of samples per growth form or species and site, respectively.

	ALB	HAI	SCH
forb	53	61	24
grass	52	41	31
A.millefolium	11	13	4
G.mollugo	12	12	7
G.verum	12	14	5
P.lanceolata	10	10	5
R.acris	8	12	3
A.pratensis	5	8	4
A.elatius	14	6	5
D.glomerata	14	11	9
L.perenne	10	7	7
P.pratensis	9	9	6

Supplementary Table 10: List of used environmental factors and included variables including abbreviations, category, unit and description.

Environmental factors and variable	Abbreviation	category	Unit
Local neighbouring plant community	LNH		
Covered area by neighbouring plants	Cover		%
Richness of each species occurring around the target plant	Richness		
Species diversity of neighbouring plant community	Shannon	LNH	
Land use intensity index	LUI		-
Annual grazing frequency	Grazing		livestock units days of grazing*ha^{-1}*$year^{-1}$
Annual mowing frequency	Mowing		times* $year^{-1}$
Annual fertilization intensity	Fertilization	LUI	kg N*ha^{-1} *yr^{-1}
Relative annual humidity	humidity		%
Annual temperature in 10 cm height	T (10)		°C
Annual temperature in 200 cm height	T (200)	Climate	°C
pH of the soil	pH		-
Soil moisture	moisture		%
Total soil carbon content	TC		%
Total soil nitrogen content	TN		%
Soil group	Soil group		-
Soil type	Soil type	Soil	-
